# Integrating vocational rehabilitation and mental healthcare to improve the return-to-work process for people on sick leave with stress-related disorders: results from a randomized trial

**DOI:** 10.5271/sjweh.4021

**Published:** 2022-06-30

**Authors:** Andreas Hoff, Jonas Fisker, Rie Mandrup Poulsen, Carsten Hjorthøj, Nicole Kristjansen Rosenberg, Merete Nordentoft, Anders Bo Bojesen, Lene Falgaard Eplov

**Affiliations:** 1 Copenhagen Research Institute for Mental Health [CORE], Mental Health Services Capital Region of Denmark, Copenhagen, Denmark; 2 The National Board of Social Services, Odense C, Denmark; 3 University of Copenhagen, Department of Public Health, Section of Epidemiology, Copenhagen, Denmark; 4 Mental Health Center Copenhagen, Mental Health Services Capital Region of Denmark, Copenhagen, Denmark

**Keywords:** adjustment disorder, common mental disorder, distress, exhaustion disorder, integrated service, stepped care

## Abstract

**Objective:**

Stress-related disorders are common, associated with substantial individual suffering, and place a large economic burden on society. While treatment appears to be able to reduce symptoms, evidence of interventions to improve vocational outcomes is flimsy. Lack of integration of vocational rehabilitation and healthcare services has been suspected to be a major potential barrier in return-to-work (RTW) processes; therefore, we aimed to test the effectiveness of such integration.

**Methods:**

We randomized participants who were on sick leave for ≥ 4 weeks with a stress-related disorder. They were allocated to (i) service as usual (SAU), (ii) improved mental healthcare (MHC), or (iii) integrated interventions (INT). The primary outcome was RTW rates measured at 12 months. Secondary outcome were RTW rates measured at 6 months, proportion in work at 12 months, and levels of stress, anxiety, depression, and functioning at 6 months.

**Results:**

We included 666 participants. On the primary outcome and almost all other vocational outcomes, SAU was superior to both INT and MHC. MHC and INT did not differ on any vocational outcome. On several symptom scales, MHC showed lower values than SAU, whilst INT did not differ from the two other groups.

**Conclusion:**

Both the INT and the MHC intervention lowered RTW rates compared with SAU, and thereby yielded a worse outcome. However, the MHC group showed a tendency towards having lower symptom levels compared with those in the SAU group; accordingly, the SAU group is not unequivocally superior. MHC and INT showed no general differences.

Stress-related disorders, including adjustment disorders and exhaustion disorders, are common mental disorders (CMD), which are frequent and account for a large and increasing part of long-term sick leave from work (1). CMD are associated with substantial individual suffering and place a large economic burden on society due to the expense of sick-leave benefit, treatment and rehabilitation, as well as loss of production (2).

For most people, paid employment is beneficial because it involves a steady income, daily structure and a sense of purpose (3). Long-term sickness absence is associated with risk of permanent exclusion from the labor market, and permanent exclusion is associated with poor mental health and depression (4). Therefore, a hastened return to work (RTW) is considered positive. Nevertheless, 20–30% of absentees experience recurrent sick leave, and professionals and sickness absentees are often uncertain of what is the right time for each individual to return to work (5).

No clear consensus exists about best practice treatment for stress-related disorders. Although interventions such as Mindfulness-Based Stress Reduction (MBSR) and Mindfulness-Based Cognitive Therapy (MBCT) have shown positive results in reducing symptoms (6), studies testing an intervention effect on vocational outcomes have shown heterogenous findings. A recent systematic review concluded that a positive impact on RTW was seen in interventions with multiple intervention components and these should preferably include work-place contact or enhanced use of graded RTW (7). Often, these interventions are not sufficiently coordinated, and this lack of service integration is perceived as a stressful barrier for persons on sick leave (8). From studies targeting persons with severe mental disorders, sector integration has shown positive vocational results (3). For CMD alone, two studies have been undertaken: one with positive (9) and one with negative results (10). Hence, evidence remains scarce on the impact of sector integration when targeting CMD-associated sick-leave, and OECD has called for more investigation in this area (2).

The objective of this randomized trial was to compare the IBBIS Integrated Intervention (INT), with two other interventions, both lacking such integration: (i) a mental healthcare (MHC) group – an investigator-directed control group consisting of standard municipal vocational rehabilitation and best practice mental healthcare – and (ii) a non-investigator-directed control group: service as usual (SAU), consisting of standard mental healthcare (treatment as usual) and standard municipal vocational rehabilitation.

We hypothesized that the INT would be superior to both MHC and SAU, and that MHC would be superior to SAU on all the pre-defined outcomes: RTW rates, symptom levels, functioning, presenteeism, self-efficacy, quality of life and client satisfaction.

## Methods

### Study design and participants

This study was a randomized, multisite trial, with recruitment, eligibility assessment and intervention delivery running parallel on each site. Only randomization was centralized, through an online service, accessible to trial staff on all four sites. The study was registered on ClinicalTrials.gov with the registration number NCT02885519 on 31 August 2016 and was last updated on 25 January 2021. The study design has previously been published (11). Those eligible were at least 18 years of age and had been receiving sick-leave benefits for at least 4 weeks due to (i) stress, as defined by the Four-Dimensional Symptom Questionnaire (4DSQ) distress-subscale, (ii) adjustment disorder according to ICD-10, or (iii) exhaustion disorder according to the definition from the Swedish National Board of Health and Welfare. While of these, only adjustment disorders are listed in ICD-10, the other diagnoses are commonly used as diagnostic labels in Denmark, why we chose to acknowledge and operationalize these in our study. The diagnosis was evaluated by clinical research staff guided by the Mini Neuropsychiatric Interview (12). RCT eligibility was determined in conjunction with the clinical assessment. We excluded those who were at moderate or higher risk of suicide, had substance abuse disorder, were pregnant, showed signs of dementia, had an unstable medical condition, or were unwilling to abstain from seeking supplementary mental healthcare outside that provided through this RCT. Participants could be randomized regardless of employment status, being either on full- or part-time sick leave from either employment or unemployment.

In Denmark, all public employment services are delivered through municipal job centers. In these centers, any sickness absentee who wish to have lost salary reimbursed through sick leave benefit is assigned a case manager who manages the benefit case. This management is regulated via national legislation, stipulation among other that time to RTW should be as low as reasonably possible and that the case manager should conduct mandatory sickness benefit criteria controls at least at certain points in time. During the trial period, case managers could refer absentees for trial eligibility assessment if they suspected a mental health issue as the main cause of sick leave. Before assessment, participants completed an online self-report questionnaire addressing a variety of domains related to health status, functioning and self-efficacy, and life-quality. Another RCT with a very similar design for persons with anxiety and depression was conducted simultaneously, on each site (ClinicalTrials.gov ID: NCT02872051) (13), but all analyses were completely separate between the RCT.

### Randomization and masking

If eligible, and after giving written consent, the participant was subsequently randomized by a non-blinded staff member. The allocation to the intervention groups, INT, MCH or SAU, was random in the ratio 1:1:1. A computer generated the allocation sequence with concealed varying block size, stratified for diagnosis, research site, and employment status. All researchers involved in the analyses were blinded until all analyses at 12 months had been conducted.

### Procedures

Participants were clinically assessed at baseline and were followed up after 6, 12 and 24 months. At baseline and all follow-ups, they completed online questionnaires reflecting the chosen outcomes. At follow-up, we obtained register-based data from the national DREAM database containing information about the benefit received per week and on the monthly salary. We performed fidelity reviews to monitor protocol adherences regarding intervention implementation; we developed a fidelity scale for this purpose. To monitor the participants’ use of interventions within the study, and their use of the same type of services outside the study, post-hoc we counted the total use of the services in the three groups. Intervention delivery methods and results and fidelity reviews are presented in detail in supplementary material (www.sjweh.fi/article/4021), supplement 3, including a description of the intervention duration, session length and number of sessions. Additionally, the integration of services in the INT were investigated in an in-depth qualitative process evaluation (14).

The IBBIS interventions (delivered in the INT and MHC groups, respectively) were designed with the intention of simultaneously hastening RTW and improving health. They were delivered in the INT (integrated) group and the MHC (not integrated) group, while participants allocated to the SAU group received both standard mental healthcare and standard vocational rehabilitation.

Participants allocated to SAU, received mental healthcare delivered by or via their GP, private psychologist or psychiatrist, but no healthcare was provided in the job centers. Job centers in the municipality offered standard vocational rehabilitation that included management of the sickness benefit case, as well as occasional assessment of workability, and miscellaneous short-term programs with instruction and support for job searching. Job centers also offered unpaid internships and graded RTW depending on initial employment status.

Participants randomized to MHC received IBBIS mental healthcare, a manualized stepped care programme. It was informed by a literature review of best practice RTW interventions targeting the relevant population. It included stress-coaching and MBSR, inspired by the work by Netterstrøm et al (15). Treatment was delivered by care managers, who were health professionals with relevant training and at least one year of experience in mental health services. Any vocational rehabilitation in the MHC group was delivered through the job centers, as in the SAU group.

Participants randomized to INT received IBBIS mental healthcare as in the MHC group and IBBIS vocational rehabilitation. The latter intervention was inspired by existing evidence-based vocational interventions such as Individual Placement and Support (16), problem solving therapy, and SHARP-at-work (17). It focused on rapid, stepwise RTW and prevention of sick-leave relapse. The IBBIS Vocational Rehabilitation intervention was delivered by employment specialists, who were specifically trained for this study. In INT, IBBIS mental healthcare and IBBIS vocational rehabilitation was integrated through a range of integration activities. These were (i)) roundtable meetings with the participant, care manager and the employment specialist, (ii) co-location of all care managers and employment specialists, and (iii) multidisciplinary, joint supervision.

### Outcomes

The primary outcome was time from baseline to stable RTW measured at the 12-month follow-up. We chose this outcome because we expected most people to return to work within a year, and because sick-leave duration is associated with risk of permanent exclusion from the labor market. Baseline was defined as the day of randomization, and the event ‘stable RTW’ was defined as beginning four consecutive weeks of salaried work with no concurrent vocational benefits. Secondary vocational outcomes were time to stable RTW measured at 6 months, and ‘proportion in ordinary work’ at 12 months. Other secondary outcomes were the following four scales, all measured through four self-report questionnaires at the 6-month follow-up: level of depression using the Beck Depression Inventory (BDI) (18), level of anxiety using the Beck Anxiety Inventory (BAI) (19), level of stress using Cohen’s Perceived Stress Scale (PSS) (20), and functioning using the Social and Work-related Function Scale (WSAS) (21). Pre-planned exploratory outcomes were total numbers of weeks at work at 12 months, at and at 6, 12 and 24 months the following self-reported outcomes: 4 Dimensional Symptoms Questionnaire (4DSQ) (22), exhaustion (23), illness perception (24), quality of life (25, 26), self-efficacy (27, 28), presenteeism (29), and intervention satisfaction (30). All 24-months outcomes will be presented elsewhere, as well as health economic evaluation. Post-hoc, we produced curves displaying proportion in work over time to measure harm as frequency of suicide and self-harm during the 12 months after baseline.

### Statistical analysis

We calculated the sample size on the basis of the primary outcome: median number of days in the control group we conservatively estimated to be 210 days, observed in a similar study group (31). We estimated that a sufficient and clinically relevant hazard ratio (HR)would be 1.5. Due to the three-armed design of the trial, we Bonferroni corrected the acceptable type I error risk from 0.05 to 0.0167, and hence we report 98.3% confidence intervals. The acceptable type II error risk was set to 10%. In total, 201 participants were necessary in each of the three arms.

For time to RTW outcomes we used Cox regression to estimate hazard ratios. For proportion in work at 12 months we estimated odds ratios using logistic regression. All secondary self-report outcomes were analyzed using linear mixed-effects models with unstructured covariance. All analyses of primary and secondary outcomes were based on intention-to-treat principle. Missing data regarding self-report questionnaires were handled by generation of 100 multiple imputations by chained equations (MICE) using stratification variables, primary outcome data, and the four self-reported secondary outcome measures as predictor variables. No missing register data were expected, and only complete case analyses were planned. We pre-planned subgroup analyses per diagnosis, per IBBIS team, per employment status group, and per first and last half of the randomized individuals. Additional analyses were conducted adjusted for the interaction between diagnosis and intervention. We performed sensitivity analyses in a worst-/best-case scenario manner: missing cell values were replaced with group mean ± 2 standard deviations for self-report outcomes, and min/max values for register-based outcomes. We decided, post-hoc, to perform all vocational outcome analyses adjusted for the interaction of intervention groups with (i) employment status, (ii) first versus last half, and (iii) IBBIS team allocation, respectively. Minor changes to the trial were made after trial commencement, and all protocol deviations are exhaustively reported in supplement 1. A comprehensive description of the methodology is found in supplement 2.

## Results

**Figure 1:**
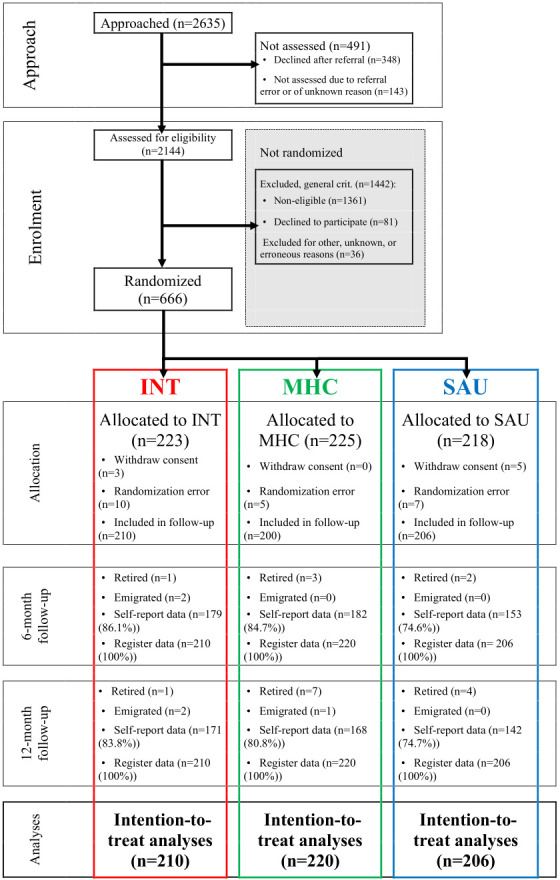
Participant flowchart; MHC: Mental healthcare; SAU: Service as usual; INT: Integrated intervention.

A total of 2209 sickness absentees underwent mental health assessment. Recruitment was simultaneous for this and a similar RCT regarding anxiety and depression. However, the latter RCT did not achieve the necessary 603 participants within the scheduled period and inclusion period was prolonged for both studies. It ended when both trials had reached sample size. Followingly, 666 participants were randomized in this RCT between 2 May 2016 and 30 April 2018. Of these participants, 22 were subsequently excluded, as we after randomization discovered they had not met the inclusion criteria and therefore were randomized by mistake. Another 8 participants withdraw consent and were excluded. Hence, participants included for analyses in the three groups were as follows: SAU: 210, MHC: 220, and INT: 206. Figure 1 illustrates the participant flow.

### Baseline data

Of the participants, 77% were female and mean age was 45 (standard deviation 10) years. Most participants were diagnosed with exhaustion disorder (N=335, 53%), followed by stress (N=184, 29%) and adjustment disorder (N=118, 18%). Baseline characteristics are presented in table 1.

**Table 1:** Baseline characteristics. [SD=standard deviation; MHC=mental healthcare; SAU=treatment as usual; INT=integrated intervention; BDI=Bech Depression Inventory; BAI=Bech Anxiety Inventory; PSS=Perceived Stress Scale; CPH=Copenhagen; GENT=Gentofte; GLAD=Gladsaxe; LTK= Lyngby-Taarbæk; WSAS=Work and Social Adjustment Scale; VE=vocational education

Level	Missing %	Groups					

		INT (N=210)		MHC (N=220)		SAU (N=206)	
		
		Mean (SD)	N (%)	Mean (SD)	N (%)	Mean (SD)	N (%)
Age, years	0.0	45.22 (9.42)		45.26 (10.08)		43.18 (10.23)	
Gender							
Female	0.0		155 (73.8)		163 (74.1)		169 (82.0)
Male			55 (26.2)		57 (25.9)		37 (18.0)
Educational level (highest achieved)							
Primary school	0.0		18 (8.6)		16 (7.3)		15 (7.3)
Secondary/VE			61 (29)		68 (30.9)		62 (30.1)
Bachelors and above			131 (62.4)		136 (61.8)		129 (62.6)
Municipality							
CPH	0.0		127 (60.5)		133 (60.5)		121 (58.7)
GENT			26 (12.4)		27 (12.3)		28 (13.6)
GLAD			29 (13.8)		30 (13.6)		28 (13.6)
LTK			28 (13.3)		30 (13.6)		29 (14.1)
Vocational status							
Employed	0.0		177 (84.3)		188 (85.5)		177 (85.9)
Unemployed			33 (15.7)		32 (14.5)		29 (14.1)
BDI	0.3	21.15 (8.45)		20.45 (8.88)		20.35 (8.23)	
BAI	0.3	15.70 (8.76)		14.43 (7.56)		15.75 (7.49)	
PSS	0.3	23.32 (5.48)		22.67 (5.52)		23.13 (5.53)	
WSAS	2.2	21.46 (7.92)		20.97 (7.93)		22.05 (7.92)	
Sick leave, weeks	0.0	10.62 (2.80)		11.06 (3.99)		11.34 (3.66)	
Diagnosis							
Adjustment disorder	0.0		41 (19.5)		37 (16.8)		39 (18.9)
Exhaustion disorder			109 (51.9)		118 (53.6)		108 (52.4)
Stress			60 (28.6)		65 (29.5)		59 (28.6)

### Implementation and delivery of interventions

Participants in the INT group, compared with those in the MHC group, received more sessions (mean 7.3 versus 5.6) and had longer treatment courses (mean 142 versus 122 days). Most of the participants in the SAU group (81.5%) received at least some healthcare treatment outside IBBIS during the trial. In total, 51.2% of the MHC group and 42.3% of the INT group reported receiving treatment outside IBBIS during the 6-month follow-up, mostly provided by psychologists. The IBBIS mental healthcare was implemented with good fidelity, whereas the IBBIS vocational rehabilitation and integration of services were implemented with fair fidelity (see supplement 3).

### Vocational outcomes

**Figure 2:**
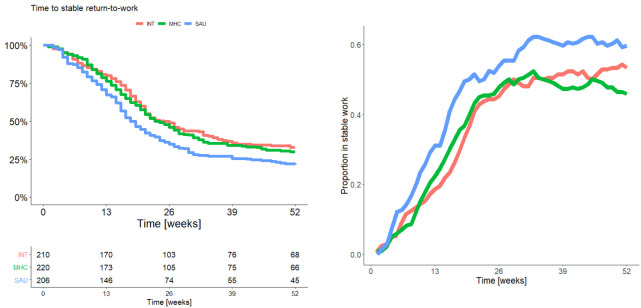
Vocational outcome graphs. Left: Kaplan-Meier curve time to stable RTW. Right: Proportion in stable work per week.

No differences were detected between INT and MHC on the primary outcome time to RTW at 12 months, but SAU was superior to both INT (HR 1.43, P=0.002) and MHC (HR 1.35, P=0.008). Figure 2 displays a Kaplan-Meier curve depicting RTW (left side). SAU was also superior to MHC on the three other vocational outcomes, time to RTW at 6 months (HR 1.41, P=0.0069), weeks in work [risk ratio (RR) 1.24, P=0.003] and proportion in ordinary work at 12 months [odds ratio (OR) 1.78, P=0.005], see the right graph in Figure 2 for proportion in work per week. While MHC and INT showed no difference on any other vocational outcome, SAU was also superior to INT on two of the three other vocational outcomes, time to RTW 6 months (HR 1.51, P=0.001) and weeks in work (RR 1.22, P=0.007) but not proportion in ordinary work at 12 months (OR 1.26, P=0.27). Estimates of differences on the vocational outcomes are presented in table 2.

**Table 2:** Vocational outcomes: group values and pairwise comparison. [IQR=interquartile range; MHC=mental healthcare; SAU=service as usual; INT=integrated intervention; HR=hazard ratio; OR=odds ratio; RR=rate ratio.]

	Group values			Group comparisons					
	
	INT	MHC	SAU	SAU - MHC		SAU - INT		MHC - INT	
		
				Ratio	P-value (98.3% CI)	Ratio	P-value (98.3% CI)	Ratio	P-value (98.3% CI)
Time to RTW (6 months)	25 weeks	23 weeks	19 weeks	1.41 ^HR^	0.0069 ^a^ (1.04–1.91)	1.51 ^HR^	0.0015 ^a^ (1.1–2.06)	1.07 ^HR^	0.590 (0.78–1.48)
Time to RTW (12 months)	IQR 16–>52	IQR 14–>52	IQR 11–43	1.35 ^HR^	0.0077 ^a^ (1.03–1.78)	1.43 ^HR^	0.002 ^a^ (1.08–1.89)	1.06 ^HR^	0.60 (0.8–1.41)
Proportion in work (12 months)	53.8%	46.5%	60%	1.76 ^OR^	0.0053 ^a^ (1.08–2.85)	1.26 ^OR^	0.271 (0.76–2.08)	0.71 ^OR^	0.099 (0.44–1.17)
Mean (SD) weeks of work (12 months)	19.4 (SD 5.4)	19.3 (SD 5.2)	24 (SD 6.4)	1.24 ^RR^	0.003 ^a^ (1.04–1.47)	1.22 ^RR^	0.007 ^a^ (1.02–1.45)	0.99 ^RR^	0.868 (0.82–1.19)

a P-value ≤0.0167.

### Self-report data outcomes

At the 6-month follow-up, SAU yielded higher exhaustion level than INT (KES difference: 3.04, P=0.044) but not regarding other outcomes. Comparing MHC with INT on symptom measures, only one difference was found: MHC yielded lower perceived stress scores than did INT at 6 months (PSS difference: -1.4, P=0.026). Several differences on symptom scales were seen between the SAU and MHC groups with the SAU group showing more symptom scores regarding anxiety (difference on BAI: 2.16, P=0.005), perceived stress (difference on PSS: 1.83, P=0.006), exhaustion (difference on KES: 5.2, P<0.005), somatization (difference on 4DSQ-somatization: 1.28, P=0.040) and psychological distress (difference on 4DSQ-distress: 1.41, P=0.040). On the WSAS functioning scale, a higher score was observed at 6 months in the SAU group compared with the MHC group (difference: 2.11, P=0.023). On client satisfaction measured only at 6 months, participants allocated to SAU reported lower client satisfaction than those allocated to MHC (difference: -2.68, P<0.005) and INT (difference: -2.86, P<0.005). No differences were found between the MHC and INT groups.

At the 12-month follow-up, the only difference observed across all outcomes and group comparisons was symptoms of exhaustion, lower in the MHC group, compared to SAU (difference on KES: 3.49, P=0.029). On all self-efficacy outcomes, life quality outcomes, and presenteeism, no differences were observed between groups at either 6 or 12 months. See supplements 4 and 5 for results of the subgroup and sensitivity analyses. We found no significant interactions between the stratification variables and interventions on vocational outcomes. Results from analyses on all secondary and exploratory self-report outcomes at 6 and 12 months are shown in table 3.

**Table 3:** Self-report data outcomes. Group means are values after imputation. [Diff.=difference (estimated marginal mean); SD=standard deviation; MHC=mental healthcare; SAU=service as usual; INT=integrated intervention; BDI=Bech Depression Inventory; BAI=; PSS=Perceived Stress Scale; WSAS=Work and Social Adjustment Scale; 4DSQ=Four Dimensional Questionnaire; KEDS=Karolinska Exhaustion Disorder Scale; IPQ=Illness Perception Questionnaire; EQ5DL=health-related quality of life; QoLs=Quality of Life Scale; RTW-SE=return to work-self efficacy; SPS=Stepford Presenteeism Scale; GSE=Generalized Self-Efficacy Scale; CSQ=The Client Satisfaction Questionnaire]

Outcome domain	Outcome Secondary (S) Exploratory (E)	Group comparisons					

		SAU - MHC		SAU - INT		MHC - INT	
		
		Diff	P-value (98.3%CI)	Diff	P-value (98.3%CI)	Diff	P-value (98.3%CI)
**6-month follow-up**							
Symptoms	BAI (S)	2.16	0.0051 ^a^ (0.32–4.01)	1.23	0.122 (-0.67–3.13)	-0.97	0.199 (-2.79–0.84)
	BDI (S)	1.37	0.112 (-0.7–3.44)	0.52	0.548 (-1.55–2.59)	-0.84	0.316 (-2.85–1.17)
	PSS (S)	1.83	0.0055 ^a^ (0.26–3.41)	0.43	0.506 (-1.13–2)	-1.40	0.0261 ^b^(-2.91–0.11)
	KES (E)	5.24	0.0007 ^a^ (1.57–8.91)	3.04	0.0445 ^b^(-0.59–6.67)	-2.20	0.133 (-5.7–1.31)
	4DSQ-somatization (E)	1.28	0.0402 ^b^ (-0.21–2.77)	0.97	0.136 (-0.59–2.52)	-0.36	0.544 (-1.76–1.05)
	4DSQ-distress (E)	1.41	0.0365 ^b^ (-0.21–3.03)	1.06	0.114 (-0.55–2.68)	-0.33	0.604 (-1.88–1.21)
	4DSQ-anxiety (E)	0.70	0.065 (-0.21–1.6)	0.55	0.14 (-0.34–1.43)	-0.17	0.64 (-1.03–0.7)
	4DSQ-depression (E)	0.14	0.527 (-0.38–0.66)	-0.06	0.788 (-0.6–0.48)	-0.19	0.4 (-0.73–0.35)
Functioning	WSAS (S)	2.11	0.0234 ^b^ (-0.12–4.33)	0.99	0.28 (-1.2–3.18)	-1.13	0.195 (-3.22–0.96)
Presenteeism	SPS (E)	0.13	0.675 (-0.62–0.89)	0.12	0.682 (-0.6–0.85)	0.00	0.99 (-0.67–0.66)
Self-efficacy	IPQ (E)	-0.09	0.801 (-1–0.81)	-0.31	0.409 (-1.21–0.59)	-0.20	0.585 (-1.07–0.67)
	GSE (E)	-0.30	0.632 (-1.82–1.22)	0.53	0.411 (-1.01–2.07)	0.83	0.187 (-0.68–2.35)
	RTW-SE (E)	-0.47	0.588 (-2.53–1.6)	-0.20	0.811 (-2.25–1.84)	0.30	0.713 (-1.67–2.28)
Life quality	QoL (E)	-0.10	0.94 (-3.24–3.04)	0.53	0.682 (-2.6–3.67)	0.60	0.636 (-2.45–3.65)
	EQ5 (E)	-0.02	0.166 (-0.05–0.01)	0.00	0.787 (-0.03–0.03)	0.01	0.246 (-0.02–0.04)
Satisfaction	CSQ (E)	-2.68	<0.0005 ^a^ (-4.12–-1.24)	-2.86	<0.0005 ^a^(-4.29–-1.43)	-0.18	0.744 (-1.49–1.14)
**12-month follow-up**							
Symptoms	BAI (E)	1.32	0.084 (-0.51–3.14)	1.03	0.184 (-0.83–2.9)	-0.31	0.676 (-2.09–1.47)
	BDI (E)	-0.20	0.819 (-2.28–1.88)	-0.49	0.563 (-2.53–1.55)	-0.27	0.747 (-2.28–1.74)
	PSS (E)	0.38	0.57 (-1.23–2)	0.50	0.456 (-1.11–2.12)	0.12	0.857 (-1.49–1.73)
	KES (E)	3.56	0.0233 ^b^(-0.2–7.31)	2.28	0.147 (-1.49–6.04)	-1.27	0.412 (-4.98–2.44)
	4DSQ-somatization (E)	0.92	0.139 (-0.57–2.41)	0.89	0.163 (-0.64–2.42)	-0.07	0.911 (-1.48–1.35)
	4DSQ-distress (E)	0.45	0.503 (-1.17–2.07)	0.44	0.507 (-1.16–2.05)	0.01	0.994 (-1.6–1.61)
	4DSQ-anxiety (E)	0.47	0.196 (-0.4–1.33)	0.42	0.233 (-0.43–1.27)	-0.05	0.874 (-0.88–0.77)
	4DSQ-depression (E)	-0.31	0.144 (-0.83–0.2)	-0.18	0.41 (-0.7–0.34)	0.14	0.521 (-0.39–0.68)
Functioning	WSAS (E)	0.05	0.955 (-2.18–2.29)	-0.22	0.812 (-2.45–2.01)	-0.28	0.754 (-2.42–1.86)
Presenteeism	SPS (E)	0.03	0.932 (-0.9–0.97)	-0.32	0.397 (-1.21–0.58)	-0.31	0.375 (-1.15–0.53)
Self-efficacy	IPQ (E)	-0.03	0.936 (-0.96–0.9)	-0.22	0.578 (-1.19–0.74)	-0.18	0.649 (-1.1–0.75)
	GSE (E)	0.08	0.898 (-1.45–1.61)	0.97	0.138 (-0.6–2.54)	0.90	0.161 (-0.64–2.44)
	RTW-SE (E)	-0.16	0.858 (-2.26–1.95)	-0.04	0.963 (-2.14–2.06)	0.16	0.857 (-1.9–2.21)
Life quality	QoL (E)	1.28	0.327 (-1.85–4.41)	0.65	0.616 (-2.45–3.74)	-0.65	0.61 (-3.71–2.41)
	EQ5 (E)	-0.01	0.468 (-0.04–0.02)	0.00	0.993 (-0.03–0.03)	0.01	0.452 (-0.02–0.04)

a P≤0.0167.

b 0.05 ≤P< 0.0167.

### Harms

We observed no suicides, fatalities, or self-harm in any group. Numbers of referrals to hospital-based mental health until 6-month follow-up in the groups were INT 17, MHC 11, and SAU 6.

## Discussion

### Main findings

Contrary to our hypotheses, this study showed that INT was not superior to either SAU or MHC and, further, that MHC was not superior to SAU on the primary outcome RTW rates at 12 months. On the primary outcome and all other vocational outcomes, SAU was superior to MHC, and this was also the case for SAU compared with INT, except on proportion in work at 12 months. INT did not differ from MHC on any vocational outcome. Had the faster RTW in SAU been counterbalanced by a higher rate of current sick-leave after individuals’ initial RTW, we would have seen at least a tendency towards contrasting differences on the weeks-in-work outcome.

Across the three group comparisons, regarding self-reported secondary outcomes, MHC yielded somewhat better results than SAU, yet the differences were of generally low clinical significance. Overall, the hypothesis that the integrated intervention (INT) would be superior to SAU and MHC could not be supported and, similarly, neither could the hypothesis that MHC was superior to SAU. On the contrary, SAU was superior to INT due to the beneficial vocational differences and lack of symptoms differences. The vocational superiority of SAU compared with MHC was somewhat counterbalanced by the differences on self-reported outcomes; hence SAU cannot unequivocally be perceived as superior to MHC.

### Other studies

An RCT by Bakker et al (32) regarding persons with self-reported stress found that the median number of sick-leave days was 96 in the intervention group and 102 in the control group. In our study, median number of days before RTW was 133 (SAU), 161 (MHC) and 175 (INT). In a study by Blank et al (33) regarding those on sick leave with psychological complaints, median sick-leave days for a combined intervention group were 122, for a CBT group 329, and 320 for a control group (33). On the vocational outcome *proportion in work at 12 months*, in our study, 52.5% had returned to work in the SAU group, 40.6% in the MHC group, and 52.2% in INT group. In a smaller study for persons with burnout, two rehabilitation programme groups both had ~60% still on sick leave after 12 months – a result similar to that of our MHC group (34). The Norwegian AWaC study, which found a positive vocational effect of their integrated intervention, had 44.2% in work participation at 12 months in the intervention group, and 37.2% in the control group (9). However, the population in that study consisted of persons with anxiety, depression, and subthreshold conditions and only 47.5% of the study population was similar to the population of this study (on full- or part-time sick leave at baseline, either employed or not). Overall, this suggests that other studies have shown more positive outcomes on median number of days to RTW, while participants in the SAU group had a higher proportion in work at follow-up compared with the populations in these studies.

### Interpretation of findings

The reason why both IBBIS groups – the MHC and INT groups – yielded poorer vocational outcomes than the SAU group might be explained by the way the two groups differed from the SAU group, namely by the IBBIS mental healthcare treatment. We found no significant difference in the quantity of healthcare treatment delivered in the interventions. Therefore, any vocational difference cannot satisfactorily be explained by a quantitative difference; instead, it suggests a qualitative one. While we do not have detailed knowledge of what healthcare services participants in SAU received, some major differences were that the care managers delivering the treatment in IBBIS were organizationally employed in and had received training through hospital-based psychiatry. In Denmark, this part of the healthcare sector per definition targets persons with severe mental disorders as well as those with common mental disorders, but in the latter case only if they have markedly impaired functioning, or moderately impaired functioning on several domains. Being part of the latter population was an exclusion criterion in this study, and hence the care managers were used to and trained in providing treatment for a population with substantially lower functional levels. Accordingly, one hypothesis could be that healthcare professionals had lower than justified expectations of the participants and that such expectations negatively predict outcome (35), which could explain part of the negative effect we observed. Similarly, hospital-based mental healthcare in general shows some latency regarding incorporating vocational goals into its practice (2), and another hypothesis could be that this explains the differences in vocational outcomes compared with SAU. In INT, a process evaluation study showed that the integration between care managers and employment consultations did not unfold as protocolled (14). Hence, part of the negative vocational outcomes in the INT group, compared with the SAU group, might have been different had integration been carried out as protocolled. Nevertheless, integration was not utilized in the MHC group; therefore, lack of integration cannot alone account for the negative outcomes of both groups. However, the care managers delivering healthcare were the same group of practitioners in both the MHC and the INT groups. While taking part in the relational coordination in INT, care managers might have gain knowledge about the sick-leave benefit case management conducted by the employment consultants. This knowledge may have enabled them to influence the case managers of participants in both IBBIS groups to ease sick-leave benefit criteria control, indicated by the process evaluation (14). This claim is supported by the studies demonstrating the association between rigidity of such control and hastened RTW (36–38).

Several of the many self-reported outcomes were better in the MHC group than in the SAU group, but never vice versa. Differences were of low effect size and for scales with an established minimally relevant clinical difference, differences were below that value. However, on most of these outcomes all groups had almost reached clinical remission at the 6-month follow-up, perhaps largely due to regression towards the mean. One reason for the better self-reported outcomes in the MHC group could be that this group was the only group where all participants received systematically delivered mental healthcare, which was not the case in the SAU group. Furthermore, in the MHC group the relation between the individual participants and their care manager did not imply detailed sharing of personal information with the sickness benefit case manager, as was the case in the INT group, due to the integration. This sharing of information with INT might have impaired the effect of the mental healthcare, leading to a lower effect as it has long been recognized that privacy is pivotal in such treatment (39).

The only exception regarding effect-size magnitude was intervention satisfaction, where both the MHC and the INT group showed much higher levels of satisfaction than did the SAU group, indicating that participants in general were highly dissatisfied with the interventions usually provided. Part of the positive effect on symptom levels may be due to a reporting bias as a consequence of treatment expectation because participants were not blinded to group allocation, and expectation has been proven as a significant predictor of similar outcomes (40).

### Harms

We found no evidence of differences in harm attributable to any of the three interventions. The apparent difference in number of referrals to hospital-based mental healthcare in the INT and MHC groups compared with the SAU group is seemingly due to a greater knowledge of referral criteria or referral possibility amongst mental healthcare staff in the INT and MHC interventions, who were formally employed in the organization receiving these referrals, rather than due to harm attributable to the interventions.

### Strengths and limitations

A large pre-planned, sufficient sample size was achieved, randomization was successful and few data were missing on vocational outcomes. However, on self-report outcomes, degree of missingness differed between groups. Best/worst case sensitivity analyses showed vocational outcomes to be very robust and, on self-reported outcomes, reasonably robust. Vocational outcomes were calculated from actual salary and sick-leave benefit payments, and hence free from reporting bias. Participants were not blinded, and self-reported outcomes may be biased by expectations. RTW processes are highly dependent on legislative context, and external validity across time and borders might be affected. SAU entailed largely different outcome across time periods of the trial, also affliction the external validity of the comparative effects of the trialed interventions. Fidelity reviews and intervention delivery indicate moderate deviations from that protocolled.

### Implications

This study indicates that neither the mental healthcare intervention, as in the MHC group, nor integrated intervention, as in the INT group, improves the RTW process for people on sick leave with stress-related disorders. On the contrary, both interventions significantly worsened vocational outcomes, without any substantial counterbalancing of clinically relevant positive impacts on health or functioning. Hence, none of the IBBIS interventions, as implemented in this trial, can be recommended as an alternative to service as usual. Replication is needed to test the true efficacy of the IBBIS interventions.

### Concluding remarks

This study tested whether INT was superior to MHC and SAU on vocational outcomes, symptoms and functioning in a population of persons on sick leave due to a stress-related disorder. Neither of the tested interventions – INT and MHC – as they were implemented in this trial, can be recommended as a means of hastening RTW or increasing work participation; in fact, the contrary is manifestly the case. However, even though MHC hampers RTW, it alleviates some symptom domains more than SAU does, albeit only slightly. Therefore, SAU cannot unequivocally be recommended over MHC.

## Supplementary material

Supplements 1-5

## Data sharing

By contacting the corresponding author, data can be shared, including individual participant data along with a data dictionary. Data can include all data used for calculating all outcomes in the study, and all relevant data for assessing eligibility for referred participants. Such data sharing requires an application stating the detailed purpose of data retrieval. A data sharing agreement is required, and this must fulfil the requirements of the EU General Data Protection Regulation 2016/679 (GDPR).
