# Supplementary material for: Integrating vocational rehabilitation and mental healthcare to improve the return-to-work process for people on sick leave with stress-related disorders: results from a randomized trial
Source: Scand J Work Environ Health. 2022 Jun 30;48(5):361–71. doi: 10.5271/sjweh.4021 (PMC9527782; doi:10.5271/sjweh.4021)
Supplement: Supplements 1-5 [file SJWEH-48-361-S001.pdf]

Integrating vocational rehabilitation and mental healthcare to improve the return-to-work process for people on sick leave with stress-related disorders: results from a randomized trial[1]

by Andreas Hoff,[2] Jonas Fisker, Rie Mandrup Poulsen, Carsten Hjorthøj, Nicole Kristjansen Rosenberg, Merete Nordentoft, Anders Bo Bojesen, Lene Falgaard Eplov

1. Supplementary material
2. Correspondence to: Andreas Hoff, Copenhagen Research Institute for Mental Health [CORE], Mental Health Services Capital Region of Denmark, Copenhagen. [E-mail: andreas.hoff@regionh.dk]

Supplement 1: Deviations from protocol

Regarding interventions

- For several months, during the first temporal half of the RCT, the general practitioners (GP) did not receive a conclusion note from the mental health assessment. This procedure was implemented with the intention to improve GP treatment, in all three intervention groups.
- Roundtable meetings in the INT group meetings were conducted later in the care manager course than planned.
- In general, the average of cognitive behavioural therapy session numbers was lower than planned per protocol.

Regarding statistical analyses

- We draw conclusions prior to unblinding, but thereafter we discovered an error in calculation of the weekly vocational status. Followingly, all analyses were performed again, and conclusions drawn once again. The erroneous results and conclusions drawn hereupon, can be obtained from corresponding author.
- We did not describe any censoring principles regarding the outcomes “proportion in work” and chose to apply the principles described regarding the primary outcome.
- Before publication of statistical analysis plan, *and before unblinding*, complete case-analyses of the four secondary outcome measures were performed as preliminary, blinded results (using ANOVA to calculate estimated marginal mean differences between groups). These results were discarded, since it did not adhere to the original plan of using linear mixed effects model at multiple imputed datasets.
- We discovered a discrepancy between statistical analysis plan and the published study design article, describing the protocol: both described that salary was a criteria for return to work, but the study design article described return to work as a period “with no concurrent vocational benefits”, whereas the statistical analysis plan said “with no sick leave benefit”, the latter only implying that any other vocational benefits than sick leave benefits along with salary could constitute return to work. We chose the definition from the study design article.
- In the statistical analysis plan we planned statistical assumption control of proportional hazards in cox-regression, and that we would alternatively adjust for different kinds of interactions between time and group assignment. Only if the primary analysis would point to a difference, and hence null-hypothesis rejection, this applied – not in cases were no difference was seen.
- The statistical analysis plan did not mention Kaplan-Meier curves, but study design article did – we obeyed the latter.
- We discarded the outcome *time from the first day of return to work until recurrent sick leave*, since we realized that this time would not consistently reflect a positive outcome. Post-hoc we decided that a proportion per time-curve, as shown in Figure 2, right graph, could benefit exploring patterns of this outcome.
- Post-hoc, we performed all analyses of vocational outcomes adjusted for the interaction of intervention with a) IBBIS team allocation, b) first vs. last half, and c) employment status, respectively.

Supplement 2: Methodological details

Assessment of eligibility was performed by research staff, all clinically trained mental health professionals, supervised by a psychiatric specialist, and specifically trained for the purpose. The assessment was conducted through clinical interview, partly guided by 1) MINI International Neuropsychiatric Interview,[1] 2) Standardized Assessment of Personality - Abbreviated Scale (SAPAS),[2] 3) Attention deficit hyperactivity disorder symptom checklist for adults (Adult Self-Report Scale, ASRS v1·1)[3] and 4) Mini-Mental State Examination (MMSE)[4] (when dementia was clinically suspected).

Eligible were ≥18-year-old sick leave benefit recipients for ≥ four weeks with either 1) stress, as defined by the Four-Dimensional Symptom Questionnaire (4DSQ) distress-subscale, 2) adjustment disorder according to ICD-10, or 3) exhaustion disorder according to the definition from the Swedish National Board of Health and Welfare. Participants were required to have Danish proficiency and give written consent. Excluded were those who were pregnant, in at least moderate risk of suicide according to clinical assessment and stratification according to the MINI International Neuropsychiatric Interview,[1] had clinically significant substance abuse disorder according to clinical assessment by assessor, other unstable medical condition judged by assessor to yield significant obstacles for mental health care treatment in the research project, showed signs of dementia by assessors' clinical judgement, or could not indicate willingness to abstain from seeking mental health care outside the study, whilst receiving such through the study.

Statistical analyses

A detailed statistical analysis plan, following the consensus template for such, recommended by a wide range of relevant bodies,[5] was submitted for publication on www.clinicaltrials.org on July 5th, 2019, before any analyses of primary outcome data was scrutinized. It was followed notoriously (with few deviations, see supplements) regarding, for each outcome, its calculation, analysis method, results presentation, covariate adjustment, statistical method assumption control (and assumption fail-alternatives), sensitivity analyses and handling of missing data. All analyses were performed using base functions in *R*,[6] and packages *survival*, *mice*, *lme4*, and *sandwich*. All analyses adhered to the intention-to-treat-principle (except the outcome *employee productivity*):

- For all time to return to work outcomes we performed cox-regression to calculate hazard rate ratios.
- For proportion in work outcomes we calculated odds ratios using logistic regression.

- For weeks in work outcomes we calculated rate ratios using Poisson regression and a Sandwich-Hubert estimator to correct standard errors due to expected severely skewed data.
- For self-reported (all numerical) outcomes we calculated pairwise group differences of estimated marginal means, using linear mixed-effects model, with unstructured covariance. Exceptions were outcomes without baseline scores–*client satisfaction* and *employee productivity*–where we used generalized linear models.
- Adjustments were, in all analyses, made for only randomization stratification variables, as recommended for RCTs by European Medicines Agency.[7]
- Missing data regarding self-report questionnaires was handled by generation of 100 multiple imputations by Chained Equations (MICE), using stratification variables, primary outcome data, and the four self-reported secondary outcome measures as predictor variables. No missing register data was expected, and only complete case analyses were planned.
- Subgroup analyses were performed for all outcomes in following strata, all pre-planned: per diagnosis (anxiety; depression); per employment at baseline (employed; unemployed); per two IBBIS teams (Team City (Copenhagen municipality) and Team North (remaining municipalities); per first and last temporal half. Furthermore, all analyses were performed adjusted for the interaction of diagnoses and intervention.
- Sensitivity analyses were, for register based data-outcomes, performed by including the missing data (due to consent withdrawal or register failure), with all their outcomes handled as either the worst possible (no return to work at follow-up) vs. best possible (return to work at first observation). For self-reported outcomes all missing data was single imputed as the mean of the outcome variable ±2 standard deviations in the best/worst case scenario manner.

Predefined exploratory outcomes:

- ➢ Register-based:
  - → *Total numbers of weeks at work* (12-month follow-up), with one week at work being without sick leave benefit and any salary from an ordinary job during that week
- ➢ Self-reported at 6- and 12-month follow-up:
  - → *Levels of depression, anxiety, somatization and distress*, by 4 Dimensional Symptoms Questionnaire, 4DSQ,[8]
  - → *Levels of exhaustion*, measured with Karolinska exhaustion disorder scale, KEDS,[9]
  - → *Illness perception*, by Illness Perception Questionnaire, IPQ,[10]

- → *Health related quality of life*, by EQ5DL,[11]
- → *Quality of life*, measured by *Quality of Life Scale*, QoL,[12]
- → *Return to work-self efficacy*, by RTW-SE,[13]
- → *Generalised self-efficacy* measured with *Generalized Self-Efficacy Scale*, GSE,[14]
- → *Employee productivity*, measured with the Stepford Presenteeism scale, SPS,[15] (NB: this outcome was measured only among participants in any vocational activity (supported or competitive), and is hence not an *intention-to-treat* analysis)

➢ Self-reported at 6-month follow-up:

- → *Client satisfaction*, by *The Client Satisfaction Questionnaire,*[16]

All outcomes at 24-month follow-up will be reported later.

Changes to predefined outcomes

Before any analysis of any vocational outcomes, and before 24-month follow-up, we discarded the recurrent sick leave-outcome due to failure to find reasonable statistical measures.

Intervention delivery description

Pre-planned:

- → Self-reported at 6-month follow-up: Any use of psychotherapy-like interventions, regardless of funding source, at general practitioners, psychiatrists, psychologists, coaches, psychotherapists or group therapy.

Post-hoc we decided to describe the following parameters:

➢ Delivered outside the study interventions:

- → Number of publicly funded consultations at general practitioners, psychiatrists and psychologist
- → Use of psychiatric admissions, out-patient consultations and emergency room
- → Use of vocational rehabilitation services: number courses and their duration.

➢ Delivered within the study interventions:

- → Number of contacts and duration of treatment course with health care staff
- → Number of roundtable-meetings, and their relative placement in the treatment

➢ Delivery across intervention placement:

- → Employment consultant consultations, meetings and virtual contacts

References

1 Sheehan D V., Lecrubier Y, Sheehan KH, *et al.* The Mini-International Neuropsychiatric Interview (M.I.N.I.): The development and validation of a structured diagnostic psychiatric interview for DSM-IV and ICD-10. In: Journal of Clinical Psychiatry. 1998: 22–33.

2 Moran P, Leese M, Lee T, Walters P, Thornicroft G, Mann A. Standardised Assessment of Personality - Abbreviated Scale (SAPAS): Preliminary validation of a brief screen for personality disorder. *Br J Psychiatry* 2003; **183**: 228–32.

3 Kessler RC, Adler L, Ames M, *et al.* The World Health Organization Adult ADHD Self-Report Scale (ASRS): a short screening scale for use in the general population. *Psychol Med* 2005; **35**: 245–56.

4 Cole MG, McCusker J, Elie M, Dendukuri N, Latimer E, Belzile E. Systematic detection and multidisciplinary care of depression in older medical inpatients: a randomized trial. *Can Med Assoc J* 2006; **174**: 38–44.

5 Gamble C, Krishan A, Stocken D, *et al.* Guidelines for the Content of Statistical Analysis Plans in Clinical Trials. *JAMA* 2017; **318**: 2337.

6 R: A Language and Environment for Statistical Computing. Vienna, Austria. 2019; **0**. https://www.r-project.org/.

7 European Medicines Agency, Committee for Medicinal Products for Human Use (CHMP). European Medicines Agency - Adjustment for baseline covariates in clinical trials. Eur. Med. Agency. 2015.

8 Terluin B, van Marwijk HWJ, Adèr HJ, *et al.* The Four-Dimensional Symptom Questionnaire (4DSQ): a validation study of a multidimensional self-report questionnaire to assess distress, depression, anxiety and somatization. *BMC Psychiatry* 2006; **6**: 34.

9 Saboonchi F, Perski A, Grossi G. Validation of Karolinska Exhaustion Scale: Psychometric properties of a measure of exhaustion syndrome. *Scand J Caring Sci* 2013; **27**: 1010–7.

10 Moss-Morris R, Weinman J, Petrie KJ, Horne R, Cameron LD, Buick D. The revised Illness Perception Questionnaire (IPQ-R). *Psychol Health* 2002; **17**: 1–16.

11 Brazier J, Roberts J, Tsuchiya A, Busschbach J. A comparison of the EQ-5D and SF-6D across seven patient groups. *Health Econ* 2004; **13**: 873–84.

12 Burckhardt CS, Anderson KL. The Quality of Life Scale (QOLS): reliability, validity, and utilization. *Health Qual Life Outcomes* 2003; **1**: 60.

13 Lagerveld SE, Blonk RWB, Brenninkmeijer V, Schaufeli WB. Return to work among employees with mental health problems: Development and validation of a self-efficacy questionnaire. *Work Stress* 2010; **24**: 359–75.

14 Schwarzer R, Jerusalem M. Generalized Self-Efficacy scale. In: Wright S, Johnston M, eds. Measures in health psychology: A user’s portfolio. Causal and control beliefs. Windsor, England, 1995: 35–7.

15 Koopman C, Pelletier KR, Murray JF, *et al.* Stanford presenteeism scale: health status and employee productivity. *J Occup Environ Med* 2002; **44**: 14–20.

16 Attkisson C, Zwick R. The Client Satisfaction Questionnaire: Psychometric properties and correlations with service utilization and psychotherapy outcome. *Eval Program Plann* 1982; **5**: 233–7.

Supplement 3: Intervention delivery and fidelity reviews

Section 1 in this supplement describes interventions delivered to the participants in the **IBBIS stress-related disorders RCT**. Section 2 describes the fidelity reviews carried out across the two IBBIS concurrent RCTs (RCT1 including anxiety and depression and RCT2 including stress-related disorders, see the study protocol for explanation).

1 Description of the delivery of interventions in the study, and delivered externally

The study protocol (published on clinicaltrials.org) and the published study design articles described the intended interventions in the respective trial arms. To describe what was delivered *de facto*, we decided to calculate the specific amounts of interventions. Some measures were pre-registered, but post-hoc we decided on further measures, in order to create a nuanced insight:

Pre-planned:

→ Self-reported at 6-month follow-up: Any use of psychotherapy-like interventions, regardless of funding source, at general practitioners, psychiatrists, psychologists, coaches, psychotherapists or group therapy.

Post-hoc we decided to describe the following parameters:

- ➢ Delivered outside the study interventions:
  - → Number of publicly funded consultations at general practitioners, psychiatrists and psychologist
  - → Use of psychiatric admissions, out-patient consultations and emergency room
  - → Use of vocational rehabilitation services: number courses and their duration.
- ➢ Delivered within the study interventions:
  - → Number of contacts and duration of treatment course with health care staff
  - → Number of roundtable-meetings, and their relative placement in the treatment
- ➢ Delivery across intervention placement:
  - → Employment consultant consultations, meetings and virtual contacts

Results are shown in **Fejl! Henvisningskilde ikke fundet.**: The table present interventions delivered to the participants between baseline and 12-month follow-up. The upper panel displays what was delivered by the IBBIS-teams (mental health care in the INT and MHC groups, vocational rehabilitation in the INT group, and none in the SAU group). The lower panel shows descriptive statistic of self-report and register data of interventions delivered to the participants from other providers than the IBBIS-team, except for employment consultant services, where numbers regarding the INT group represent the contacts from the IBBIS-teams as well as any contacts they might have had outside IBBIS (if e.g., they withdraw consent to continue in the IBBIS RCT, in which case they would maybe continue receiving employment consultation services in the municipal jobcentres, outside the IBBIS programme). Self-reported intervention delivery data included all mental health care interventions regardless of financial source and register data only publicly subsidised treatment, yet, some of it might have been only partly subsidised. The gap between the self-report data and register-based hence reflects interventions from private/non-public service providers.

	Intervention type	Measure		Intervention groups INT	MHC	SAU
Delivered **in** IBBIS	Mental health care interventions	**Sessions quantity**	Mean (SD); Median	7.3 (3.1); 8	5.6 (2.6); 6	
		**Cumulated duration, minutes**	Mean (SD); Median	446.1 (191.1); 460	359.8 (114.4); 385	
		**Duration, days**	Mean (SD); Median	142.1 (112.4); 120	122.2 (109.5); 112.2	
	Vocational rehabilitation	**EC meetings**	Mean (SD); Median	5.4 (3); 4.9		
		**EC digital contacts**	Mean (SD); Median	3.5 (3.9); 3		
	First RTM	**Number in CM course**	Mean (SD); Median	3.5 (1.6); 3		
	RTM quantity	**Number**	Mean (SD); Median	1.1 (0.8); 1		
Delivered **outside** IBBIS	Self-report data: Mental health care	**No treatment**	Proportion [%]	57.7	48.8	18.5
		**Sessions, quantity**	Mean (SD)	5.1 (4.6)	5.3 (4.6)	9.1 (5.7)
		**Psychologist**	Proportion [%]	16.8	24.2	43.9
		**Psychiatrist**	Proportion [%]	1.9	2.3	4.4
		**GP**	Proportion [%]	6.2	7.4	17.1
		**Coach or psychotherapist**	Proportion [%]	11.5	10.2	16.6
		**Group therapy**	Proportion [%]	2.9	6	7.8
		**Other**	Proportion [%]	5.8	6	10.2
	Register data: Mental health care	**Sessions, GP**	Mean (SD); Median	5.35 (4.72); 4	5.84 (4.34); 5	6.18 (5.3); 5
		**Sessions, psychologist**	Mean (SD); Median	0.18 (0.71); 0	0.16 (1.01); 0	0.56 (2.07); 0
		**Sessions, psychiatrist**	Mean (SD); Median	0.08 (0.57); 0	0.08 (0.56); 0	0.23 (1.17); 0
		**≥ 1 out-patient psych. contact**	n (proportion, [%])	17 (8.1)	11 (5)	6 (2.9)
		**≥ 1 psych. admission**	n	n/a (<5)	n/a (<5)	n/a (<5)
		**≥ 1 psych. ER contact**	n	0	0	0
	Register data: Vocational rehabilitation	**EC meetings** (NB: for the INT group: in IBBIS)	Mean (SD); Median	4.93 (2.93); 5	2.37 (2.19); 2	2.25 (2.32); 1.5
		**EC virtual contacts** (NB: for the INT group: in IBBIS)	Mean (SD); Median	1 (1.22); 1	1.73 (1.67); 1	1.78 (1.67); 1.5
		**VR course**	n (proportion [%])	21 (10)	43 (19.5)	77 (37.4)
		**VR course, hours (cumulated)**	Mean (SD); Median	3.14 (21.93); 0	9.14 (30.48); 0	28.48 (70.7); 0
		**VR course duration, days (start-to-end)**	Mean (SD); Median	3.2 (13.72); 0	8.69 (23.11); 0	25.68 (46.41); 0

Table 1: Interventions delivered from Baseline to 6-month follow-up. RTM: Roundtable-meeting; GP: General Practitioner; VR: Vocational Rehabilitation; EC: Employment Consultant; CM: Care Manager; SD: Standard Deviation; MHC: Mental health care; SAU: Service as usual; INT: Integrated intervention; ER: Emergency Room; n/a: not available (too few cases, cannot be reported due to personal data regulations)

1.1 Delivered inside the IBBIS study

Regarding mental health care interventions, participants in the INT group received more sessions (Mean: 7.3, SD: 8) and a longer treatment duration from baseline to last session (Mean: 142 days, SD: 112) than the MHC group (Mean: 5.6 sessions, SD: 6; duration days: 122, SD: 110). Most of the participants in the SAU group (81.5 %) received treatment outside IBBIS during the trial, whereas this was appr. half of the participants in the other groups, IND and MHC.

The INT group participants received several more employment consultant contacts with their IBBIS employment consultant than the other groups where the employment consultant contacts took place in municipalities. The median number of contacts with an employment consultant in the INT group was 8, including 3 digital ones. *Roundtable-meetings* were conducted later in the intervention course than planned, as the first roundtable meetings per participant took place later in the course than planned (median 3rd opposed to planned 2nd care manager session). Furthermore, the number was below the estimated two meetings per participant and 13% never had one. A reason for the latter can be that they withdraw from intervention.

1.2 Delivered outside the IBBIS study

In the SAU group only 18.5 % reported having received no treatment at all, and on average 9.1 sessions (SD 5.7), which is higher than the level delivered in IBBIS to the MHC and INT groups, and 44.4 % of the SAU group participants consulted a psychologist (with figures in the other groups much lower), though, registers showed that only very few of these sessions, on average 0.56 (SD 2.07) were publicly financed, and as self-report data confirmed many other financial sources were utilized (e.g. 20.5% of the SAU group participants payed themselves, and 21.5% received employer financing). Rather few participants were during the study period referred to hospital based psychiatric services, no one visited psychiatric emergency services, and numbers too small to report were they who were admitted to a psychiatric hospital.

In the SAU group, participants received on average 1.78 meetings with their employment consultants, and 37.4% of them was provided a vocational rehabilitation course, yielding 28.5 hours per participant on average in the group. For the MHC group the numbers were 1.7 meetings, and 19.5 % yielding 9.14 hours on average. In the INT group: 4.9 meetings (including meetings in IBBIS), and 10 % given courses lasting 3.14 hours on average. Yet, in all three groups, median amount of VR courses was 0, and hence less than half of the participants in each group received such.

2 Fidelity reviews of implementation degree in the IBBIS trials

2.1 Introduction

Implementation-degree of the active IBBIS intervention was investigated through fidelity reviews. Fidelity reviews were done for two reasons. Firstly, implementation was measured to ensure continuous focus on program adherence and improvement throughout the trial. Therefore, each fidelity review was followed up by a dialogue between team leaders and fidelity reviewers about action points for future implementation improvement. Secondly, fidelity reviews were done to document and benchmark implementation degree for each team throughout the trial-time. This enabled us to assess the risk of type III errors (wrongly rejecting a trial hypothesis of intervention superiority due to poor implementation),[1] which is often investigated in conjunction with RCTs.[2] This appendix addresses the latter aim of the fidelity reviews.

2.2 Method

Inspired by the fidelity review methods from *Individual Placement and Support* (IPS),[3] IBBIS fidelity reviews were designed as brief, standardized, multimethod investigations resulting in a fidelity-score on a predefined fidelity-scale.

2.2.1 Fidelity scale

The IBBIS fidelity scale was developed with 25 items measured on a Likert scale from one to five (total scale ranged from 25 to 125 points). Based on the IBBIS manuals and dialogue with intervention developers, the scale was designed to cover the most important activities in the IBBIS intervention. The scale was initially designed with six fidelity categories (organization, staff, integrated services, medical assessment, mental health care and vocational rehabilitation) that clustered similar items, see Table 2.

To benchmark the degree of implementation, three thresholds were decided on the 125-point scale:

- 74 points (49 %) or more equal fair implementation
- 100 points (75 %) or more equal good implementation
- 115 point (90 %) or more equal excellent implementation

2.2.2 Data material

Fidelity reviews were based on qualitative data material (primarily semi-structured interviews, observations and random samples of service user documents). IBBIS service users, professionals and managers were interviewed and observed. The fidelity reviews did not systematically utilize any of the data sources from the trials (e.g. self-assessment or management data). The fidelity review was conducted three times in each of the two trial sites (team city and team north).

2.2.3 Analysis of fidelity findings

To simplify the findings from the six fidelity reviews, we first reorganized the fidelity results of the six item-clusters to better fit the findings of the trial. Table 2 shows the original fidelity categories and the simplified trial categories.

Fidelity category	Fidelity item	Item description	Simplified trial category
**Organisation**	1	The IBBIS team	Integration
	2	Organizational integration	Integration
	3	Management support	Organization
	4	Team leader role	Integration
	5	Psychiatrist role	Organization
**Staff**	1	Case load	Organization
	2	Continuity in service	Organization
**Integrated services**	1	Collaboration through relational coordination	Integration
	2	Shared decision making	Integration
	3	Use of plans	Integration
**Medical assessment**	1	Medical assessment	Mental health care
**Mental health care**	1	Stepped Care	Mental health care
	2	Self-management	Mental health care
	3	Cognitive behavioural therapy	Mental health care
	4	Stress coaching	Mental health care
	5	MBSR	Mental health care
	6	Person involvement and relatives	Mental health care
**Vocational rehabilitation**	1	Work ability assessment	Vocational rehabilitation
	2	Voluntary disclosure	Vocational rehabilitation
	3	Ordinary work	Vocational rehabilitation
	4	Fast work focus	Vocational rehabilitation
	5	Individualized job search	Vocational rehabilitation
	6	Workplace contact	Vocational rehabilitation
	7	Collaboration with other municipal organs	Vocational rehabilitation
	8	Support beyond RTW	Vocational rehabilitation

Table 2: Fidelity items, fidelity categories and simplified trial categories

Secondly, we calculated average scores for the four simplified trial categories across time and teams. All averages were weighed according to the number of participants that were enrolled in team city and team north respectively. These weighed estimates were then converted into percentages to use the benchmarks for fair, good, and excellent fidelity.

2.3 Results

The six fidelity reviews were conducted from December 2016 to March 2018. The results are shown in Table 3.

		Team of review →	City			North		
	**Fidelity item ↓**	Time of review →	Dec., 2016	Sept., 2017	March, 2018	Dec., 2016	June, 2017	Dec., 2017
**Organisation**	1	The IBBIS team	3	2	2	4	2	3
	2	Organizational integration	3	3	4	3	3	3
	3	Management support	3	4	5	4	2	4
	4	Team leader role	1	1	4	3	4	5
	5	Psychiatrist role	4	5	5	4	5	4
**Staff**	1	Case load	4	4	4	5	4	5
	2	Continuity in service	4	4	4	4	4	4
**Integrated services**	1	Collaboration through relational coordination	2	3	4	3	4	4
	2	Shared decision making	3	4	4	4	5	5
	3	Use of plans	4	4	4	4	5	5
**Medical assessment**	1	Medical assessment	4	4	5	4	5	5
**Mental health care**	1	Stepped Care	5	5	4	5	5	5
	2	Self-management	5	4	3	5	4	4
	3	Cognitive behavioural therapy	5	5	5	5	5	5
	4	Stress coaching	4	5	5	4	5	5
	5	MBSR	4	4	5	4	3	4
	6	Person involvement and relatives	4	4	5	4	5	5
**Vocational rehabilitation**	1	Work ability assessment	4	4	4	4	4	4
	2	Voluntary disclosure	1	3	4	2	2	3
	3	Ordinary work	1	3	5	2	2	4
	4	Fast work focus	1	4	4	1	1	3
	5	Individualized job search	4	5	5	5	5	5
	6	Workplace contact	2	2	3	3	3	4
	7	Collaboration with other municipal organs	1	5	5	1	2	5
	8	Support beyond RTW	2	4	4	3	3	4
**Total**			**78**	**95**	**106**	**90**	**92**	**107**

Table 3: IBBIS fidelity results from the six fidelity reviews

The analysis of the four simplified fidelity scores showed that IBBIS mental health care was implemented with 87.8 % fidelity to the scale, whereas the IBBIS vocational rehabilitation was implemented with 56.3 % fidelity to the scale, see Table 4 4. Furthermore, integration of services was implemented 61.2 % fidelity to the scale.

Simplified fidelity category	Weighted average in percentage
Integration	61.2 %
IBBIS mental health care	87.8 %
IBBIS vocational rehabilitation	56.3 %
Organization	78.4 %

Table 4: Percentage implementation degree in simplified trial categories

2.4 Summary of fidelity results

According to the fidelity reviews, implementation degree rose throughout the trial time and was generally better in one of the teams. According to the average scores across teams and time, only the IBBIS mental health care was implemented with good fidelity, whereas the IBBIS vocational rehabilitation and integration of services were only just assessed to be implemented with fair fidelity.

3 References

1 Katz J, Wandersman A, Goodman RM, Griffin S, Wilson DK, Schillaci M. Updating the FORECAST formative evaluation approach and some implications for ameliorating theory failure, implementation failure, and evaluation failure. *Eval Program Plann* 2013; **39**: 42–50.

2 Hasson H. Intervention fidelity in clinical trials. In: Richards DA, Hallberg IR, eds. Complex Interventions in Health. An overview of research methods. New York: Routledge, 2015: 232–8.

3 Bonfils IS, Hansen H, Dalum HS, Eplov LF. Implementation of the individual placement and support approach–facilitators and barriers. *Scand J Disabil Res* 2017; **19**: 318–33.

Supplement 4: Sensitivity analyses report

Abbrev.: IBBIS: Integreret Behandlings- og BeskaeftigelsesIndsats til Sygedagpengemodtagere (English translation: Integrated Health Care and Vocational Rehabilitation for Sick Leave Benefit Recipients); HR: Hazard Ratio; INT: Integrated intervention; MHC: Mental health care; RCT: Randomized controlled trial; RTW: Return to work; SAU: Service as usual; OR: Odds Ratio; RR: Relative risk; FUn: n-month follow-up

Table legends: "Est.": Estimate "p": p-value "low/high CI": Low vs. high bounds of the 98.3% Confidence Interval of the estimate.

Legend: *: 0,05 > p > 0,016667 | **: p < 0,016667

In this document the term RCT2 refers to this RCT

This report chapter systematically displays all results from the IBBIS RCT trial covering stress related disorders.

Sensitivity analysis

All sensitivity analysis are performed using the entire group (consult the statistical analysis plan for details)

Sensitivity scenario: >best<

Kaplan Meier-curve at 12-month follow-up regarding: RCT2

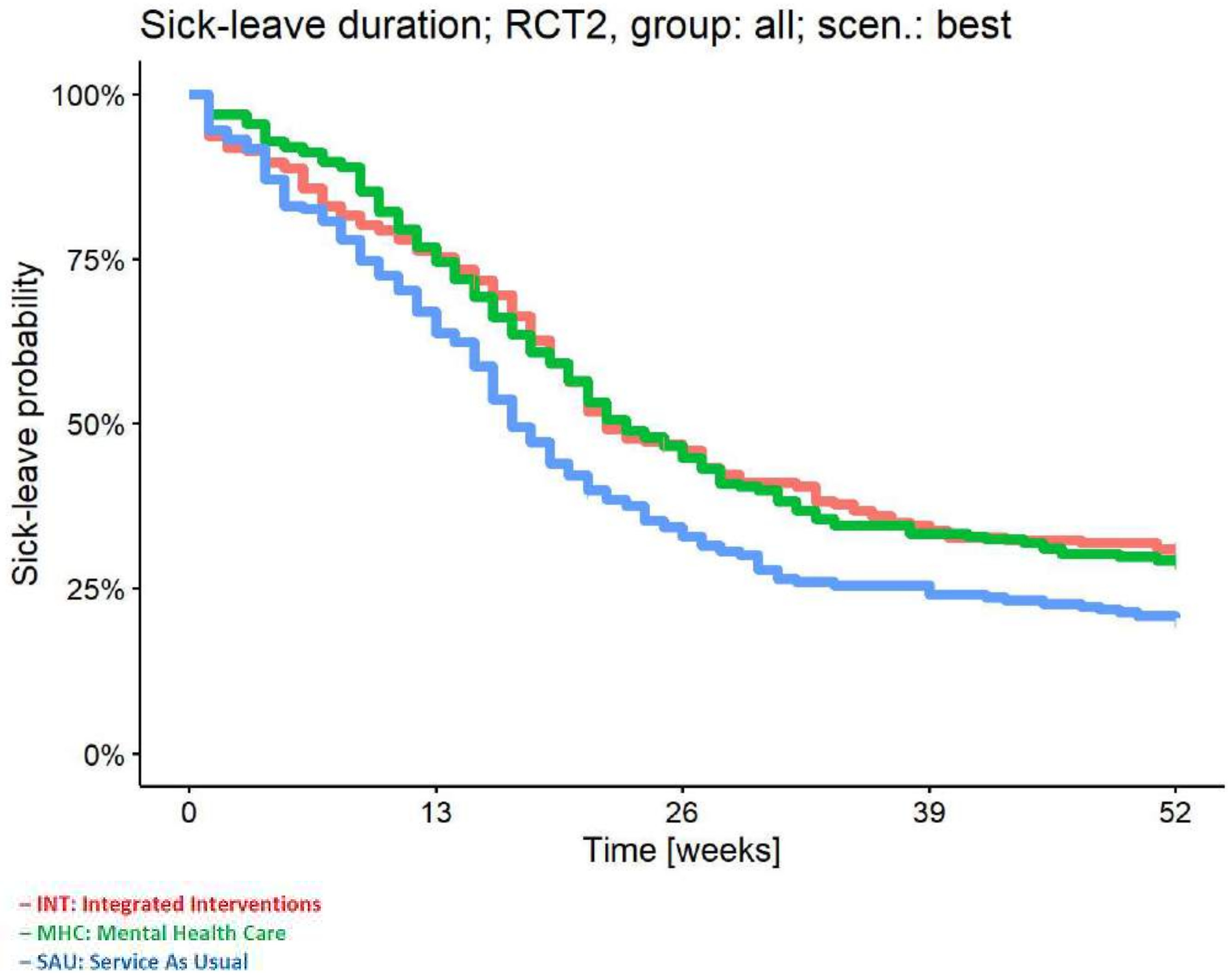

Vocational outcomes at 12-month follow-up from scenario >best< in RCT2

	Group values	SAU-MHC	SAU-INT	MHC-INT

Vocational outcomes at 12-month follow-up from scenario >best< in RCT2

	Group values			SAU-MHC				SAU-INT				MHC-INT			
	**INT**	**MHC**	**SAU**	**Est.**	**p**	**low CI**	**high CI**	**Est.**	**p**	**low CI**	**high CI**	**Est.**	**p**	**low CI**	**high CI**
RTW, FU12 [weeks, HR]	22.0	23.0	17.0	1.35	**0.0077	1.03	1.78	1.43	**0.002	1.08	1.89	1.06	0.60	0.80	1.41
RTW, FU6 [HR]				1.41	**0.0069	1.04	1.91	1.51	**0.0015	1.10	2.06	1.07	0.59	0.78	1.48
PROP, FU12 [%, OR]	56.6	47.7	62.2	1.76	**0.0053	1.08	2.85	1.26	0.27	0.76	2.08	0.71	0.10	0.44	1.17
WEEKS, FU12 [weeks, RR]	21.3	20.0	25.5	1.24	**0.003	1.04	1.47	1.22	**0.007	1.02	1.45	0.99	0.87	0.82	1.19

Self-reported outcomes at 24-month follow-up regarding scenario: best (RCT2)

	Group values						SAU-MHC				SAU-INT				MHC-INT			
	**INT**	**(SD)**	**MHC**	**(SD)**	**SAU**	**(SD)**	**Est.**	**p**	**low CI**	**high CI**	**Est.**	**p**	**low CI**	**high CI**	**Est.**	**p**	**low CI**	**high CI**
BAI [FU6]1	7.6	(8.48)	6.16	(8.19)	7.49	(9.62)	1.65	*0.0377	-0.25	3.55	-0.02	0.98	-2.01	1.97	-1.7	*0.0294	-3.58	0.17
BDI [FU6]1	8.42	(9.94)	6.89	(9.61)	7.34	(10.52)	0.85	0.34	-1.3	3.01	-0.92	0.31	-3.09	1.24	-1.75	*0.0446	-3.84	0.34
PSS [FU6]1	13.77	(8.12)	11.82	(7.83)	12.78	(8.7)	1.2	0.07	-0.41	2.81	-1.02	0.14	-2.65	0.62	-2.21	**7e-04	-3.76	-0.66
KES [FU6]1	55.71	(20.22)	52.77	(20)	55.12	(22.84)	3.23	0.06	-0.88	7.34	-0.29	0.87	-4.55	3.97	-3.46	*0.0355	-7.39	0.48
DSQ-som. [FU6]1	5.45	(7.34)	5.09	(6.88)	5.25	(8.45)	0.54	0.42	-1.06	2.13	-0.18	0.79	-1.85	1.49	-0.74	0.24	-2.24	0.76
DSQ-distr. [FU6]1	7.58	(8.66)	7.28	(8.46)	7.39	(9.67)	0.55	0.46	-1.23	2.34	-0.22	0.77	-2.02	1.58	-0.73	0.3	-2.43	0.97
DSQ-anx. [FU6]1	0.75	(4.23)	0.65	(4.15)	0.59	(4.73)	0.16	0.69	-0.82	1.14	-0.15	0.71	-1.13	0.83	-0.32	0.42	-1.25	0.62
DSQ-depr. [FU6]1	0.43	(2.86)	0.24	(2.49)	0	(2.67)	-0.12	0.6	-0.7	0.45	-0.46	0.06	-1.05	0.14	-0.32	0.2	-0.9	0.27
WSAS [FU6]1	9.35	(10.93)	7.64	(11.07)	8.23	(12.34)	1.07	0.26	-1.22	3.35	-1.01	0.29	-3.33	1.3	-2.08	*0.022	-4.25	0.1
SPS [FU6]1	22.32	(3.36)	22.4	(3.54)	22.61	(3.39)	0.25	0.44	-0.53	1.04	0.3	0.35	-0.46	1.05	0.03	0.94	-0.76	0.81
IPQ [FU6]1	18.79	(4.2)	18.69	(4.44)	19.27	(4.62)	0.54	0.17	-0.4	1.48	0.46	0.24	-0.49	1.42	-0.07	0.85	-0.99	0.85
GSS [FU6]1	30.84	(7.85)	31.59	(7.75)	32.4	(7.65)	0.68	0.31	-0.93	2.29	1.55	*0.0265	-0.12	3.23	0.87	0.21	-0.78	2.51
RTW-SE [FU6]1	28.98	(10.75)	30.02	(10.65)	30.61	(11.34)	0.29	0.75	-1.91	2.5	1.59	0.08	-0.62	3.8	1.32	0.14	-0.82	3.45
QoLs [FU6]1	68.48	(16.64)	68.23	(17.14)	66.7	(17.63)	-1.6	0.28	-5.15	1.95	-2.32	0.11	-5.78	1.14	-0.7	0.62	-4.1	2.7
EQ5 [FU6]1	0.87	(0.16)	0.89	(0.15)	0.89	(0.17)	-0.01	0.71	-0.04	0.03	0.02	0.16	-0.01	0.05	0.02	0.07	-0.01	0.06
IS [FU6]1	27.3	(5.96)	27.29	(5.99)	25.59	(7.9)	-1.83	**0.0068	-3.44	-0.21	-1.69	**0.0145	-3.34	-0.04	0.1	0.87	-1.29	1.48
BAI [FU12]1	5.92	(8.41)	5.12	(8.58)	5.36	(10.21)	0.51	0.53	-1.45	2.48	-0.39	0.64	-2.4	1.61	-0.91	0.24	-2.78	0.96
BDI [FU12]1	6.09	(9.73)	5.23	(10.49)	3.84	(9.82)	-1.09	0.23	-3.27	1.09	-2.11	*0.0187	-4.27	0.04	-0.97	0.28	-3.11	1.17
PSS [FU12]1	11.43	(8.4)	11.01	(8.82)	10.39	(8.56)	-0.39	0.59	-2.11	1.33	-0.93	0.19	-2.65	0.79	-0.52	0.45	-2.2	1.16

	Group values						SAU-MHC				SAU-INT				MHC-INT			
	**INT**	**(SD)**	**MHC**	**(SD)**	**SAU**	**(SD)**	**Est.**	**p**	**low CI**	**high CI**	**Est.**	**p**	**low CI**	**high CI**	**Est.**	**p**	**low CI**	**high CI**
KES [FU12]1	46.69	(20.8)	44.15	(21.07)	43.81	(22.61)	0.27	0.88	-4.08	4.61	-2.6	0.16	-7.02	1.83	-2.83	0.1	-7	1.33
DSQ-som. [FU12]1	4.48	(7.07)	4.37	(7.31)	4.12	(8.23)	0.05	0.95	-1.6	1.69	-0.13	0.86	-1.83	1.58	-0.19	0.77	-1.75	1.37
DSQ-distr. [FU12]1	6.83	(8.39)	6.6	(8.94)	5.5	(8.84)	-0.78	0.31	-2.63	1.07	-1.11	0.15	-2.98	0.75	-0.31	0.68	-2.11	1.49
DSQ-anx. [FU12]1	0.39	(3.71)	0.22	(3.8)	-0.07	(4.41)	-0.13	0.75	-1.09	0.84	-0.34	0.4	-1.31	0.63	-0.21	0.58	-1.13	0.7
DSQ-depr. [FU12]1	0.1	(2.65)	0.1	(2.89)	-0.56	(2.44)	-0.57	*0.0198	-1.15	0.02	-0.61	**0.0144	-1.2	-0.01	-0.02	0.93	-0.63	0.58
WSAS [FU12]1	6.79	(11.15)	5.89	(11.39)	4.47	(11.53)	-1.08	0.28	-3.44	1.29	-2.15	*0.0291	-4.52	0.21	-1.07	0.26	-3.34	1.2
SPS [FU12]1	21.96	(4.12)	21.98	(4.52)	22.37	(4.36)	0.23	0.57	-0.74	1.19	0.26	0.5	-0.64	1.15	0.01	0.98	-0.9	0.92
IPQ [FU12]1	19.25	(4.68)	19.5	(4.91)	20.04	(4.74)	0.44	0.28	-0.54	1.43	0.77	0.06	-0.21	1.75	0.33	0.42	-0.64	1.3
GSS [FU12]1	31.94	(8.17)	33.23	(7.71)	34.15	(8.12)	0.79	0.25	-0.87	2.44	2.2	**0.0022	0.48	3.92	1.39	*0.0467	-0.29	3.07
RTW-SE [FU12]1	32.54	(10.68)	33.4	(10.99)	34.49	(11.02)	0.84	0.38	-1.44	3.11	1.84	0.05	-0.42	4.11	1.04	0.26	-1.17	3.25
QoLs [FU12]1	70.74	(16.36)	68.26	(17.23)	67.71	(16.85)	-0.28	0.85	-3.9	3.35	-3.15	*0.0312	-6.66	0.35	-2.86	*0.0484	-6.33	0.61
EQ5 [FU12]1	0.89	(0.16)	0.91	(0.17)	0.92	(0.17)	0.01	0.52	-0.03	0.04	0.04	**0.0122	0	0.07	0.03	0.06	-0.01	0.06

Sensitivity scenario: >worst<

Kaplan Meier-curve at 12-month follow-up regarding: RCT2

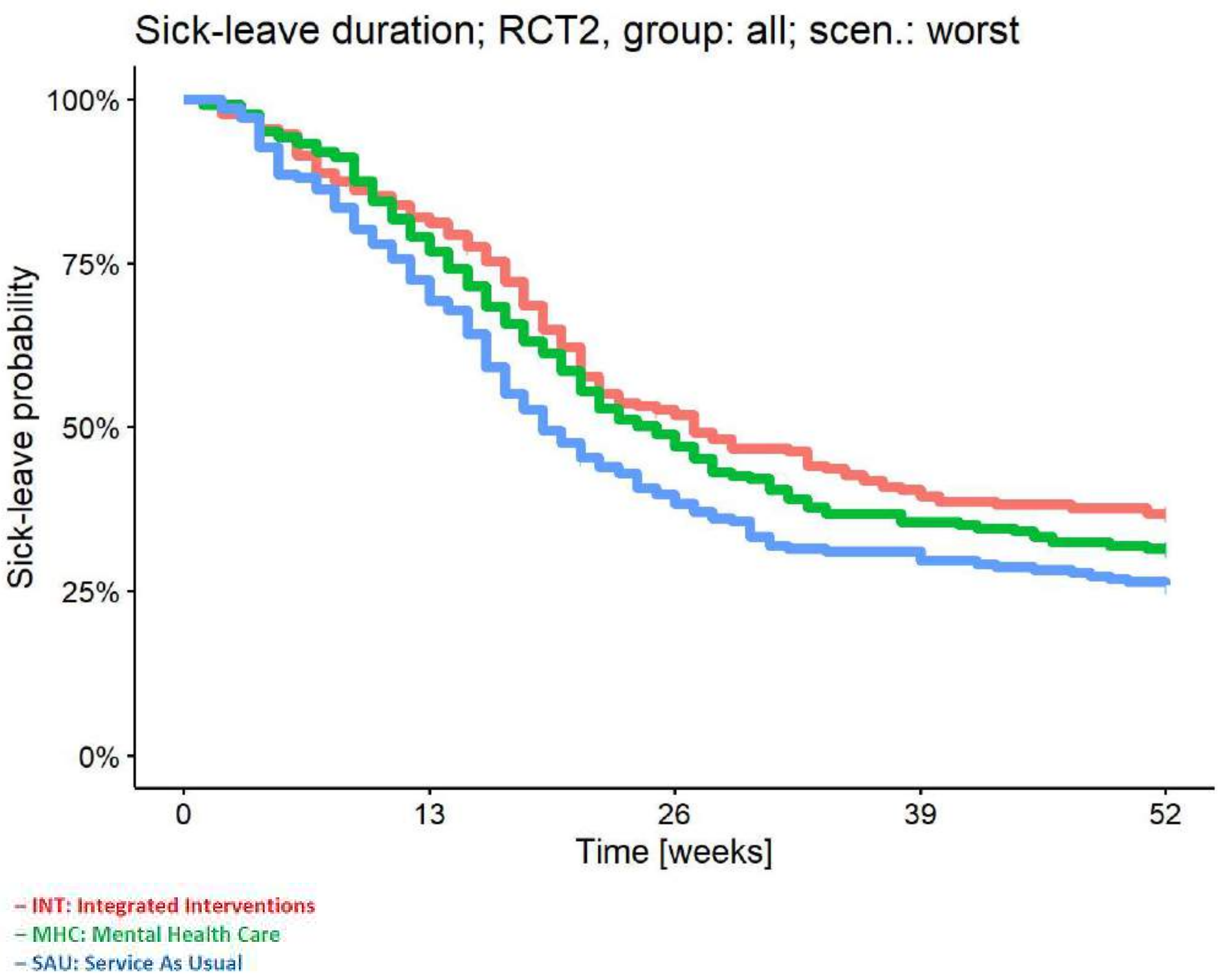

Vocational outcomes at 12-month follow-up from scenario >worst< in RCT2

	Group values			SAU-MHC					SAU-INT					MHC-INT				
	INT	MHC	SAU	Est.	p	low CI	:	high CI	Est.	p	low CI	:	high CI	Est.	p	low CI	:	high CI
RTW, FU12 [weeks, HR]	27.0	25.0	19.0	1.35	**0.0077	1.03		1.78	1.43	**0.002	1.08		1.89	1.06	0.60	0.80		1.41
RTW, FU6 [HR]				1.41	**0.0069	1.04		1.91	1.51	**0.0015	1.10		2.06	1.07	0.59	0.78		1.48
PROP, FU12 [%, OR]	50.7	45.5	56.7	1.76	**0.0053	1.08		2.85	1.26	0.27	0.76		2.08	0.71	0.10	0.44		1.17
WEEKS, FU12 [weeks, RR]	18.3	18.9	22.6	1.24	**0.003	1.04		1.47	1.22	**0.007	1.02		1.45	0.99	0.87	0.82		1.19

Self-reported outcomes at 24-month follow-up regarding scenario: worst (RCT2)

	Group values						SAU-MHC				SAU-INT				MHC-INT			
	INT	(SD)	MHC	(SD)	SAU	(SD)	Est.	p	low CI	high CI	Est.	p	low CI	high CI	Est.	p	low CI	high CI
BAI [FU6]1	11.35	(8.5)	10.81	(8.77)	13.8	(8.85)	2.94	**2e-04	1.05	4.82	2.53	**0.0016	0.61	4.46	-0.46	0.56	-2.34	1.43
BDI [FU6]1	12.56	(9.86)	12.21	(10.02)	14.39	(10.13)	2.17	**0.0157	0.02	4.32	2	*0.0235	-0.12	4.11	-0.17	0.84	-2.26	1.92
PSS [FU6]1	17.16	(7.97)	16.29	(8.42)	18.94	(8.16)	2.55	**2e-04	0.92	4.17	1.79	**0.0068	0.21	3.36	-0.78	0.23	-2.36	0.79
KES [FU6]1	67.29	(20.43)	66.74	(21.42)	73.7	(20.66)	7.1	**0	2.99	11.2	6.81	**1e-04	2.76	10.86	-0.33	0.84	-4.35	3.69

	Group values						SAU-MHC				SAU-INT				MHC-INT			
	**INT**	**(SD)**	**MHC**	**(SD)**	**SAU**	**(SD)**	**Est.**	**p**	**low CI**	**high CI**	**Est.**	**p**	**low CI**	**high CI**	**Est.**	**p**	**low CI**	**high CI**
DSQ-som. [FU6]1	10.26	(7.47)	10.08	(7.21)	12.24	(7.86)	1.91	**0.0038	0.33	3.49	2.21	**0.0011	0.59	3.84	0.25	0.69	-1.27	1.76
DSQ-distr. [FU6]1	13.63	(8.84)	13.53	(8.8)	15.85	(8.98)	2.04	**0.0059	0.26	3.82	2.46	**8e-04	0.71	4.21	0.41	0.56	-1.3	2.13
DSQ-anx. [FU6]1	3.58	(4.31)	3.52	(4.29)	4.68	(4.42)	1.01	**0.0123	0.04	1.98	1.23	**0.0019	0.28	2.18	0.19	0.63	-0.75	1.12
DSQ-depr. [FU6]1	2.13	(2.81)	1.97	(2.55)	2.47	(2.64)	0.4	0.1	-0.18	0.97	0.4	0.1	-0.19	0.98	0	1	-0.58	0.58
WSAS [FU6]1	14.38	(10.92)	14	(11.63)	17.45	(11.56)	3.49	**3e-04	1.18	5.8	3.24	**6e-04	0.98	5.5	-0.27	0.77	-2.48	1.93
SPS [FU6]1	16.84	(3.33)	16.67	(3.44)	16.75	(3.53)	0.16	0.64	-0.65	0.96	-0.04	0.92	-0.84	0.77	-0.18	0.58	-0.95	0.6
IPQ [FU6]1	16.29	(4.3)	15.95	(4.41)	15.18	(4.49)	-0.75	0.05	-1.68	0.18	-1.16	**0.0033	-2.11	-0.22	-0.38	0.32	-1.3	0.53
GSS [FU6]1	26.24	(7.67)	26.57	(7.91)	25.33	(7.68)	-1.23	0.07	-2.85	0.39	-0.92	0.18	-2.57	0.73	0.32	0.63	-1.3	1.94
RTW-SE [FU6]1	24.34	(10.74)	23.94	(10.81)	22.3	(11.16)	-1.5	0.1	-3.72	0.72	-2.13	*0.0195	-4.31	0.06	-0.56	0.53	-2.71	1.59
QoLs [FU6]1	79.55	(17)	81.45	(16.92)	82.92	(17.39)	1.01	0.48	-2.44	4.47	2.88	*0.0488	-0.62	6.38	1.77	0.22	-1.66	5.21
EQ5 [FU6]1	0.79	(0.16)	0.79	(0.16)	0.76	(0.16)	-0.03	*0.0202	-0.07	0	-0.03	**0.0138	-0.07	0	0	0.97	-0.03	0.03
IS [FU6]1	23.33	(6.62)	22.76	(6.53)	19.3	(6.26)	-3.45	**0	-4.94	-1.97	-4.07	**0	-5.57	-2.57	-0.58	0.35	-2.09	0.92
BAI [FU12]1	11.28	(8.58)	11.63	(8.95)	13.99	(9.5)	2.23	**0.0055	0.31	4.16	2.82	**5e-04	0.89	4.74	0.56	0.48	-1.33	2.44
BDI [FU12]1	12.02	(9.58)	12.44	(10.48)	13.25	(10.07)	0.66	0.47	-1.52	2.85	1.29	0.15	-0.83	3.41	0.65	0.46	-1.47	2.76
PSS [FU12]1	16.51	(8.52)	17.17	(8.88)	18.56	(8.27)	1.24	0.08	-0.48	2.96	2.11	**0.0025	0.44	3.78	0.86	0.22	-0.83	2.55
KES [FU12]1	61.12	(20.92)	61.24	(22.17)	67.55	(20.84)	6.05	**8e-04	1.72	10.38	6.67	**2e-04	2.43	10.9	0.62	0.73	-3.63	4.87
DSQ-som. [FU12]1	10.29	(7.31)	10.45	(7.54)	12.41	(7.62)	1.77	**0.0094	0.14	3.39	2.1	**0.0022	0.46	3.75	0.31	0.63	-1.26	1.88
DSQ-distr. [FU12]1	13.19	(8.49)	13.53	(9.01)	15.25	(8.59)	1.48	0.05	-0.37	3.33	2.01	**0.0081	0.19	3.82	0.54	0.47	-1.26	2.34
DSQ-anx. [FU12]1	3.28	(3.8)	3.37	(3.93)	4.49	(4.13)	1.02	**0.0103	0.07	1.97	1.19	**0.0023	0.25	2.12	0.17	0.66	-0.75	1.08
DSQ-depr. [FU12]1	2.02	(2.64)	2.23	(2.81)	2.4	(2.59)	0.08	0.74	-0.51	0.67	0.35	0.15	-0.24	0.94	0.28	0.26	-0.31	0.87
WSAS [FU12]1	13.5	(11.09)	14.2	(11.39)	15.45	(11.62)	1.08	0.28	-1.31	3.47	2.04	*0.0357	-0.29	4.38	0.95	0.32	-1.33	3.23

	Group values						SAU-MHC				SAU-INT				MHC-INT			
	**INT**	**(SD)**	**MHC**	**(SD)**	**SAU**	**(SD)**	**Est.**	**p**	**low CI**	**high CI**	**Est.**	**p**	**low CI**	**high CI**	**Est.**	**p**	**low CI**	**high CI**
SPS [FU12]1	16.38	(4.39)	15.4	(4.1)	15.71	(4.47)	0.29	0.46	-0.65	1.22	-0.68	0.08	-1.61	0.25	-0.94	**0.0118	-1.84	-0.05
IPQ [FU12]1	16.11	(4.76)	15.63	(4.91)	14.83	(4.59)	-0.7	0.08	-1.68	0.27	-1.28	**0.0016	-2.25	-0.31	-0.57	0.16	-1.54	0.39
GSS [FU12]1	26.87	(7.89)	27.04	(7.94)	25.84	(8.27)	-1.03	0.14	-2.71	0.65	-1.02	0.15	-2.7	0.66	0.02	0.98	-1.63	1.67
RTW-SE [FU12]1	25.23	(10.74)	24.42	(11.09)	22.62	(10.77)	-1.61	0.09	-3.9	0.68	-2.67	**0.0043	-4.91	-0.43	-0.99	0.28	-3.22	1.23
QoLs [FU12]1	81.54	(16.36)	82.62	(17.81)	86.04	(16.04)	2.98	*0.0444	-0.57	6.53	4.37	**0.0029	0.85	7.89	1.33	0.37	-2.22	4.88
EQ5 [FU12]1	0.79	(0.16)	0.78	(0.17)	0.75	(0.16)	-0.03	0.05	-0.06	0.01	-0.04	**0.0062	-0.07	0	-0.01	0.45	-0.04	0.02

Supplement 5: Subgroup analyses report

Abbrev.: IBBIS: Integreret Behandlings- og BeskaeftigelsesIndsats til Sygedagpengemodtagere (English translation: Integrated Health Care and Vocational Rehabilitation for Sick Leave Benefit Recipients); HR: Hazard Ratio; INT: Integrated intervention; MHC: Mental health care; RCT: Randomized controlled trial; RTW: Return to work; SAU: Service as usual; OR: Odds Ratio; RR: Relative risk; FUn: n-month follow-up

Table legends: “Est.”: Estimate “p”: p-value “low/high CI”: Low vs. high bounds of the 98.3% Confidence Interval of the estimate.

Subgroup abbrev.:

The following abbreviations reflects subgroups including only participants with following characteristics:

- “ex”: Exhaustion disorder,
- “stress”: Psychological distress,
- “adj”: Adjustment disorder,
- “emp”: Employment (at baseline),
- “vac”: Unemployed (at baseline),
- “north”: Team North,
- “city”: Team City (Copenhagen),
- “first”: All participants randomized in the first temporal half of the RCT,
- “last”: All participants randomized in the last temporal half of the RCT,
- “interaction”: Analyses adjusted for the diagnosis x treatment arm interaction
- “intempl”: Analyses adjusted for the employment status (employed vs uempl.) x treatment arm interaction
- “intteam”: Analyses adjusted for the intervention-team (Team North vs. City) x treatment arm interaction
- “inttemp”: Analyses adjusted for the time (first vs. last trial period half) x treatment arm interaction

Legend: *: 0,05 > p > 0,016667 | **: p < 0,016667

This report chapter systematically displays all results from the IBBIS RCT trial covering stress related disorders.

Analyses regarding: >adj<

Kaplan Meier-curve at 12-month follow-up regarding: adj

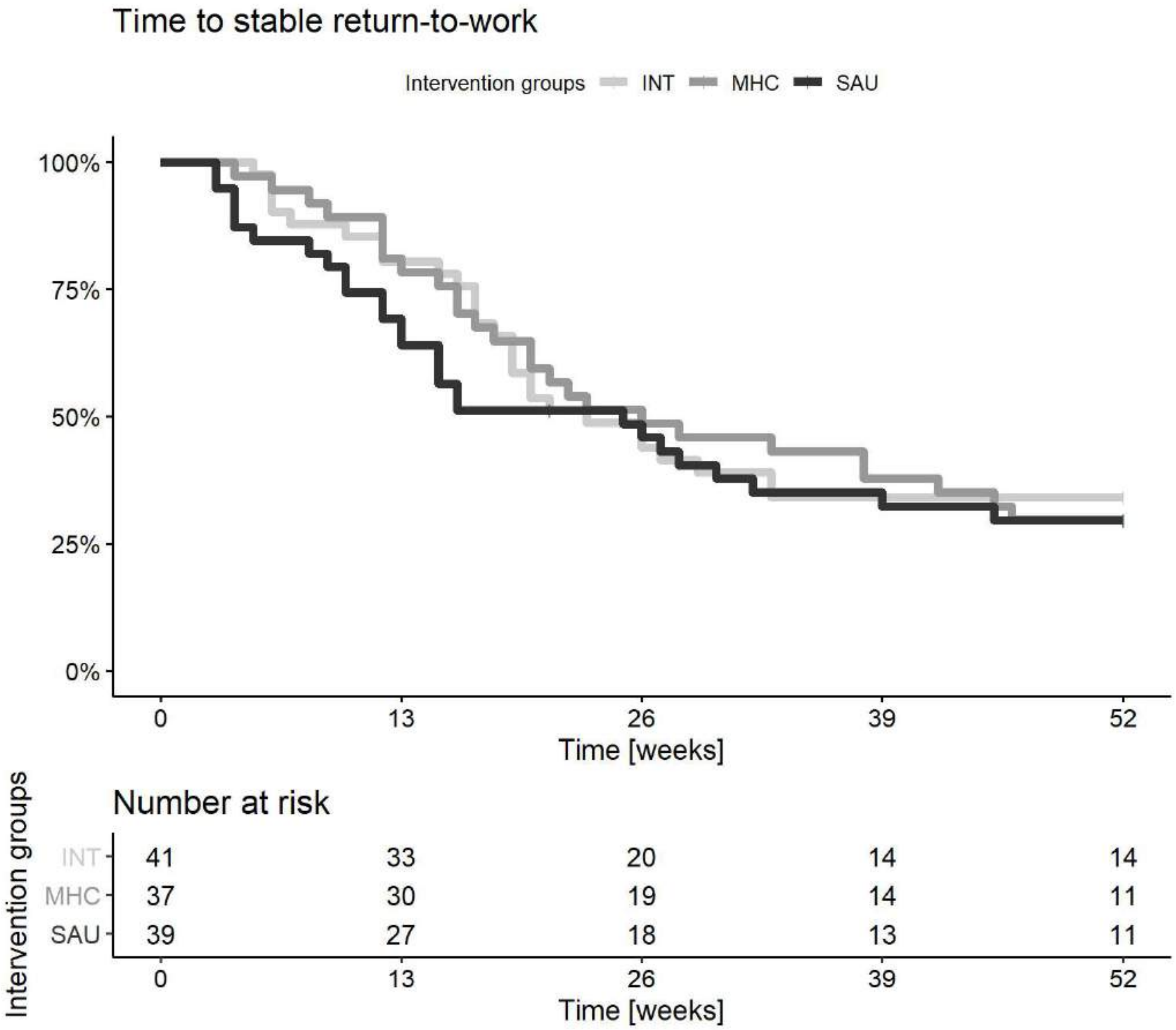

Proportion over time-curve at 12-month follow-up regarding: adj<

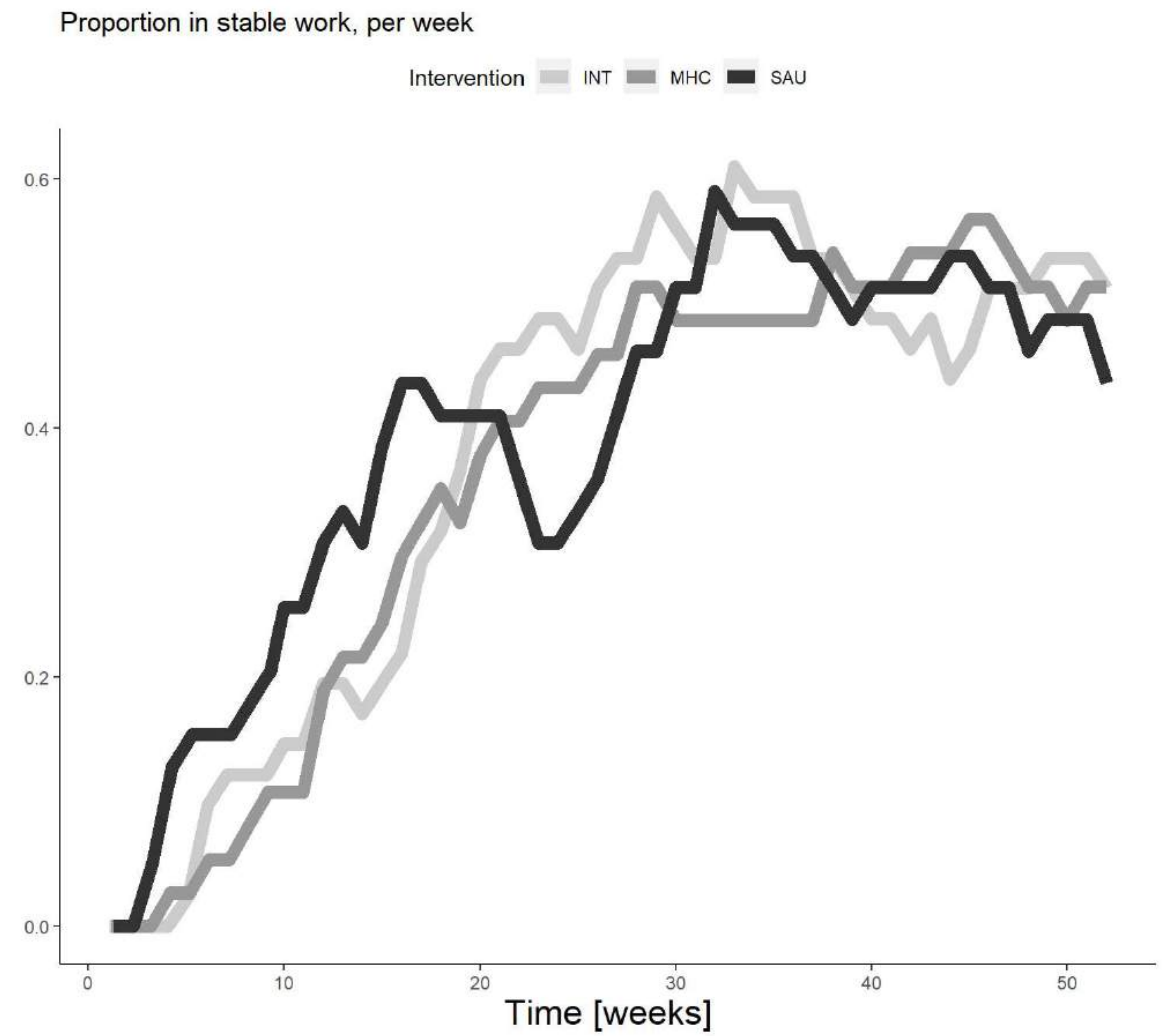

Vocational outcomes at 12-month follow-up from subgroup >adj< in RCT2

	Group values			SAU-MHC				SAU-INT				MHC-INT			
	INT	MHC	SAU	Est.	p	low CI	high CI	Est.	p	low CI	high CI	Est.	p	low CI	high CI
RTW, FU12 [weeks, HR]	23	26	25	1.15	0.61	0.58	2.27	1.32	0.32	0.67	2.58	1.14	0.64	0.58	2.24
RTW, FU6 [HR]				1.18	0.6	0.54	2.57	1.2	0.54	0.57	2.53	1.01	0.97	0.47	2.17
PROP, FU12 [%, OR]	51.2	51.4	44.7	0.61	0.33	0.18	2.13	0.77	0.59	0.23	2.55	1.16	0.76	0.35	3.84
WEEKS, FU12 [weeks, RR]	20.1	19.3	21	1.03	0.88	0.67	1.57	1.06	0.74	0.7	1.58	1.04	0.85	0.67	1.61

Self-reported outcomes at 6- and 12-month follow-up regarding: adj

	Group values						SAU-MHC				SAU-INT				MHC-INT			
	INT	(SD)	MHC	(SD)	SAU	(SD)	Est.	p	low CI	high CI	Est.	p	low CI	high CI	Est.	p	low CI	high CI
BAI [FU6]	9.25	(7.31)	7.07	(5.57)	9.71	(5.77)	1.68	0.32	-2.41	5.78	0.4	0.82	-3.75	4.54	-1.36	0.42	-5.48	2.75
BDI [FU6]	10.57	(9.51)	7.17	(7.63)	8.47	(6.63)	0.67	0.71	-3.76	5.1	-1.69	0.36	-6.11	2.74	-2.65	0.19	-7.51	2.21
PSS [FU6]	14.97	(7.12)	12.61	(6.79)	13.67	(6.53)	0.68	0.65	-2.97	4.32	-1	0.47	-4.35	2.35	-1.8	0.22	-5.35	1.75
KES [FU6]	62.67	(17.62)	53.58	(17.21)	59.81	(19.77)	4.06	0.28	-5.06	13.18	-2.37	0.49	-10.73	5.99	-6.55	0.06	-14.8	1.7
DSQ-som. [FU6]	7.73	(6.27)	5.63	(4.23)	6.96	(5.47)	1.65	0.29	-2.1	5.39	-0.26	0.86	-3.84	3.33	-1.68	0.21	-4.95	1.59
DSQ-distr. [FU6]	10.69	(7.21)	9.38	(7.55)	9.15	(6.47)	0.11	0.94	-3.52	3.73	-1.03	0.47	-4.48	2.43	-1.33	0.37	-4.87	2.22

	Group values						SAU-MHC				SAU-INT				MHC-INT			
	**INT**	**(SD)**	**MHC**	**(SD)**	**SAU**	**(SD)**	**Est.**	**p**	**low CI**	**high CI**	**Est.**	**p**	**low CI**	**high CI**	**Est.**	**p**	**low CI**	**high CI**
DSQ-anx. [FU6]	2.14	(3.23)	1.78	(2.44)	2	(2.42)	0.34	0.67	-1.57	2.25	0.05	0.95	-1.94	2.04	-0.33	0.69	-2.29	1.64
DSQ-depr. [FU6]	1.41	(2.55)	0.96	(1.79)	0.5	(0.81)	-0.3	0.5	-1.37	0.77	-0.77	0.12	-1.99	0.44	-0.57	0.31	-1.92	0.78
WSAS [FU6]	10.56	(9.78)	7.61	(10.16)	11.31	(9.62)	2.45	0.29	-3.15	8.04	0.79	0.7	-4.18	5.76	-1.72	0.42	-6.94	3.5
SPS [FU6]	19.83	(2.71)	19.43	(3.11)	20.15	(2.76)	0.14	0.65	-0.61	0.89	0.12	0.69	-0.6	0.84	-0.02	0.93	-0.69	0.64
IPQ [FU6]	17.44	(3.57)	17.65	(3.9)	17.28	(4.5)	-0.51	0.58	-2.74	1.73	0.03	0.97	-2.13	2.19	0.46	0.58	-1.58	2.5
GSS [FU6]	27.78	(6.36)	29.76	(7.71)	30.23	(4.39)	0.27	0.85	-3.22	3.76	1.75	0.2	-1.51	5.01	1.36	0.36	-2.26	4.98
RTW-SE [FU6]	28.7	(9.1)	29.75	(8.88)	27.73	(9.08)	-1.32	0.53	-6.36	3.72	-0.96	0.62	-5.61	3.68	0.58	0.77	-4.17	5.33
QoLs [FU6]	74.79	(13.85)	76.32	(14.65)	80.52	(11.09)	1.8	0.57	-5.89	9.48	3.24	0.26	-3.71	10.2	1.27	0.68	-6.15	8.7
EQ5 [FU6]	0.85	(0.13)	0.86	(0.17)	0.86	(0.17)	0	0.95	-0.09	0.09	0.01	0.85	-0.06	0.07	0	0.89	-0.08	0.09
IS [FU6]	24.09	(5.84)	25.65	(5.73)	21.48	(5.22)	-3.02	*0.0494	-6.74	0.7	-1.71	0.24	-5.26	1.83	1.54	0.27	-1.87	4.96
BAI [FU12]	9.22	(7.84)	9.08	(8.92)	7.96	(5.06)	-0.48	0.78	-4.65	3.68	-0.42	0.81	-4.51	3.67	0.01	0.99	-4.25	4.28
BDI [FU12]	8.72	(9.5)	7.28	(8.92)	6.15	(5.38)	-0.96	0.61	-5.55	3.63	-1.97	0.3	-6.58	2.64	-1.24	0.55	-6.28	3.8
PSS [FU12]	13.56	(8.02)	13.46	(8.56)	13.52	(5.04)	-0.15	0.92	-4.05	3.75	-0.22	0.89	-3.92	3.49	-0.2	0.9	-4.15	3.74
KES [FU12]	51.59	(17.25)	48.5	(19.24)	49.91	(14.68)	0.46	0.9	-8.7	9.62	-1.33	0.71	-9.8	7.15	-1.87	0.61	-10.72	6.97
DSQ-som. [FU12]	8.09	(5.89)	6.5	(6.25)	6.86	(5.16)	0.64	0.68	-3.06	4.33	-0.5	0.73	-4.09	3.08	-0.9	0.53	-4.36	2.55
DSQ-distr. [FU12]	9.68	(7.19)	8.43	(8.03)	9.26	(5.16)	0.26	0.87	-3.43	3.95	-1	0.5	-4.56	2.57	-1.48	0.36	-5.33	2.38
DSQ-anx. [FU12]	2.12	(3.29)	1.33	(2.93)	1.26	(1.45)	0.06	0.94	-1.71	1.83	-0.37	0.62	-2.17	1.44	-0.44	0.57	-2.33	1.45
DSQ-depr. [FU12]	1.25	(2.79)	1.08	(2.81)	0.57	(1.24)	-0.48	0.3	-1.61	0.65	-0.69	0.17	-1.91	0.53	-0.28	0.63	-1.69	1.13
WSAS [FU12]	7.97	(8.83)	8	(11)	7.48	(9.13)	-0.38	0.87	-6.02	5.26	0.13	0.95	-5.01	5.28	0.35	0.88	-5.07	5.77
SPS [FU12]	18.92	(3.5)	19	(3.12)	18	(3.98)	0.02	0.96	-0.92	0.96	-0.37	0.32	-1.26	0.52	-0.37	0.29	-1.22	0.47
IPQ [FU12]	18.16	(3.85)	17.61	(5.2)	17.61	(3.07)	-0.09	0.92	-2.42	2.23	-0.51	0.57	-2.71	1.68	-0.49	0.6	-2.73	1.76
GSS [FU12]	30.41	(6.61)	31.38	(7.14)	30.91	(5.19)	0.16	0.92	-3.47	3.78	0.65	0.64	-2.73	4.02	0.38	0.8	-3.32	4.08
RTW-SE [FU12]	30.61	(9.19)	32.57	(8.22)	29.27	(8.3)	-1.19	0.56	-6.15	3.77	-0.59	0.77	-5.47	4.29	0.73	0.72	-4.18	5.64
QoLs [FU12]	78.69	(14.33)	78.43	(16.93)	79.22	(11.39)	1.12	0.72	-6.43	8.68	0.77	0.79	-6.21	7.75	-0.58	0.85	-8.11	6.94
EQ5 [FU12]	0.85	(0.12)	0.84	(0.2)	0.87	(0.13)	0.02	0.69	-0.08	0.11	0.01	0.79	-0.07	0.08	-0.01	0.77	-0.09	0.07

Analyses regarding: >ex<

Kaplan Meier-curve at 12-month follow-up regarding: ex

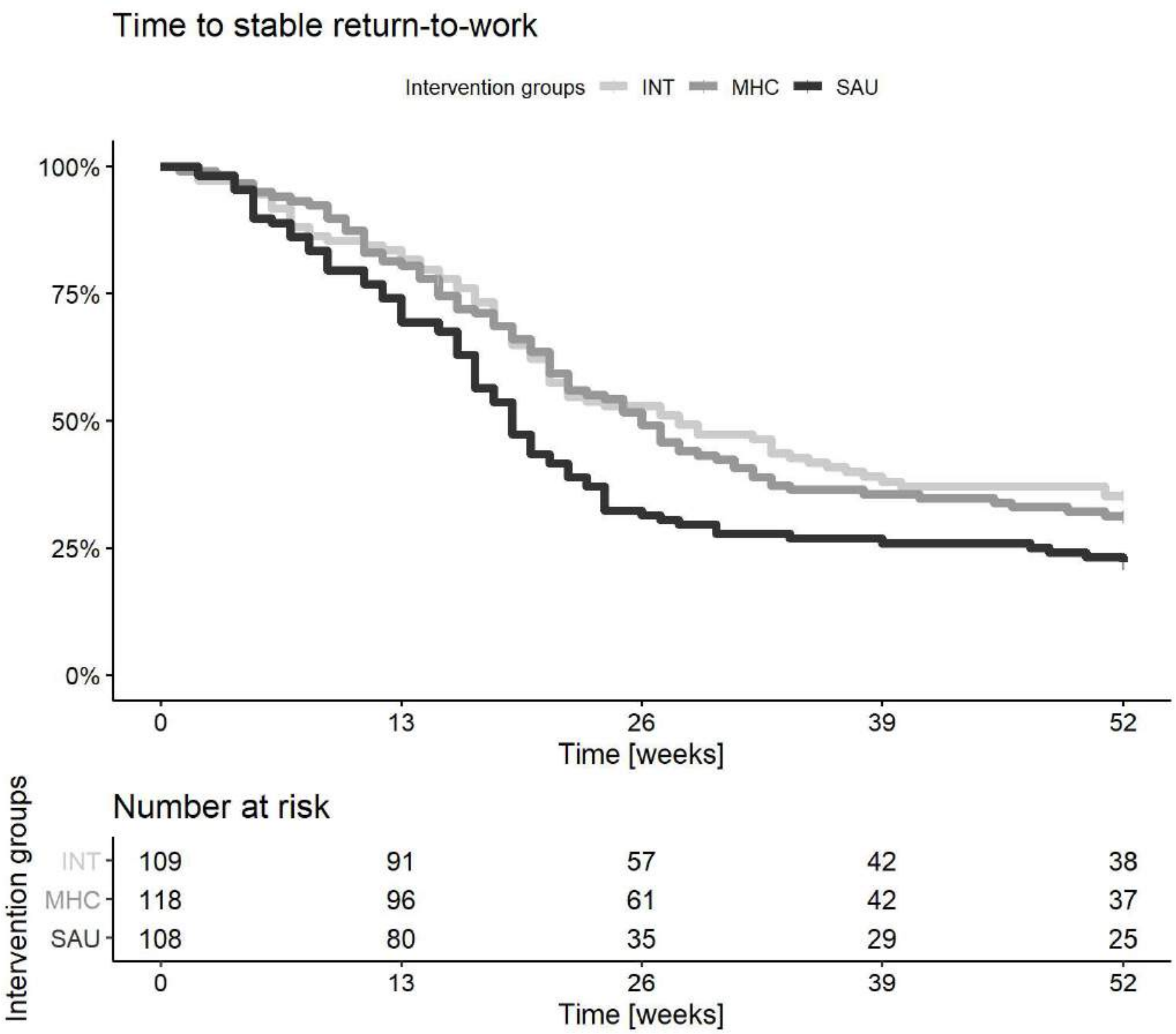

Proportion over time-curve at 12-month follow-up regarding: ex<

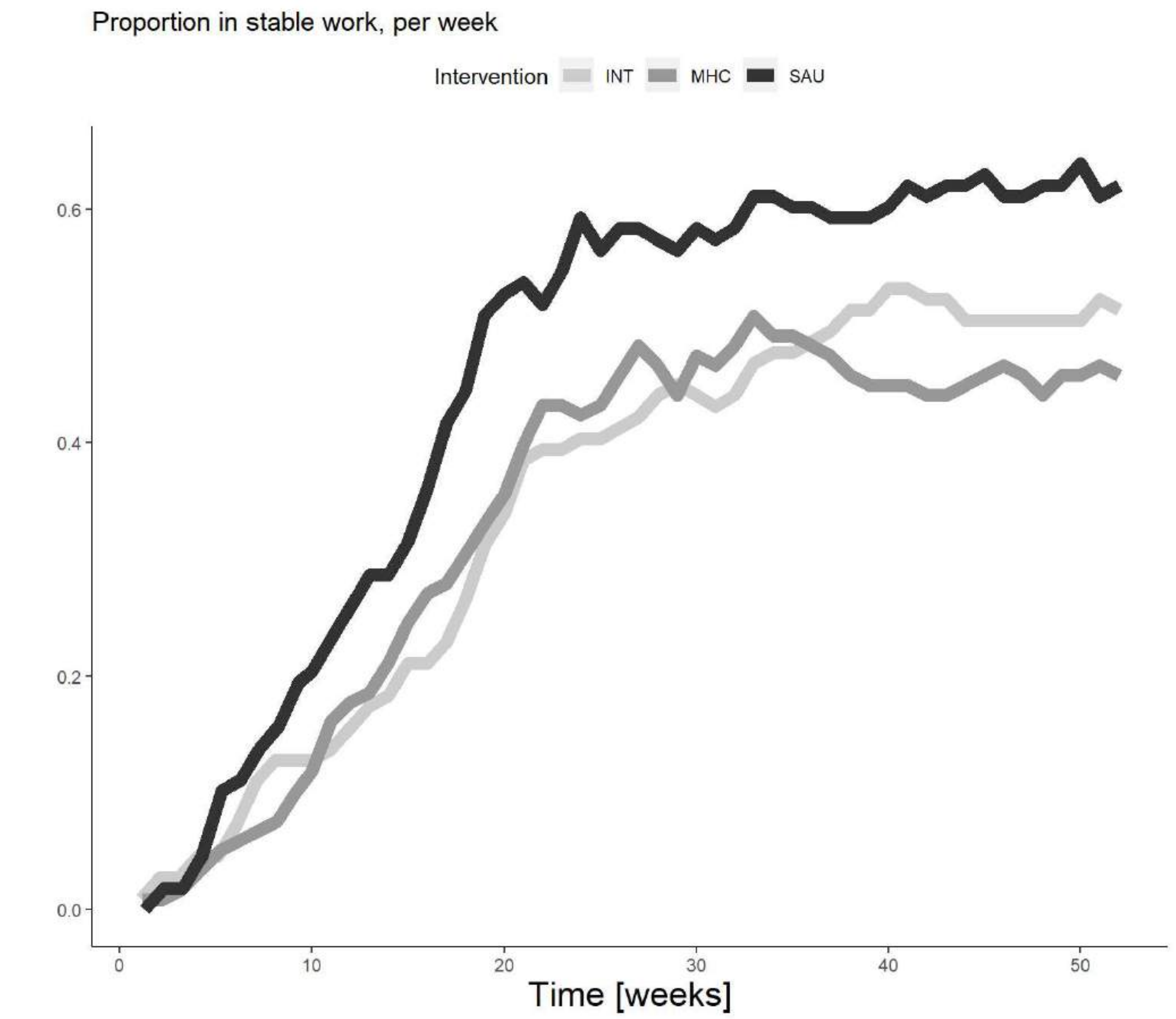

Vocational outcomes at 12-month follow-up from subgroup >ex< in RCT2

	Group values			SAU-MHC					SAU-INT					MHC-INT				
	**INT**	**MHC**	**SAU**	**Est.**	**p**	**low CI**	**:**	**high CI**	**Est.**	**p**	**low CI**	**:**	**high CI**	**Est.**	**p**	**low CI**	**:**	**high CI**
RTW, FU12 [weeks, HR]	28.0	26.0	19.0	1.46	*0.0168	1.00		2.12	1.47	*0.0174	0.99		2.18	1.03	0.88	0.69		1.52
RTW, FU6 [HR]				1.65	**0.0042	1.08		2.52	1.69	**0.0041	1.09		2.62	1.00	0.99	0.63		1.59
PROP, FU12 [%, OR]	51.9	46.6	62.0	1.95	*0.0186	0.98		3.85	1.47	0.19	0.73		2.96	0.74	0.29	0.37		1.46
WEEKS, FU12 [weeks, RR]	18.4	18.2	23.9	1.31	**0.0068	1.03		1.66	1.25	*0.0333	0.97		1.61	0.95	0.62	0.72		1.24

Self-reported outcomes at 6- and 12-month follow-up regarding: ex

	Group values						SAU-MHC				SAU-INT				MHC-INT			
	**INT**	**(SD)**	**MHC**	**(SD)**	**SAU**	**(SD)**	**Est.**	**p**	**low CI**	**high CI**	**Est.**	**p**	**low CI**	**high CI**	**Est.**	**p**	**low CI**	**high CI**
BAI [FU6]	10.1	(7.41)	8.82	(7.25)	11.44	(8.88)	2.21	*0.0378	-0.34	4.75	1.31	0.25	-1.4	4.01	-1	0.34	-3.54	1.53
BDI [FU6]	11.69	(8.77)	10.38	(8.9)	11.11	(8.43)	0.47	0.69	-2.38	3.32	-0.44	0.71	-3.3	2.42	-0.95	0.42	-3.79	1.89
PSS [FU6]	16.39	(7.26)	14.31	(6.87)	16.36	(6.83)	1.59	0.07	-0.49	3.66	-0.04	0.96	-2.19	2.1	-1.65	0.06	-3.74	0.43
KES [FU6]	63.7	(17.11)	62.03	(16.44)	68.08	(17.77)	4.87	**0.0163	0.02	9.71	2.95	0.16	-2.1	8	-2.07	0.31	-6.97	2.84
DSQ-som. [FU6]	8.46	(6.05)	8.21	(5.64)	9.84	(7.09)	1.31	0.11	-0.64	3.27	0.97	0.27	-1.16	3.1	-0.38	0.64	-2.32	1.56

	Group values						SAU-MHC				SAU-INT				MHC-INT			
	**INT**	**(SD)**	**MHC**	**(SD)**	**SAU**	**(SD)**	**Est.**	**p**	**low CI**	**high CI**	**Est.**	**p**	**low CI**	**high CI**	**Est.**	**p**	**low CI**	**high CI**
DSQ-distr. [FU6]	11.18	(7.5)	10.89	(7.1)	12.53	(7.88)	1.37	0.14	-0.87	3.61	0.96	0.31	-1.32	3.23	-0.4	0.66	-2.61	1.8
DSQ-anx. [FU6]	2.09	(3.22)	2.17	(3.62)	2.94	(4.36)	0.79	0.15	-0.53	2.11	0.66	0.21	-0.62	1.94	-0.17	0.74	-1.38	1.05
DSQ-depr. [FU6]	1.33	(2.54)	1.22	(2.24)	1.25	(2.01)	0.05	0.87	-0.69	0.8	-0.07	0.83	-0.82	0.68	-0.11	0.72	-0.88	0.65
WSAS [FU6]	13.99	(9.93)	12.78	(9.63)	13.51	(9.83)	0.3	0.81	-2.69	3.3	-0.75	0.55	-3.78	2.29	-1.05	0.39	-4.01	1.91
SPS [FU6]	19.53	(2.32)	19.62	(2.35)	19.75	(2.57)	0.14	0.65	-0.61	0.89	0.12	0.69	-0.6	0.84	-0.02	0.93	-0.69	0.64
IPQ [FU6]	17.72	(3.28)	17.12	(3.7)	17.62	(3.23)	0.47	0.35	-0.72	1.65	-0.07	0.88	-1.28	1.13	-0.56	0.26	-1.74	0.63
GSS [FU6]	28.93	(7.12)	28.85	(6.5)	28.73	(6.39)	-0.12	0.89	-2.16	1.93	0.09	0.93	-2.17	2.34	0.18	0.84	-1.95	2.31
RTW-SE [FU6]	24.84	(9.71)	25.83	(9.19)	25.73	(9.07)	0.15	0.9	-2.65	2.95	0.95	0.42	-1.88	3.77	0.8	0.5	-2.01	3.6
QoLs [FU6]	73.51	(14.13)	73.31	(14.84)	73.29	(15.15)	0.22	0.9	-4.02	4.45	0.06	0.98	-4.24	4.36	-0.18	0.92	-4.36	4
EQ5 [FU6]	0.81	(0.14)	0.83	(0.13)	0.81	(0.14)	-0.02	0.33	-0.06	0.03	0	0.78	-0.04	0.05	0.02	0.23	-0.02	0.06
IS [FU6]	25.55	(5.24)	25.22	(4.73)	21.9	(5.98)	-2.67	**0.001	-4.6	-0.74	-2.85	**7e-04	-4.85	-0.85	-0.25	0.74	-2.04	1.54
BAI [FU12]	8.89	(7.03)	9.09	(6.94)	11.21	(9.74)	1.89	0.08	-0.69	4.47	1.85	0.1	-0.85	4.54	-0.13	0.9	-2.59	2.34
BDI [FU12]	9.62	(7.45)	10.52	(9.42)	9.44	(8.28)	-0.88	0.46	-3.75	1.99	-0.24	0.84	-3.03	2.55	0.59	0.61	-2.24	3.42
PSS [FU12]	13.98	(7.1)	14.94	(7.55)	15.34	(6.68)	0.37	0.68	-1.81	2.55	1.12	0.24	-1.15	3.38	0.72	0.44	-1.52	2.96
KES [FU12]	56	(17.69)	56.32	(17.41)	59.94	(17.61)	3.53	0.1	-1.61	8.66	3.44	0.12	-1.86	8.74	-0.25	0.91	-5.42	4.92
DSQ-som. [FU12]	7.81	(5.82)	8.38	(5.94)	9.39	(6.97)	0.87	0.3	-1.14	2.88	1.16	0.19	-0.95	3.27	0.26	0.75	-1.67	2.18
DSQ-distr. [FU12]	10.38	(6.76)	11.84	(7.51)	11.38	(7.31)	-0.07	0.94	-2.33	2.2	1.04	0.27	-1.22	3.31	1.11	0.24	-1.16	3.37
DSQ-anx. [FU12]	1.54	(2.55)	2.32	(3.61)	2.88	(4.2)	0.62	0.24	-0.66	1.91	1.05	*0.039	-0.17	2.27	0.39	0.41	-0.75	1.54
DSQ-depr. [FU12]	1	(1.84)	1.58	(2.77)	0.81	(1.7)	-0.56	0.07	-1.3	0.19	-0.07	0.8	-0.77	0.62	0.48	0.12	-0.27	1.24
WSAS [FU12]	11.87	(9.62)	12.09	(9.32)	11.13	(9.67)	-0.78	0.53	-3.76	2.21	-0.84	0.51	-3.94	2.26	-0.05	0.97	-3.07	2.96
SPS [FU12]	19.37	(3.18)	18.5	(3.33)	19.8	(3.56)	0.02	0.96	-0.92	0.96	-0.37	0.32	-1.26	0.52	-0.37	0.29	-1.22	0.47
IPQ [FU12]	17.69	(3.73)	17.24	(3.92)	17.61	(3.11)	0.44	0.37	-0.75	1.63	-0.1	0.84	-1.33	1.13	-0.56	0.27	-1.76	0.65
GSS [FU12]	29.08	(7.1)	29.37	(6.48)	30.02	(6.45)	0.51	0.55	-1.52	2.54	0.83	0.37	-1.39	3.05	0.31	0.73	-1.84	2.46
RTW-SE [FU12]	27.53	(9.79)	27.91	(9.41)	28.08	(8.48)	0.2	0.87	-2.64	3.03	0.62	0.61	-2.26	3.5	0.42	0.73	-2.51	3.34
QoLs [FU12]	74.97	(13.79)	72.93	(14.97)	75.28	(13.47)	1.98	0.28	-2.37	6.32	0.43	0.81	-3.88	4.74	-1.57	0.38	-5.86	2.71
EQ5 [FU12]	0.82	(0.15)	0.82	(0.14)	0.82	(0.12)	-0.01	0.69	-0.05	0.04	0	0.82	-0.05	0.04	0	0.92	-0.04	0.05

Analyses regarding: >stress<

Kaplan Meier-curve at 12-month follow-up regarding: stress

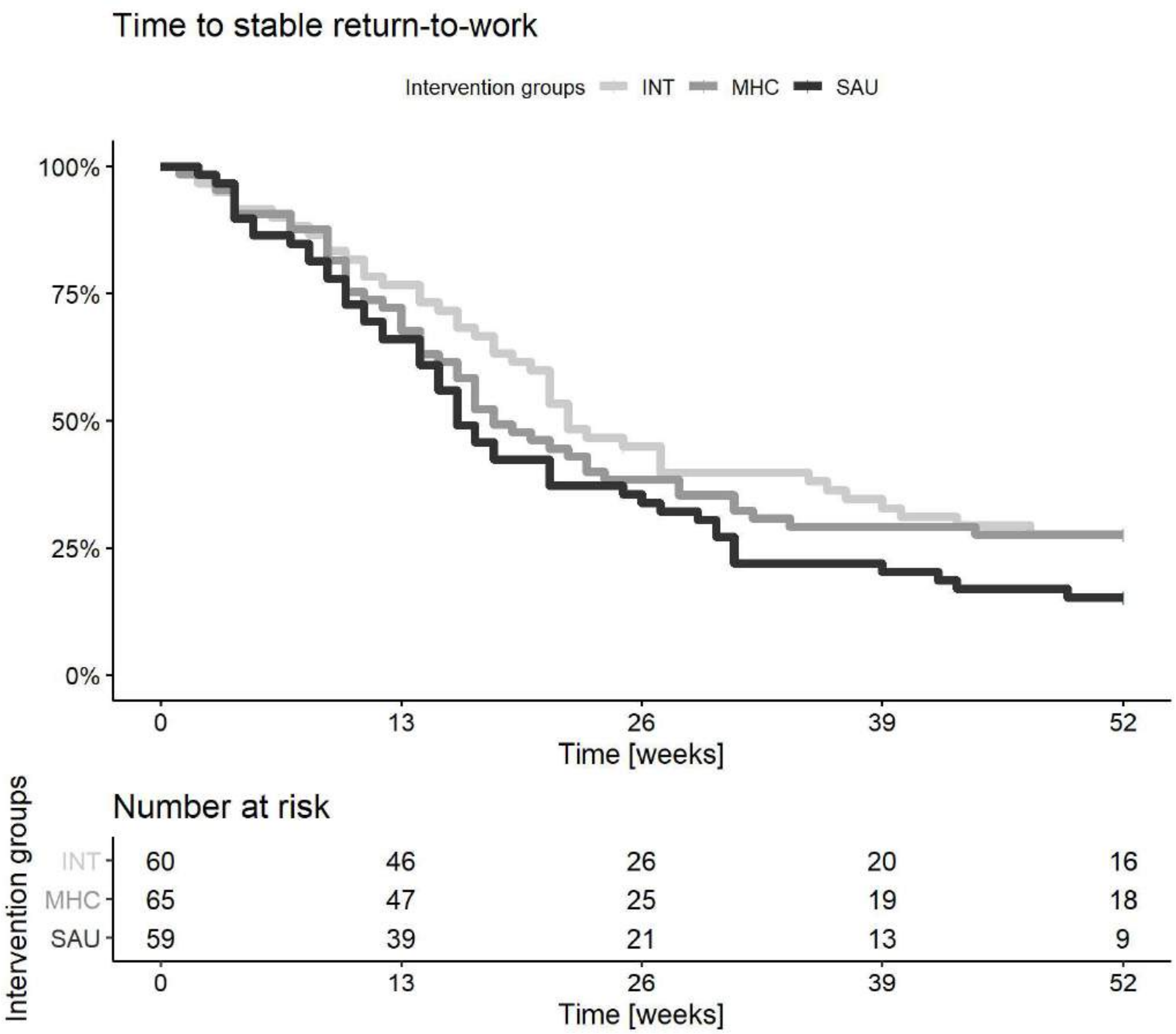

Proportion over time-curve at 12-month follow-up regarding: stress<

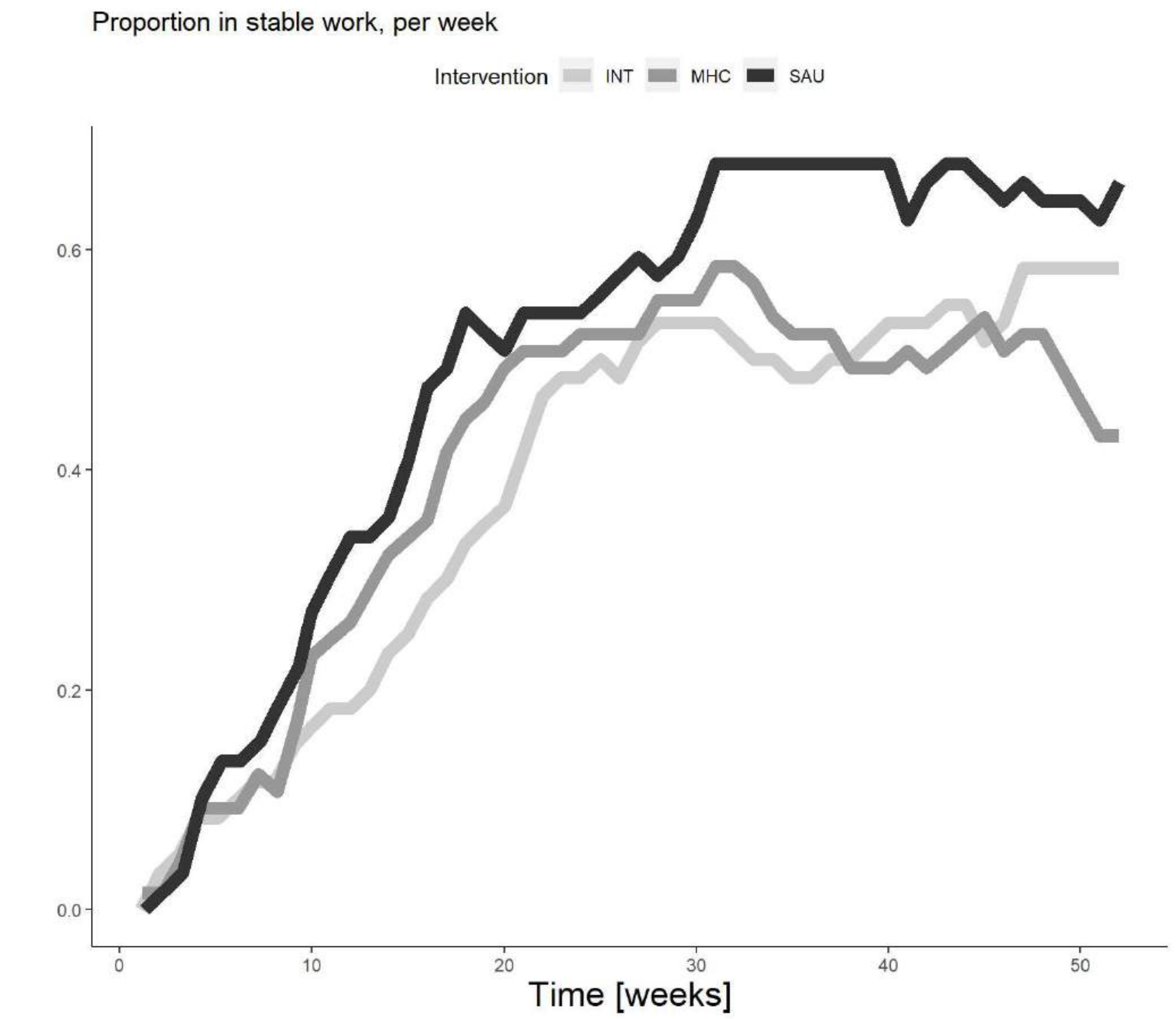

Vocational outcomes at 12-month follow-up from subgroup >stress< in RCT2

	Group values			SAU-MHC				SAU-INT				MHC-INT			
	INT	MHC	SAU	Est.	p	low CI :	high CI	Est.	p	low CI :	high CI	Est.	p	low CI :	high CI
RTW, FU12 [weeks, HR]	22.0	18.0	16.0	1.31	0.19	0.79	2.15	1.43	0.09	0.86	2.39	1.07	0.75	0.64	1.79
RTW, FU6 [HR]				1.17	0.5	0.67	2.02	1.45	0.12	0.81	2.59	1.20	0.44	0.68	2.13
PROP, FU12 [%, OR]	59.3	43.7	66.1	2.50	**0.0149	1.00	6.24	1.36	0.43	0.52	3.57	0.49	0.06	0.19	1.24
WEEKS, FU12 [weeks, RR]	20.8	21.4	26.0	1.21	0.14	0.89	1.64	1.24	0.09	0.91	1.68	1.01	0.92	0.73	1.41

Self-reported outcomes at 6- and 12-month follow-up regarding: stress

	Group values						SAU-MHC				SAU-INT				MHC-INT			
	INT	(SD)	MHC	(SD)	SAU	(SD)	Est.	p	low CI	high CI	Est.	p	low CI	high CI	Est.	p	low CI	high CI
BAI [FU6]	8.76	(7.76)	7.73	(7.41)	10.81	(7.07)	2.56	0.06	-0.7	5.82	1.8	0.21	-1.64	5.23	-0.78	0.57	-4.04	2.49
BDI [FU6]	8.49	(8.1)	8.61	(7.25)	12.65	(9.85)	3.47	*0.0265	-0.28	7.21	3.73	*0.0224	-0.18	7.64	0.32	0.82	-3.06	3.7
PSS [FU6]	14.47	(6.7)	13.36	(6.96)	16.68	(7.23)	3.06	**0.0118	0.15	5.97	2.28	0.06	-0.58	5.14	-0.76	0.49	-3.42	1.9

	Group values						SAU-MHC				SAU-INT				MHC-INT			
	**INT**	**(SD)**	**MHC**	**(SD)**	**SAU**	**(SD)**	**Est.**	**p**	**low CI**	**high CI**	**Est.**	**p**	**low CI**	**high CI**	**Est.**	**p**	**low CI**	**high CI**
KES [FU6]	56.61	(16.25)	55.91	(16.9)	63.74	(16.93)	6.87	**0.0147	0.13	13.6	6.95	**0.0119	0.34	13.56	0.12	0.96	-6.08	6.33
DSQ-som. [FU6]	6.78	(6.22)	7	(5.77)	8.62	(7.35)	1.14	0.32	-1.65	3.93	1.63	0.18	-1.27	4.52	0.47	0.66	-2.09	3.03
DSQ-distr. [FU6]	9.33	(6.66)	9.6	(6.49)	12.37	(7.98)	2.32	0.06	-0.58	5.23	2.57	*0.0398	-0.43	5.56	0.27	0.81	-2.41	2.96
DSQ-anx. [FU6]	2.21	(4.24)	1.91	(3.61)	2.91	(3.27)	0.81	0.21	-0.73	2.34	0.59	0.38	-1.02	2.2	-0.2	0.76	-1.78	1.38
DSQ-depr. [FU6]	1.17	(2.41)	0.89	(1.69)	1.66	(2.6)	0.57	0.14	-0.37	1.51	0.39	0.35	-0.61	1.39	-0.18	0.63	-1.06	0.7
WSAS [FU6]	8.96	(7.68)	7.6	(7.79)	13.78	(10.6)	5.25	**0.0016	1.3	9.21	4.37	**0.009	0.37	8.38	-0.9	0.52	-4.31	2.5
SPS [FU6]	19.68	(2.58)	19.26	(3.88)	20.1	(2.73)	0.14	0.65	-0.61	0.89	0.12	0.69	-0.6	0.84	-0.02	0.93	-0.69	0.64
IPQ [FU6]	17.37	(4.12)	17.44	(3.86)	16.1	(4.04)	-0.81	0.26	-2.55	0.92	-0.79	0.29	-2.58	0.99	0	1	-1.68	1.67
GSS [FU6]	28	(6.06)	29.57	(6.46)	28.36	(6.08)	-1.15	0.32	-3.92	1.63	0.51	0.64	-2.17	3.19	1.67	0.12	-0.9	4.25
RTW-SE [FU6]	28.22	(9.16)	27.84	(8.72)	26.57	(10.14)	-1.2	0.45	-5.01	2.61	-1.7	0.3	-5.6	2.21	-0.49	0.73	-3.98	3
QoLs [FU6]	74.98	(14)	77.49	(11.51)	74.33	(13.83)	-2.05	0.39	-7.77	3.67	-0.34	0.89	-6.37	5.68	1.68	0.45	-3.67	7.03
EQ5 [FU6]	0.86	(0.14)	0.87	(0.11)	0.83	(0.11)	-0.03	0.11	-0.08	0.02	-0.02	0.3	-0.08	0.03	0.01	0.59	-0.04	0.06
IS [FU6]	26.25	(4.45)	25.09	(4.94)	21.51	(6.03)	-2.56	*0.0176	-5.14	0.02	-3.57	**9e-04	-6.09	-1.04	-1.03	0.27	-3.32	1.26
BAI [FU12]	7.36	(6.4)	6.21	(5.67)	8.86	(7.24)	1.5	0.26	-1.69	4.69	0.66	0.64	-2.69	4	-0.85	0.53	-4.05	2.35
BDI [FU12]	8.35	(8.2)	6.47	(7.71)	7.94	(7.97)	1.47	0.35	-2.3	5.24	0.06	0.97	-3.88	4.01	-1.34	0.35	-4.82	2.13
PSS [FU12]	13.76	(7.01)	12.64	(6.71)	13.64	(6.79)	0.71	0.57	-2.3	3.73	-0.08	0.95	-3.1	2.95	-0.79	0.51	-3.63	2.06
KES [FU12]	50.54	(16.42)	46.43	(16.29)	53.28	(17.4)	5.67	*0.0489	-1.23	12.57	2.89	0.31	-4.01	9.79	-2.77	0.3	-9.26	3.72
DSQ-som. [FU12]	5.84	(5.08)	5.85	(5.76)	7.77	(6.56)	1.27	0.27	-1.49	4.04	1.21	0.3	-1.6	4.01	-0.07	0.95	-2.64	2.5
DSQ-distr. [FU12]	9.39	(6.81)	7.55	(6.7)	9.38	(6.48)	1.49	0.22	-1.43	4.41	0.32	0.8	-2.67	3.3	-1.15	0.31	-3.91	1.6
DSQ-anx. [FU12]	2.08	(3.45)	0.9	(1.6)	1.92	(3.19)	0.48	0.42	-0.95	1.91	-0.27	0.68	-1.84	1.29	-0.73	0.24	-2.24	0.78
DSQ-depr. [FU12]	1.06	(2.39)	0.6	(1.43)	1.1	(2.13)	0.21	0.57	-0.69	1.11	-0.05	0.91	-1.05	0.95	-0.26	0.49	-1.15	0.63
WSAS [FU12]	8.3	(9.06)	7.19	(8.11)	9.45	(8.8)	1.9	0.25	-2.07	5.88	0.82	0.63	-3.28	4.91	-1.08	0.47	-4.67	2.52
SPS [FU12]	19.55	(2.96)	17.91	(3.86)	18.1	(3.82)	0.02	0.96	-0.92	0.96	-0.37	0.32	-1.26	0.52	-0.37	0.29	-1.22	0.47
IPQ [FU12]	17.33	(4.4)	18.22	(4.01)	16.97	(4.5)	-0.81	0.28	-2.6	0.99	-0.1	0.9	-1.98	1.78	0.71	0.32	-1.02	2.44

	Group values						SAU-MHC				SAU-INT				MHC-INT			
	INT	(SD)	MHC	(SD)	SAU	(SD)	Est.	p	low CI	high CI	Est.	p	low CI	high CI	Est.	p	low CI	high CI
GSS [FU12]	28.81	(6.41)	31.43	(5.23)	29.77	(7.91)	-0.84	0.47	-3.63	1.96	1.42	0.23	-1.41	4.25	2.3	*0.0303	-0.24	4.85
RTW-SE [FU12]	30.18	(6.93)	29.4	(8.83)	28.71	(9.44)	-0.24	0.88	-4.18	3.7	-0.76	0.64	-4.66	3.13	-0.54	0.71	-4.08	3
QoLs [FU12]	77.67	(12.37)	78	(13.14)	79.82	(11.17)	-0.02	0.99	-5.57	5.53	1	0.68	-4.76	6.77	1.01	0.65	-4.31	6.33
EQ5 [FU12]	0.85	(0.12)	0.89	(0.11)	0.86	(0.15)	-0.03	0.15	-0.09	0.02	0	0.87	-0.05	0.06	0.04	0.09	-0.01	0.09

Analyses regarding: >interaction<

Kaplan Meier-curve at 12-month follow-up regarding: interaction

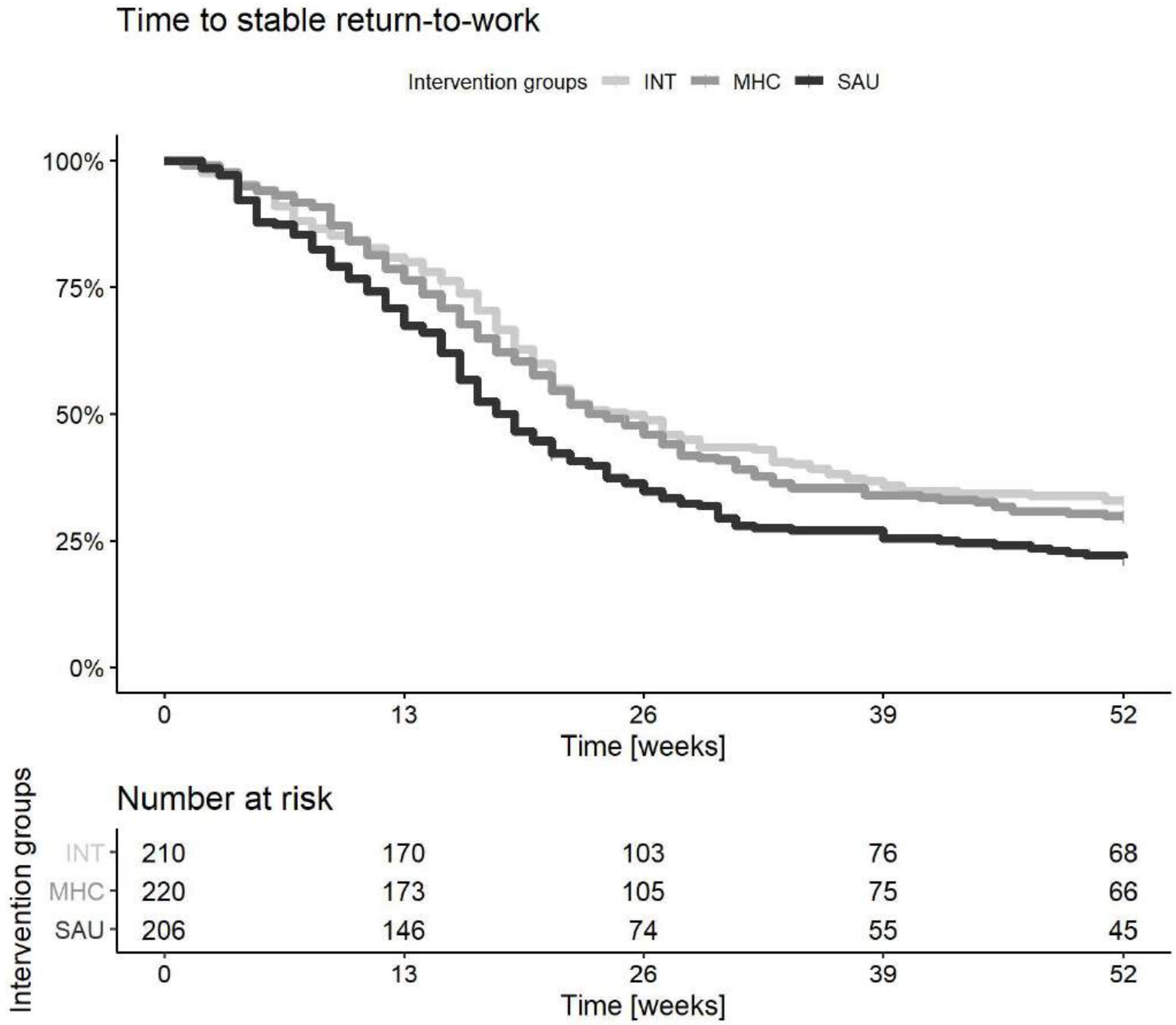

Proportion over time-curve at 12-month follow-up regarding: interaction<

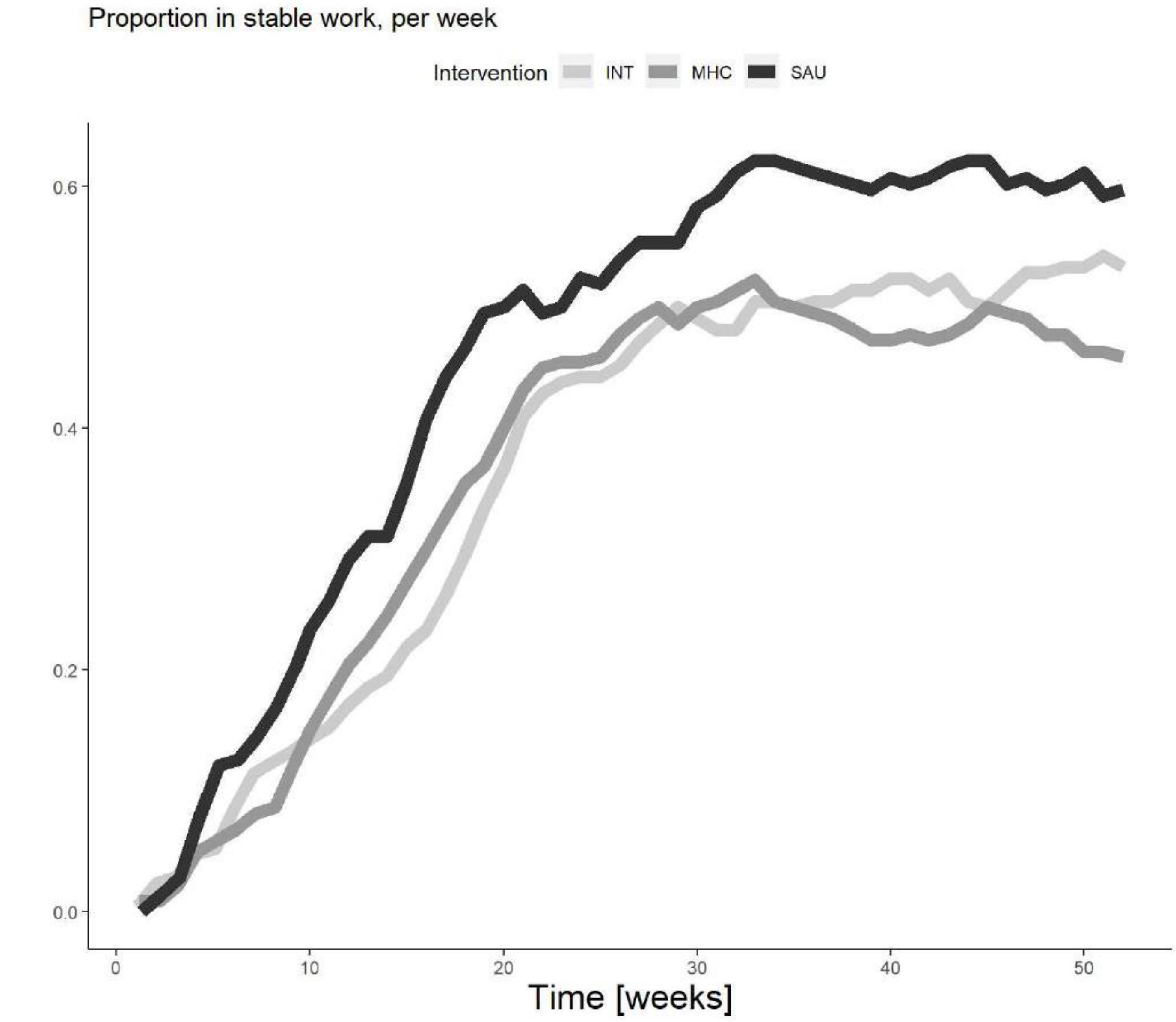

Vocational outcomes at 12-month follow-up from subgroup >interaction< in RCT2

	Group values			SAU-MHC				SAU-INT				MHC-INT			
	INT	MHC	SAU	Est.	p	low CI :	high CI	Est.	p	low CI :	high CI	Est.	p	low CI :	high CI
RTW, FU12 [weeks, HR]	25.0	23.0	19	1.17	0.56	0.61	2.27	1.36	0.26	0.71	2.63	1.16	0.58	0.60	2.26
RTW, FU12 [weeks, HR]:stratdiagex				1.22	0.54			1.07	0.83			0.89	0.70		
RTW, FU12 [weeks, HR]:stratdiagstress				1.14	0.71			1.04	0.90			0.91	0.78		
RTW, FU6 [HR]				1.24	0.5	0.58	2.65	1.26	0.45	0.61	2.60	1.03	0.93	0.49	2.17
RTW, FU6 [HR]:stratdiagex				1.32	0.45			1.33	0.42			0.99	0.98		
RTW, FU6 [HR]:stratdiagstress				0.95	0.9			1.14	0.74			1.17	0.69		
PROP, FU12 [%, OR]	53.8	46.5	60	0.74	0.52	0.23	2.31	0.82	0.67	0.26	2.54	1.17	0.74	0.37	3.70
PROP, FU12 [%, OR]:stratdiagex				0.96	0.08			0.57	0.30			-0.47	0.40		
PROP, FU12 [%, OR]:stratdiagstress				1.24	*0.0415			0.47	0.45			-0.87	0.15		
WEEKS, FU12 [weeks, RR]	19.4	19.3	24	1.07	0.72	0.69	1.64	1.09	0.61	0.72	1.66	1.04	0.83	0.67	1.61
WEEKS, FU12 [weeks, RR]:stratdiagex				0.21	0.31			0.14	0.50			-0.09	0.67		
WEEKS, FU12 [weeks, RR]:stratdiagstress				0.13	0.56			0.12	0.59			-0.03	0.91		

Self-reported outcomes at 6- and 12-month follow-up regarding: interaction

	Group values						SAU-MHC				SAU-INT				MHC-INT			
	INT	(SD)	MHC	(SD)	SAU	(SD)	Est.	p	low CI	high CI	Est.	p	low CI	high CI	Est.	p	low CI	high CI
BAI [FU6]	9.55	(7.48)	8.22	(7.07)	10.97	(7.91)	1.24	0.43	-2.52	5	1.2	0.44	-2.55	4.95	-0.19	0.9	-3.94	3.56
BDI [FU6]	10.54	(8.8)	9.36	(8.3)	11.12	(8.67)	0.44	0.8	-3.67	4.54	-1.15	0.48	-5.03	2.74	-1.73	0.3	-5.77	2.3
PSS [FU6]	15.55	(7.09)	13.77	(6.88)	16.01	(6.94)	0.94	0.47	-2.17	4.05	-0.66	0.6	-3.62	2.31	-1.72	0.16	-4.7	1.25
KES [FU6]	61.34	(17.15)	58.92	(16.97)	65.47	(18.05)	4.62	0.14	-2.89	12.13	-1.08	0.72	-8.28	6.12	-5.71	0.05	-12.79	1.36
DSQ-som. [FU6]	7.81	(6.15)	7.47	(5.54)	9.01	(6.97)	1.85	0.16	-1.31	5	0.74	0.58	-2.45	3.92	-1.19	0.33	-4.11	1.73
DSQ-distr. [FU6]	10.54	(7.21)	10.28	(6.99)	11.92	(7.75)	1.02	0.45	-2.23	4.27	-0.79	0.54	-3.93	2.35	-1.91	0.14	-5.02	1.19
DSQ-anx. [FU6]	2.13	(3.53)	2.03	(3.45)	2.78	(3.8)	0.38	0.63	-1.51	2.28	0.34	0.65	-1.46	2.15	-0.11	0.89	-1.95	1.73
DSQ-depr. [FU6]	1.29	(2.49)	1.08	(2.02)	1.24	(2.08)	-0.12	0.79	-1.2	0.96	-0.82	0.07	-1.88	0.25	-0.73	0.11	-1.85	0.38
WSAS [FU6]	11.84	(9.52)	10.46	(9.51)	13.23	(10)	2	0.28	-2.47	6.47	-0.14	0.94	-4.39	4.1	-2.26	0.19	-6.42	1.91
SPS [FU6]	19.64	(2.46)	19.48	(2.99)	19.91	(2.63)	0.93	0.42	-1.85	3.71	0.41	0.68	-2.01	2.83	-0.42	0.65	-2.66	1.81
IPQ [FU6]	17.56	(3.58)	17.29	(3.76)	17.14	(3.73)	-0.6	0.44	-2.45	1.25	-0.18	0.81	-1.97	1.61	0.48	0.51	-1.27	2.23
GSS [FU6]	28.42	(6.66)	29.2	(6.64)	28.88	(6.01)	0.69	0.59	-2.41	3.8	1.28	0.31	-1.78	4.35	0.71	0.59	-2.42	3.83
RTW-SE [FU6]	26.6	(9.55)	27.02	(9.08)	26.29	(9.36)	-0.93	0.58	-5	3.14	-1.07	0.51	-4.94	2.8	0.04	0.98	-3.85	3.93
QoLs [FU6]	74.21	(13.97)	74.95	(13.95)	74.7	(14.37)	1.61	0.55	-4.89	8.1	3.5	0.18	-2.69	9.69	2.04	0.43	-4.17	8.25
EQ5 [FU6]	0.83	(0.14)	0.85	(0.13)	0.82	(0.14)	0	0.96	-0.06	0.06	0.01	0.7	-0.05	0.07	0.01	0.63	-0.05	0.07
IS [FU6]	25.46	(5.17)	25.25	(4.92)	21.73	(5.85)	-3.18	*0.0322	-6.75	0.38	-1.85	0.19	-5.25	1.55	1.5	0.24	-1.57	4.58
BAI [FU12]	8.51	(7.01)	8.21	(7.01)	10.05	(8.57)	0.03	0.99	-3.66	3.72	0.71	0.65	-2.97	4.38	0.58	0.7	-3.02	4.17
BDI [FU12]	9.1	(8.04)	8.8	(9.01)	8.51	(7.87)	-1.09	0.52	-5.09	2.92	-1.96	0.2	-5.67	1.75	-0.91	0.58	-4.87	3.04
PSS [FU12]	13.84	(7.21)	14.02	(7.5)	14.59	(6.51)	-0.4	0.76	-3.51	2.72	-0.32	0.8	-3.32	2.67	0	1	-3.08	3.09
KES [FU12]	53.69	(17.35)	52.18	(17.83)	56.48	(17.48)	1.98	0.52	-5.48	9.44	-1.44	0.64	-8.72	5.84	-3.54	0.24	-10.83	3.75
DSQ-som. [FU12]	7.29	(5.67)	7.32	(6.01)	8.56	(6.65)	1.14	0.37	-1.89	4.17	0.47	0.71	-2.58	3.52	-0.73	0.54	-3.59	2.12
DSQ-distr. [FU12]	9.97	(6.83)	10.04	(7.57)	10.5	(6.82)	0.1	0.94	-3.06	3.26	-1.2	0.35	-4.26	1.86	-1.34	0.3	-4.45	1.77
DSQ-anx. [FU12]	1.8	(2.96)	1.75	(3.09)	2.35	(3.66)	0.08	0.91	-1.69	1.85	0.06	0.93	-1.62	1.73	-0.07	0.92	-1.77	1.62
DSQ-depr. [FU12]	1.06	(2.19)	1.21	(2.47)	0.85	(1.77)	-0.56	0.19	-1.58	0.47	-0.82	*0.0468	-1.82	0.17	-0.27	0.54	-1.35	0.81
WSAS [FU12]	10.14	(9.45)	10	(9.47)	10.09	(9.39)	-0.26	0.88	-4.52	4	-0.88	0.61	-5.02	3.27	-0.73	0.67	-4.87	3.4
SPS [FU12]	19.33	(3.17)	18.4	(3.46)	19.06	(3.76)	0.28	0.84	-3.04	3.59	-0.53	0.66	-3.45	2.39	-0.73	0.51	-3.34	1.89
IPQ [FU12]	17.67	(3.94)	17.59	(4.14)	17.44	(3.51)	-0.4	0.6	-2.22	1.42	-0.34	0.65	-2.12	1.44	0.1	0.89	-1.67	1.87
GSS [FU12]	29.25	(6.81)	30.29	(6.27)	30.09	(6.68)	0.87	0.5	-2.22	3.97	1.32	0.3	-1.74	4.37	0.51	0.69	-2.56	3.58
RTW-SE [FU12]	28.86	(9)	29.01	(9.17)	28.44	(8.67)	-0.73	0.65	-4.62	3.15	-0.87	0.59	-4.69	2.96	-0.01	0.99	-3.9	3.87
QoLs [FU12]	76.44	(13.52)	75.25	(14.87)	77.19	(12.64)	2.68	0.31	-3.66	9.01	2.44	0.34	-3.63	8.52	-0.19	0.94	-6.46	6.07
EQ5 [FU12]	0.84	(0.13)	0.85	(0.14)	0.84	(0.13)	0.01	0.68	-0.05	0.07	0.01	0.74	-0.05	0.07	0	0.95	-0.06	0.06

Analyses regarding: >emp<

Kaplan Meier-curve at 12-month follow-up regarding: emp

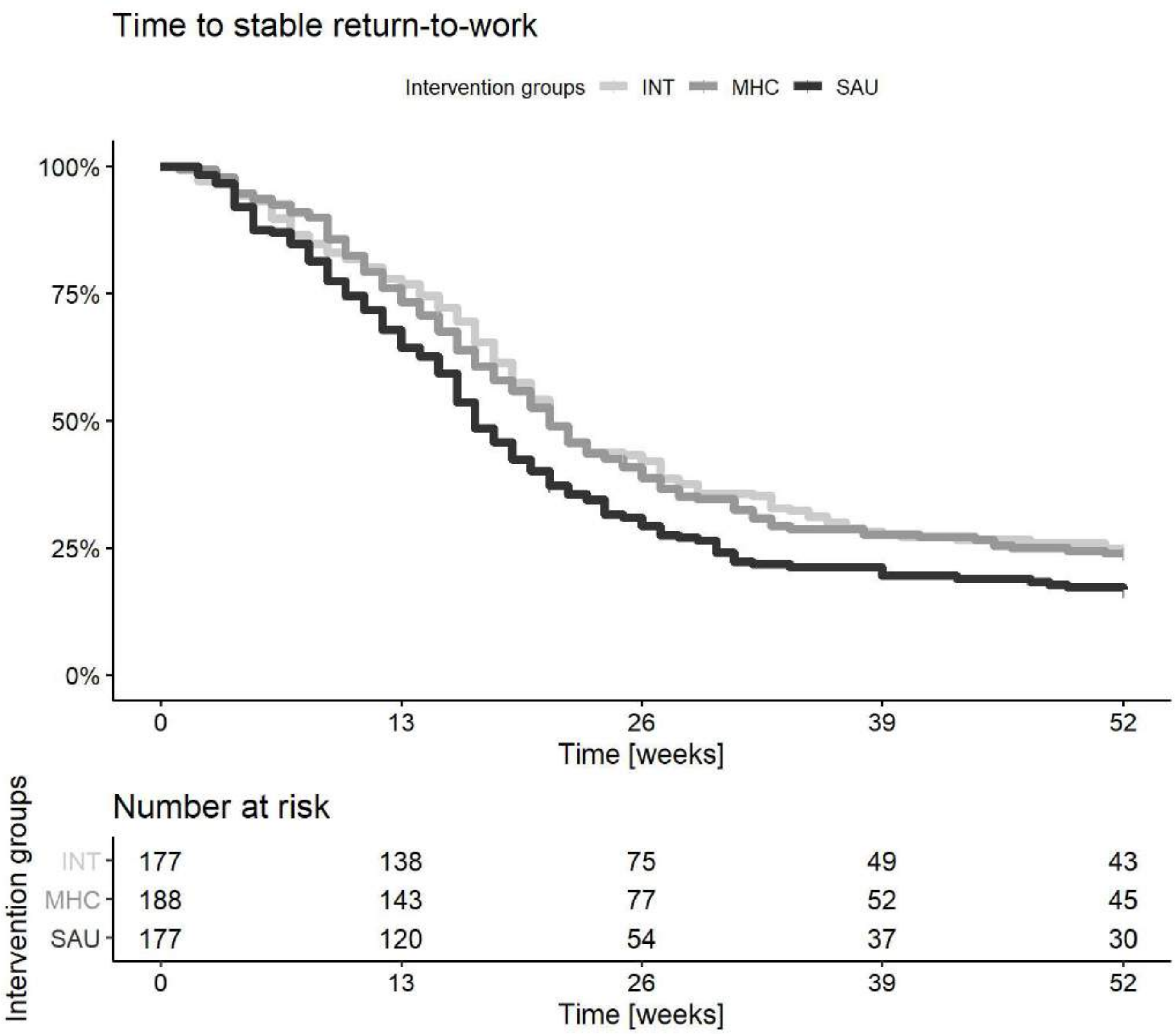

Proportion over time-curve at 12-month follow-up regarding: emp<

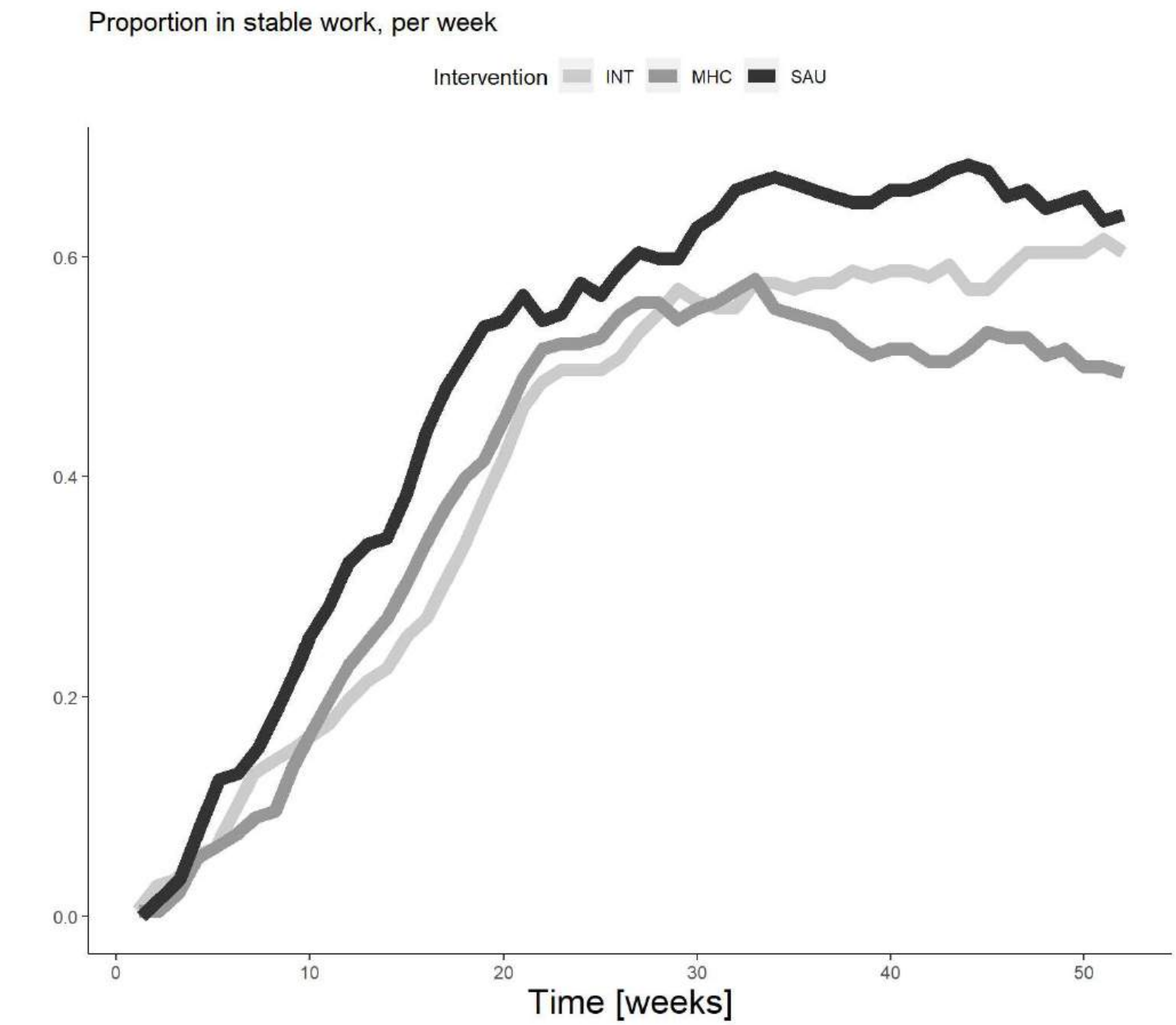

Vocational outcomes at 12-month follow-up from subgroup >emp< in RCT2

	Group values			SAU-MHC				SAU-INT				MHC-INT			
	**INT**	**MHC**	**SAU**	**Est.**	**p**	**low CI :**	**high CI**	**Est.**	**p**	**low CI :**	**high CI**	**Est.**	**p**	**low CI :**	**high CI**
RTW, FU12 [weeks, HR]	21.0	21.0	17.0	1.33	**0.0148	1.00	1.77	1.38	**0.0078	1.03	1.84	1.04	0.77	0.77	1.39
RTW, FU6 [HR]				1.37	**0.0158	1.00	1.87	1.48	**0.0034	1.07	2.04	1.08	0.56	0.78	1.50
PROP, FU12 [%, OR]	61.1	50.3	64.2	1.78	**0.0077	1.06	3.00	1.12	0.61	0.65	1.92	0.63	*0.0362	0.38	1.07
WEEKS, FU12 [weeks, RR]	22.1	21.2	26.0	1.22	**0.0058	1.03	1.45	1.17	*0.0271	0.99	1.40	0.97	0.65	0.80	1.17

Self-reported outcomes at 6- and 12-month follow-up regarding: emp

	Group values						SAU-MHC				SAU-INT				MHC-INT			
	**INT**	**(SD)**	**MHC**	**(SD)**	**SAU**	**(SD)**	**Est.**	**p**	**low CI**	**high CI**	**Est.**	**p**	**low CI**	**high CI**	**Est.**	**p**	**low CI**	**high CI**
BAI [FU6]	8.89	(6.9)	8.07	(6.89)	10.93	(8.03)	2.25	**0.0065	0.27	4.24	1.71	*0.0425	-0.31	3.72	-0.57	0.47	-2.47	1.34
BDI [FU6]	9.76	(8.08)	8.98	(7.99)	11.08	(8.29)	1.67	0.07	-0.55	3.88	1.15	0.21	-1.04	3.34	-0.53	0.54	-2.64	1.57
PSS [FU6]	14.83	(6.67)	13.41	(6.49)	15.91	(6.94)	2.14	**0.0025	0.45	3.83	0.98	0.16	-0.68	2.65	-1.16	0.08	-2.75	0.43
KES [FU6]	60.25	(16.86)	58.38	(16.33)	64.59	(17.75)	4.89	**0.003	0.95	8.82	3.23	*0.047	-0.66	7.12	-1.6	0.3	-5.33	2.13
DSQ-som. [FU6]	7.23	(5.71)	7.46	(5.35)	8.9	(7.03)	1.12	0.09	-0.47	2.71	1.4	*0.0392	-0.23	3.03	0.29	0.64	-1.18	1.75

	Group values						SAU-MHC				SAU-INT				MHC-INT			
	**INT**	**(SD)**	**MHC**	**(SD)**	**SAU**	**(SD)**	**Est.**	**p**	**low CI**	**high CI**	**Est.**	**p**	**low CI**	**high CI**	**Est.**	**p**	**low CI**	**high CI**
DSQ-distr. [FU6]	10.01	(6.76)	10.03	(6.7)	11.8	(7.66)	1.52	*0.0331	-0.19	3.23	1.49	*0.036	-0.21	3.2	-0.01	0.98	-1.63	1.6
DSQ-anx. [FU6]	2.02	(3.47)	2.03	(3.3)	2.85	(3.97)	0.76	0.05	-0.19	1.72	0.71	0.08	-0.25	1.67	-0.06	0.87	-0.98	0.86
DSQ-depr. [FU6]	1.08	(2.19)	1.03	(2.04)	1.19	(1.97)	0.15	0.51	-0.4	0.71	0.09	0.69	-0.47	0.65	-0.06	0.81	-0.62	0.5
WSAS [FU6]	11.42	(9.47)	10.32	(9.66)	12.75	(9.73)	2	*0.0451	-0.39	4.38	1.07	0.27	-1.26	3.4	-0.94	0.31	-3.16	1.29
SPS [FU6]	19.6	(2.44)	19.53	(3.06)	19.8	(2.71)	0.11	0.72	-0.64	0.87	0.12	0.68	-0.6	0.85	0	1	-0.67	0.67
IPQ [FU6]	17.72	(3.55)	17.26	(3.67)	17.28	(3.66)	0.01	0.97	-0.95	0.98	-0.42	0.3	-1.39	0.55	-0.43	0.27	-1.37	0.5
GSS [FU6]	28.77	(6.44)	29.5	(6.53)	29.05	(5.72)	-0.44	0.51	-2.06	1.18	0.23	0.73	-1.41	1.87	0.66	0.33	-0.96	2.28
RTW-SE [FU6]	27.14	(9.71)	27.35	(8.9)	26.73	(9.5)	-0.51	0.58	-2.72	1.7	-0.41	0.65	-2.63	1.8	0.14	0.88	-1.99	2.26
QoLs [FU6]	75.05	(13.59)	75.78	(13.59)	75.24	(14.11)	-0.37	0.79	-3.71	2.98	0.2	0.89	-3.18	3.59	0.52	0.7	-2.73	3.77
EQ5 [FU6]	0.84	(0.14)	0.85	(0.13)	0.82	(0.13)	-0.02	0.2	-0.05	0.02	-0.01	0.45	-0.04	0.02	0.01	0.62	-0.03	0.04
IS [FU6]	25.86	(5.05)	25.52	(4.59)	21.73	(5.92)	-2.9	**0	-4.44	-1.37	-3.24	**0	-4.8	-1.69	-0.34	0.55	-1.71	1.02
BAI [FU12]	8.27	(7.12)	7.96	(6.87)	9.78	(8.67)	1.36	0.09	-0.58	3.29	1.19	0.15	-0.81	3.19	-0.17	0.82	-2.03	1.69
BDI [FU12]	8.66	(7.77)	8.35	(8.62)	8.28	(6.99)	0.08	0.93	-2.12	2.27	-0.19	0.83	-2.32	1.94	-0.28	0.75	-2.4	1.84
PSS [FU12]	13.62	(7.05)	13.61	(7.18)	14.27	(6.23)	0.52	0.47	-1.2	2.23	0.56	0.43	-1.15	2.27	0.03	0.96	-1.66	1.73
KES [FU12]	53.19	(17.66)	51.73	(17.47)	56.08	(17.05)	3.53	*0.0331	-0.44	7.49	2.17	0.2	-1.85	6.2	-1.29	0.43	-5.22	2.64
DSQ-som. [FU12]	7.28	(5.85)	7.32	(5.97)	8.44	(6.76)	0.82	0.22	-0.77	2.41	0.85	0.21	-0.77	2.47	0.04	0.95	-1.45	1.53
DSQ-distr. [FU12]	9.79	(6.8)	9.86	(7.18)	10.37	(6.58)	0.57	0.43	-1.14	2.27	0.5	0.48	-1.2	2.2	-0.06	0.93	-1.73	1.61
DSQ-anx. [FU12]	1.78	(2.92)	1.82	(3.22)	2.36	(3.67)	0.5	0.19	-0.41	1.41	0.46	0.23	-0.46	1.38	-0.04	0.91	-0.92	0.84
DSQ-depr. [FU12]	0.97	(2.06)	1.16	(2.45)	0.73	(1.45)	-0.34	0.14	-0.88	0.21	-0.15	0.48	-0.68	0.37	0.18	0.43	-0.37	0.74
WSAS [FU12]	9.68	(9.35)	9.37	(8.86)	10.17	(9.4)	0.7	0.48	-1.7	3.1	0.33	0.74	-2.06	2.71	-0.4	0.67	-2.68	1.87
SPS [FU12]	19.23	(3.18)	18.44	(3.49)	18.97	(3.75)	0.01	0.98	-0.92	0.95	-0.31	0.4	-1.21	0.58	-0.31	0.38	-1.15	0.53
IPQ [FU12]	17.62	(3.95)	17.79	(3.99)	17.68	(3.48)	-0.11	0.78	-1.1	0.87	-0.11	0.79	-1.13	0.91	0.02	0.97	-0.97	1
GSS [FU12]	29.44	(6.76)	30.54	(6.11)	30.58	(6.37)	0.11	0.87	-1.5	1.72	1.08	0.12	-0.57	2.74	0.97	0.16	-0.67	2.61
RTW-SE [FU12]	29.05	(9.11)	29.31	(9.2)	29.09	(8.6)	-0.2	0.83	-2.44	2.05	0.26	0.79	-2.02	2.54	0.5	0.59	-1.71	2.72
QoLs [FU12]	77.1	(13.59)	75.57	(14.45)	77.6	(12.42)	1.12	0.42	-2.2	4.45	0.48	0.73	-2.84	3.81	-0.67	0.62	-3.92	2.59
EQ5 [FU12]	0.84	(0.14)	0.85	(0.14)	0.84	(0.12)	-0.01	0.49	-0.04	0.02	0	0.95	-0.03	0.03	0.01	0.56	-0.03	0.04

Analyses regarding: >vac<

Kaplan Meier-curve at 12-month follow-up regarding: vac

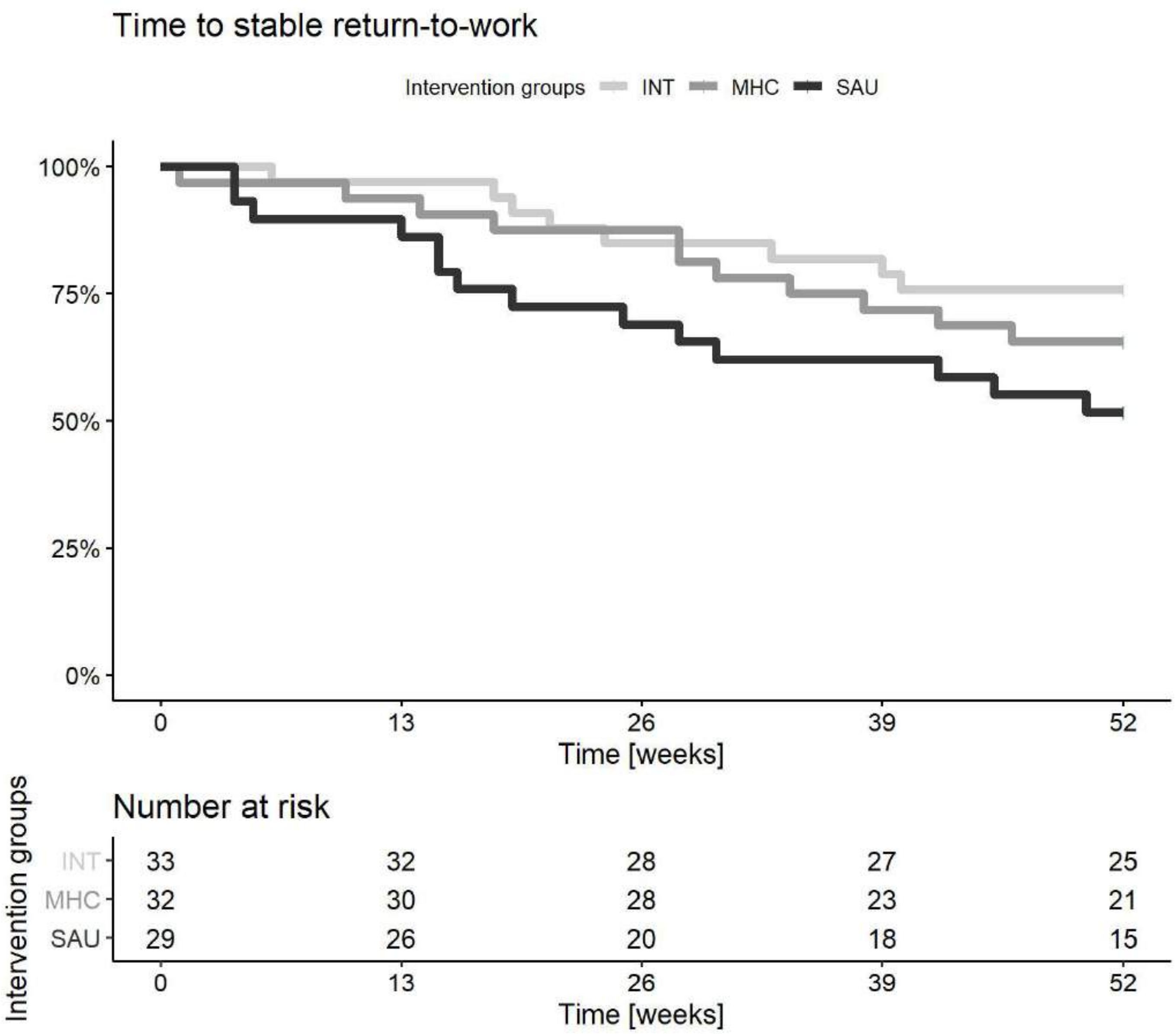

Proportion over time-curve at 12-month follow-up regarding: vac<

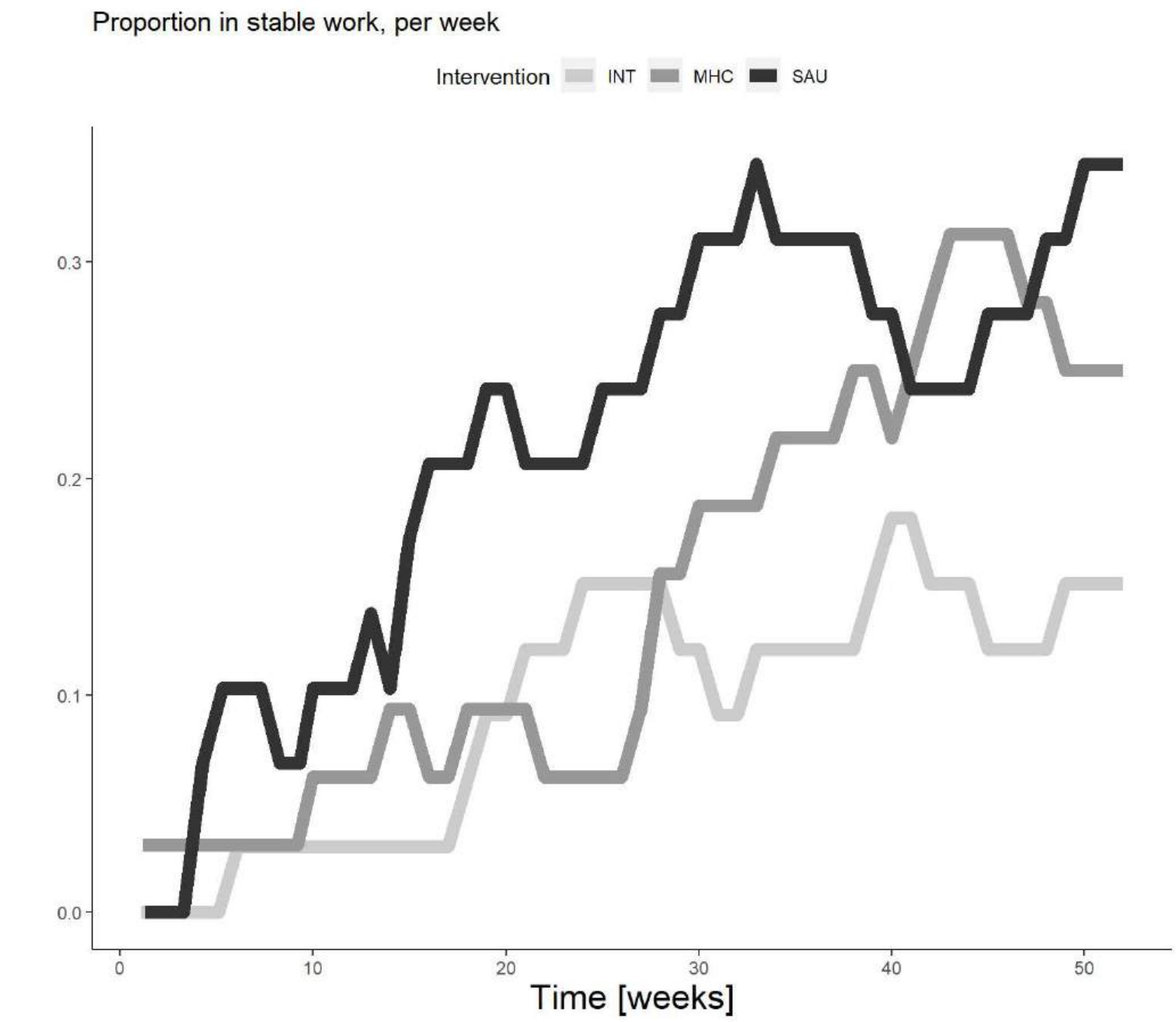

Vocational outcomes at 12-month follow-up from subgroup >vac< in RCT2

	Group values			SAU-MHC					SAU-INT					MHC-INT				
	INT	MHC	SAU	Est.	p	low CI	:	high CI	Est.	p	low CI	:	high CI	Est.	p	low CI	:	high CI
RTW, FU12 [weeks, HR]				1.79	0.16	0.65		4.93	3.05	**0.0157	0.98		9.48	1.58	0.35	0.48		5.2
RTW, FU6 [HR]				3.12	0.06	0.70		13.83	2.74	0.08	0.68		11.07	0.85	0.80	0.16		4.5
PROP, FU12 [%, OR]	15.2	25.0	34.5	1.70	0.37	0.40		7.28	3.79	*0.0469	0.73		19.75	1.93	0.31	0.39		9.7
WEEKS, FU12 [weeks, RR]	5.2	7.8	11.6	1.58	0.20	0.66		3.78	2.37	*0.0398	0.84		6.66	1.49	0.35	0.52		4.3

Self-reported outcomes at 6- and 12-month follow-up regarding: vac

	Group values						SAU-MHC				SAU-INT				MHC-INT			
	INT	(SD)	MHC	(SD)	SAU	(SD)	Est.	p	low CI	high CI	Est.	p	low CI	high CI	Est.	p	low CI	high CI
BAI [FU6]	13.29	(9.48)	9.19	(8.15)	11.21	(7.31)	1.52	0.45	-3.37	6.41	-1.51	0.5	-6.88	3.87	-3.28	0.14	-8.63	2.07
BDI [FU6]	14.97	(11.28)	11.68	(9.88)	11.37	(10.88)	-0.61	0.8	-6.44	5.23	-3.13	0.21	-9.18	2.93	-2.73	0.27	-8.75	3.29
PSS [FU6]	19.64	(8.09)	16	(8.7)	16.61	(7.01)	-0.07	0.97	-4.3	4.17	-2.64	0.14	-6.98	1.71	-2.74	0.12	-7.05	1.57
KES [FU6]	68.04	(17.76)	62.08	(20.35)	70.77	(19.36)	7.29	0.08	-2.81	17.39	2.22	0.59	-7.9	12.35	-5.47	0.19	-15.47	4.53
DSQ-som. [FU6]	11.23	(7.59)	7.54	(6.72)	9.73	(6.67)	2.24	0.2	-2	6.47	-1.58	0.4	-6.14	2.98	-3.63	*0.0333	-7.72	0.47

	Group values						SAU-MHC				SAU-INT				MHC-INT			
	INT	(SD)	MHC	(SD)	SAU	(SD)	Est.	p	low CI	high CI	Est.	p	low CI	high CI	Est.	p	low CI	high CI
DSQ-distr. [FU6]	13.62	(8.97)	11.77	(8.46)	12.68	(8.37)	0.63	0.74	-3.95	5.21	-1.5	0.44	-6.25	3.25	-2.26	0.24	-6.91	2.39
DSQ-anx. [FU6]	2.81	(3.86)	2.04	(4.32)	2.32	(2.51)	0.26	0.8	-2.23	2.76	-0.58	0.54	-2.92	1.75	-0.98	0.31	-3.33	1.37
DSQ-depr. [FU6]	2.58	(3.61)	1.38	(1.88)	1.55	(2.7)	0.01	0.99	-1.49	1.5	-1.02	0.16	-2.75	0.71	-1.06	0.12	-2.71	0.58
WSAS [FU6]	14.33	(9.6)	11.31	(8.74)	16.09	(11.27)	2.7	0.29	-3.44	8.83	0.86	0.74	-5.49	7.21	-1.91	0.42	-7.66	3.84
SPS [FU6]	20	(2.83)	18.71	(1.6)	20.75	(1.76)	0.11	0.72	-0.64	0.87	0.12	0.68	-0.6	0.85	0	1	-0.67	0.67
IPQ [FU6]	16.6	(3.74)	17.5	(4.36)	16.24	(4.11)	-0.71	0.5	-3.25	1.83	0.37	0.72	-2.12	2.86	0.83	0.4	-1.56	3.21
GSS [FU6]	26.21	(7.66)	27.38	(7.13)	27.91	(7.57)	0.58	0.73	-3.56	4.72	1.83	0.29	-2.33	6	1.43	0.39	-2.62	5.48
RTW-SE [FU6]	23.37	(7.94)	25	(10.02)	23.65	(8.13)	-0.27	0.91	-5.88	5.34	0.83	0.69	-4.29	5.94	1.26	0.58	-4.29	6.81
QoLs [FU6]	68.83	(15.47)	70.16	(15.28)	71.2	(15.88)	1.64	0.65	-7.07	10.34	2.42	0.5	-6.19	11.04	0.69	0.85	-7.86	9.24
EQ5 [FU6]	0.78	(0.12)	0.85	(0.11)	0.81	(0.17)	-0.02	0.58	-0.11	0.07	0.04	0.28	-0.05	0.13	0.05	0.09	-0.02	0.13
IS [FU6]	23.08	(5.39)	23.65	(6.39)	21.73	(5.53)	-1.3	0.45	-5.54	2.95	-0.24	0.88	-4.2	3.71	0.82	0.63	-3.35	4.98
BAI [FU12]	10.23	(6.08)	9.79	(7.78)	11.76	(7.92)	1.02	0.62	-4.01	6.05	0.16	0.94	-5.15	5.47	-1.15	0.59	-6.34	4.04
BDI [FU12]	12.13	(9.35)	11.65	(10.96)	9.95	(12.11)	-2.08	0.41	-8.2	4.05	-2.39	0.36	-8.7	3.91	-0.46	0.86	-6.58	5.65
PSS [FU12]	15.36	(8.27)	16.54	(9)	16.52	(7.92)	-0.5	0.79	-5.09	4.09	0.18	0.93	-4.58	4.94	0.46	0.81	-4.33	5.26
KES [FU12]	57.05	(15)	55.18	(20.26)	58.9	(20.19)	3.6	0.41	-6.97	14.16	2.77	0.52	-7.58	13.11	-1.17	0.78	-11.36	9.02
DSQ-som. [FU12]	7.36	(4.42)	7.33	(6.44)	9.3	(6.06)	1.53	0.38	-2.65	5.71	0.88	0.63	-3.52	5.29	-0.46	0.79	-4.5	3.59
DSQ-distr. [FU12]	11.18	(7.06)	11.23	(9.96)	11.3	(8.32)	-0.36	0.86	-5.12	4.41	-0.05	0.98	-4.75	4.64	0.12	0.95	-4.68	4.92
DSQ-anx. [FU12]	1.95	(3.34)	1.24	(1.84)	2.35	(3.7)	0.25	0.8	-2.11	2.61	-0.02	0.98	-2.28	2.24	-0.35	0.71	-2.65	1.94
DSQ-depr. [FU12]	1.73	(2.93)	1.55	(2.61)	1.6	(3.03)	-0.24	0.71	-1.77	1.29	-0.41	0.57	-2.14	1.32	-0.21	0.76	-1.86	1.45
WSAS [FU12]	13.32	(9.8)	13.92	(12.13)	9.62	(9.52)	-3.95	0.13	-10.19	2.28	-3.03	0.25	-9.39	3.33	0.63	0.8	-5.54	6.8
SPS [FU12]	20.15	(3.08)	18	(3.22)	20	(3.94)	0.01	0.98	-0.92	0.95	-0.31	0.4	-1.21	0.58	-0.31	0.38	-1.15	0.53
IPQ [FU12]	18	(3.96)	16.19	(4.92)	16.05	(3.44)	0.51	0.63	-2.1	3.13	-0.79	0.46	-3.38	1.79	-1.51	0.16	-4.08	1.06
GSS [FU12]	27.95	(7.19)	28.64	(7.16)	26.89	(7.94)	-0.04	0.98	-4.31	4.23	0.06	0.97	-4.2	4.31	0.17	0.92	-3.84	4.19
RTW-SE [FU12]	27.59	(8.31)	27	(8.86)	24.5	(8.27)	0.06	0.98	-5.79	5.92	-1.78	0.43	-7.26	3.69	-1.78	0.45	-7.49	3.94
QoLs [FU12]	71.95	(12.44)	73.05	(17.69)	74.75	(13.98)	2.39	0.5	-6.19	10.97	1.65	0.64	-6.78	10.08	-0.87	0.81	-9.43	7.69
EQ5 [FU12]	0.79	(0.1)	0.82	(0.14)	0.8	(0.17)	-0.01	0.81	-0.1	0.08	0.01	0.79	-0.08	0.1	0.02	0.62	-0.06	0.1

Analyses regarding: >intempl<

Kaplan Meier-curve at 12-month follow-up regarding: intempl

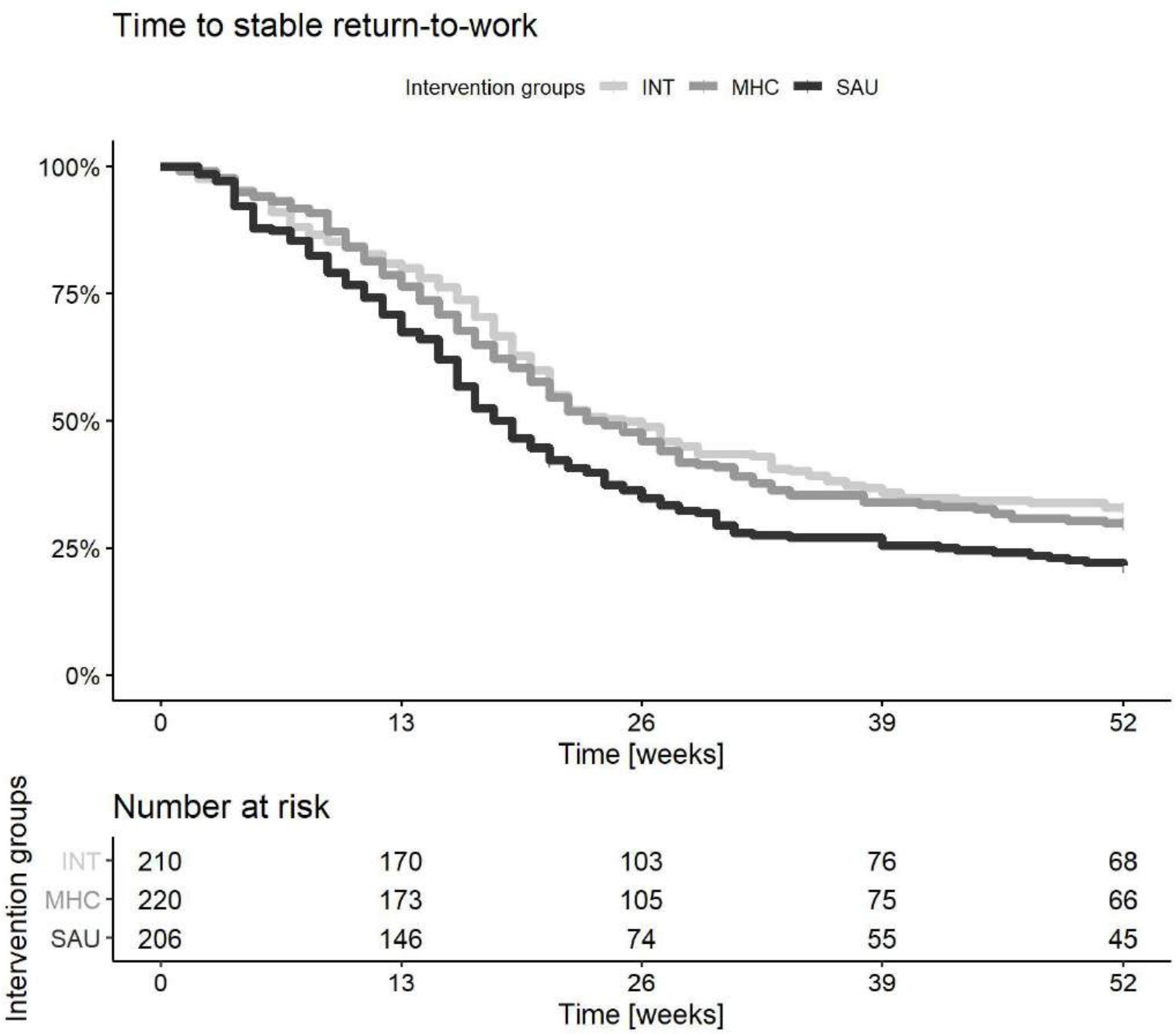

Proportion over time-curve at 12-month follow-up regarding: intempl<

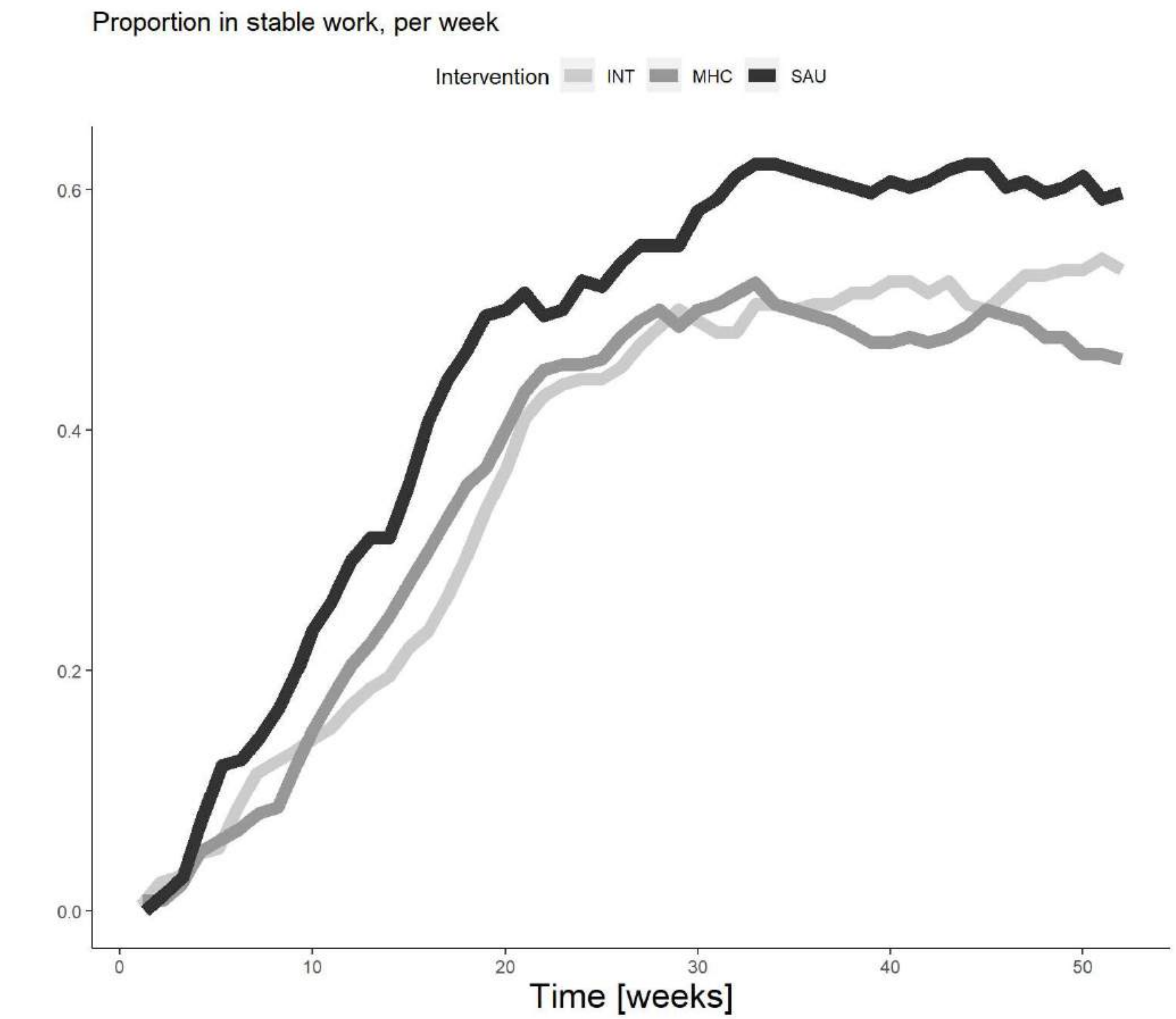

Vocational outcomes at 12-month follow-up from subgroup >intempl< in RCT2

	Group values			SAU-MHC					SAU-INT					MHC-INT				
	**INT**	**MHC**	**SAU**	**Est.**	**p**	**low CI**	**:**	**high CI**	**Est.**	**p**	**low CI**	**:**	**high CI**	**Est.**	**p**	**low CI**	**:**	**high CI**
RTW, FU12 [weeks, HR]	25.0	23.0	19	1.33	**0.0148	1.00		1.77	1.37	**0.0082	1.03		1.84	1.04	0.75	0.78		1.39
RTW, FU12 [weeks, HR]:stratlabormarketvac				1.19	0.67				1.76	0.22				1.41	0.47			
RTW, FU6 [HR]				1.36	*0.018	1.00		1.86	1.47	**0.004	1.07		2.03	1.09	0.54	0.78		1.51
RTW, FU6 [HR]:stratlabormarketvac				2.02	0.25				1.59	0.42				0.76	0.69			
PROP, FU12 [%, OR]	53.8	46.5	60	1.78	**0.0076	1.06		2.99	1.12	0.62	0.65		1.91	0.64	*0.0384	0.38		1.07
PROP, FU12 [%, OR]:stratlabormarketvac				-0.11	0.86				1.00	0.13				1.05	0.12			
WEEKS, FU12 [weeks, RR]	19.4	19.3	24	1.22	**0.0055	1.03		1.45	1.17	*0.0272	0.99		1.40	0.97	0.66	0.80		1.17
WEEKS, FU12 [weeks, RR]:stratlabormarketvac				0.20	0.6				0.64	0.14				0.43	0.34			

Analyses regarding: >north<

Kaplan Meier-curve at 12-month follow-up regarding: north

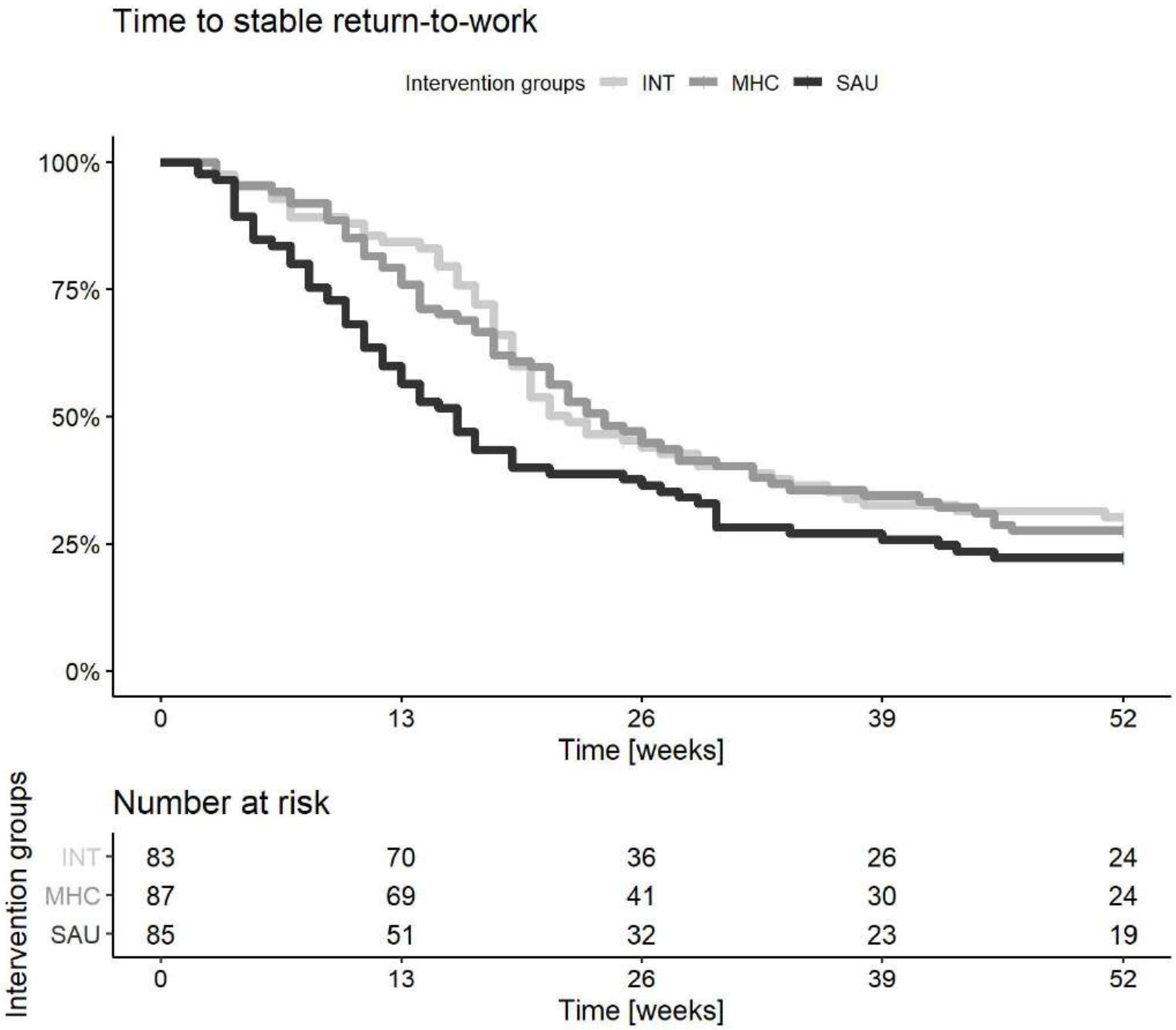

Proportion over time-curve at 12-month follow-up regarding: north<

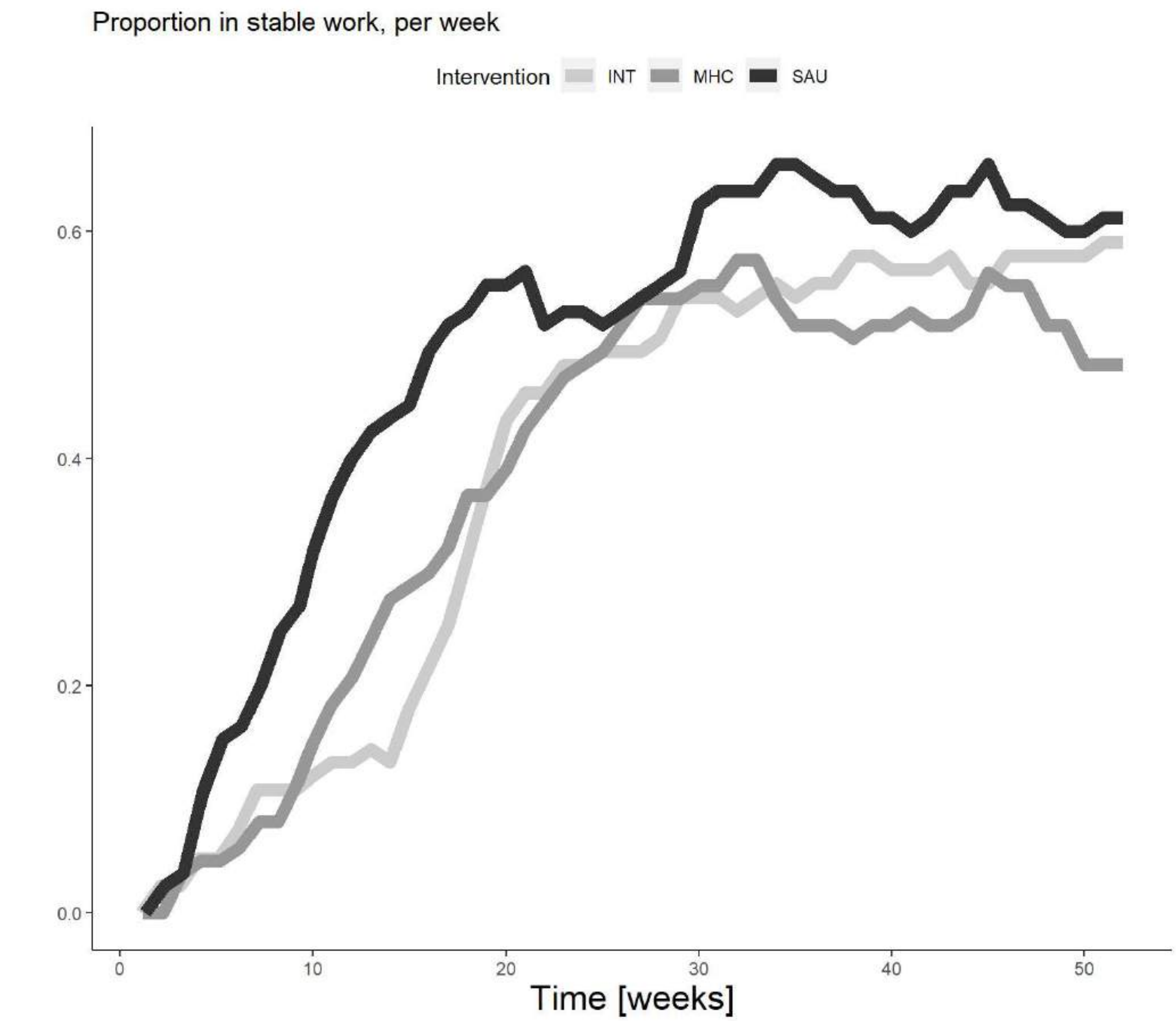

Vocational outcomes at 12-month follow-up from subgroup >north< in RCT2

	Group values			SAU-MHC				SAU-INT				MHC-INT			
	INT	MHC	SAU	Est.	p	low CI	high CI	Est.	p	low CI	high CI	Est.	p	low CI	high CI
RTW, FU12 [weeks, HR]	22.0	24.0	16.0	1.45	*0.0352	0.95	2.23	1.50	*0.0268	0.96	2.32	0.99	0.95	0.63	1.54
RTW, FU6 [HR]				1.57	*0.0237	0.97	2.54	1.57	*0.0261	0.96	2.55	0.97	0.87	0.59	1.60
PROP, FU12 [%, OR]	60.5	48.8	61.2	1.70	0.1	0.78	3.71	0.97	0.94	0.42	2.28	0.55	0.09	0.24	1.28
WEEKS, FU12 [weeks, RR]	20.9	20.4	25.7	1.26	*0.0286	0.98	1.62	1.20	0.06	0.95	1.53	0.97	0.75	0.74	1.26

Self-reported outcomes at 6- and 12-month follow-up regarding: north

	Group values						SAU-MHC				SAU-INT				MHC-INT			
	INT	(SD)	MHC	(SD)	SAU	(SD)	Est.	p	low CI	high CI	Est.	p	low CI	high CI	Est.	p	low CI	high CI
BAI [FU6]	8.32	(7.16)	8.29	(7.57)	9.48	(7.3)	0.9	0.44	-1.92	3.71	0.97	0.41	-1.87	3.81	0.14	0.91	-2.65	2.92
BDI [FU6]	10.23	(9.38)	9.77	(8.5)	10.1	(8.84)	0.2	0.88	-3.06	3.47	-0.09	0.95	-3.37	3.2	-0.17	0.9	-3.35	3
PSS [FU6]	14.51	(7.66)	14.32	(6.49)	14.81	(7.29)	0.5	0.62	-1.93	2.94	0.37	0.73	-2.16	2.89	-0.07	0.94	-2.45	2.3
KES [FU6]	59.52	(16.38)	58.51	(15.39)	63.45	(18.98)	3.96	0.09	-1.71	9.63	3.24	0.19	-2.63	9.1	-0.61	0.78	-5.81	4.59
DSQ-som. [FU6]	6.47	(5.18)	7.44	(5.32)	8.61	(7.61)	0.99	0.33	-1.43	3.41	1.76	0.09	-0.71	4.23	0.78	0.39	-1.4	2.96

	Group values						SAU-MHC				SAU-INT				MHC-INT			
	INT	(SD)	MHC	(SD)	SAU	(SD)	Est.	p	low CI	high CI	Est.	p	low CI	high CI	Est.	p	low CI	high CI
DSQ-distr. [FU6]	9.64	(6.91)	10.54	(6.09)	10.82	(7.92)	0.41	0.69	-2.07	2.89	0.94	0.37	-1.57	3.46	0.61	0.52	-1.71	2.94
DSQ-anx. [FU6]	1.26	(2.15)	2.03	(4.01)	2.16	(3.1)	0.15	0.79	-1.18	1.47	0.68	0.15	-0.44	1.81	0.56	0.26	-0.65	1.78
DSQ-depr. [FU6]	1.13	(2.39)	0.76	(1.4)	1.27	(2.27)	0.41	0.2	-0.36	1.17	0.03	0.93	-0.8	0.86	-0.35	0.27	-1.13	0.42
WSAS [FU6]	10	(8.33)	10.21	(8.47)	13.03	(10.2)	2	0.15	-1.35	5.34	2.28	0.1	-1.08	5.64	0.34	0.79	-2.74	3.42
SPS [FU6]	19.86	(1.95)	19.73	(2.54)	20.13	(2.37)	0.14	0.66	-0.62	0.9	0.16	0.59	-0.56	0.89	0.04	0.88	-0.63	0.71
IPQ [FU6]	17.82	(3.5)	17.57	(3.69)	16.5	(3.99)	-0.77	0.2	-2.23	0.68	-1	0.11	-2.48	0.49	-0.21	0.72	-1.64	1.21
GSS [FU6]	29.44	(6.32)	29.21	(6.2)	29.34	(6.09)	-0.06	0.94	-2.32	2.19	0.18	0.85	-2.18	2.55	0.21	0.82	-2.03	2.46
RTW-SE [FU6]	28	(8.58)	26.62	(8.95)	27.07	(8.79)	0.41	0.75	-2.68	3.51	-0.87	0.5	-3.96	2.22	-1.28	0.3	-4.24	1.68
QoLs [FU6]	76.21	(13.65)	74.55	(14.45)	76.06	(13.24)	1.92	0.35	-3.06	6.9	0.44	0.83	-4.47	5.35	-1.63	0.42	-6.43	3.18
EQ5 [FU6]	0.84	(0.12)	0.85	(0.11)	0.85	(0.12)	0	0.95	-0.05	0.04	0.01	0.59	-0.04	0.06	0.01	0.57	-0.03	0.06
IS [FU6]	25.71	(5.47)	24.81	(5.63)	21.72	(6.26)	-2.29	*0.0214	-4.68	0.1	-3.21	**0.001	-5.53	-0.89	-0.94	0.29	-3.11	1.22
BAI [FU12]	6.77	(5.7)	7.88	(5.43)	7.87	(6.82)	-0.28	0.81	-2.99	2.44	0.42	0.72	-2.36	3.2	0.74	0.52	-2.02	3.51
BDI [FU12]	9.2	(8.09)	8.57	(7.54)	7.74	(7.62)	-0.84	0.52	-4.02	2.34	-1.39	0.3	-4.6	1.83	-0.47	0.73	-3.69	2.75
PSS [FU12]	13.31	(7.17)	14.39	(7.21)	13.06	(6.22)	-1.13	0.28	-3.61	1.35	-0.34	0.75	-2.92	2.24	0.82	0.44	-1.73	3.37
KES [FU12]	51.07	(15.77)	51.51	(15.1)	55.08	(16.13)	3.18	0.18	-2.54	8.9	3.36	0.18	-2.61	9.33	0.28	0.9	-5.32	5.89
DSQ-som. [FU12]	5.43	(4.32)	6.85	(5.04)	7.43	(6.61)	0.54	0.59	-1.85	2.92	1.52	0.14	-0.94	3.98	1	0.26	-1.13	3.14
DSQ-distr. [FU12]	9.29	(6.4)	10.28	(6.82)	9.62	(6.2)	-0.46	0.66	-2.93	2.01	0.31	0.77	-2.18	2.8	0.82	0.42	-1.61	3.24
DSQ-anx. [FU12]	0.99	(1.58)	1.34	(2.39)	1.69	(2.79)	0.04	0.93	-1.19	1.28	0.47	0.28	-0.59	1.54	0.44	0.35	-0.71	1.6
DSQ-depr. [FU12]	0.83	(1.88)	0.84	(2)	0.92	(1.84)	-0.03	0.93	-0.79	0.73	0.07	0.84	-0.72	0.86	0.11	0.74	-0.67	0.9
WSAS [FU12]	8.91	(8.86)	9.99	(8.8)	9.27	(8.93)	-0.8	0.57	-4.17	2.58	0.09	0.95	-3.28	3.46	0.94	0.49	-2.29	4.16
SPS [FU12]	19.33	(2.79)	18.77	(3.75)	18.39	(4.45)	0.06	0.87	-0.86	0.99	-0.34	0.37	-1.23	0.56	-0.37	0.29	-1.21	0.47
IPQ [FU12]	18.29	(4.06)	17.29	(3.83)	17.5	(3.78)	0.08	0.89	-1.37	1.54	-0.65	0.31	-2.19	0.88	-0.74	0.22	-2.21	0.73
GSS [FU12]	30.39	(6.23)	29.97	(5.53)	31.6	(6.02)	1.39	0.14	-0.87	3.65	1.44	0.15	-0.95	3.83	0.04	0.97	-2.28	2.35
RTW-SE [FU12]	30.72	(8.05)	28.43	(8.95)	29.2	(9.03)	0.71	0.6	-2.51	3.92	-0.83	0.53	-4.02	2.35	-1.53	0.24	-4.66	1.59
QoLs [FU12]	78.29	(12.66)	75.31	(13.28)	78.03	(11.23)	2.35	0.25	-2.55	7.26	-0.24	0.91	-4.98	4.51	-2.67	0.18	-7.49	2.16
EQ5 [FU12]	0.85	(0.11)	0.85	(0.12)	0.83	(0.13)	-0.02	0.41	-0.06	0.03	-0.01	0.66	-0.05	0.04	0.01	0.74	-0.04	0.05

Analyses regarding: >city<

Kaplan Meier-curve at 12-month follow-up regarding: city

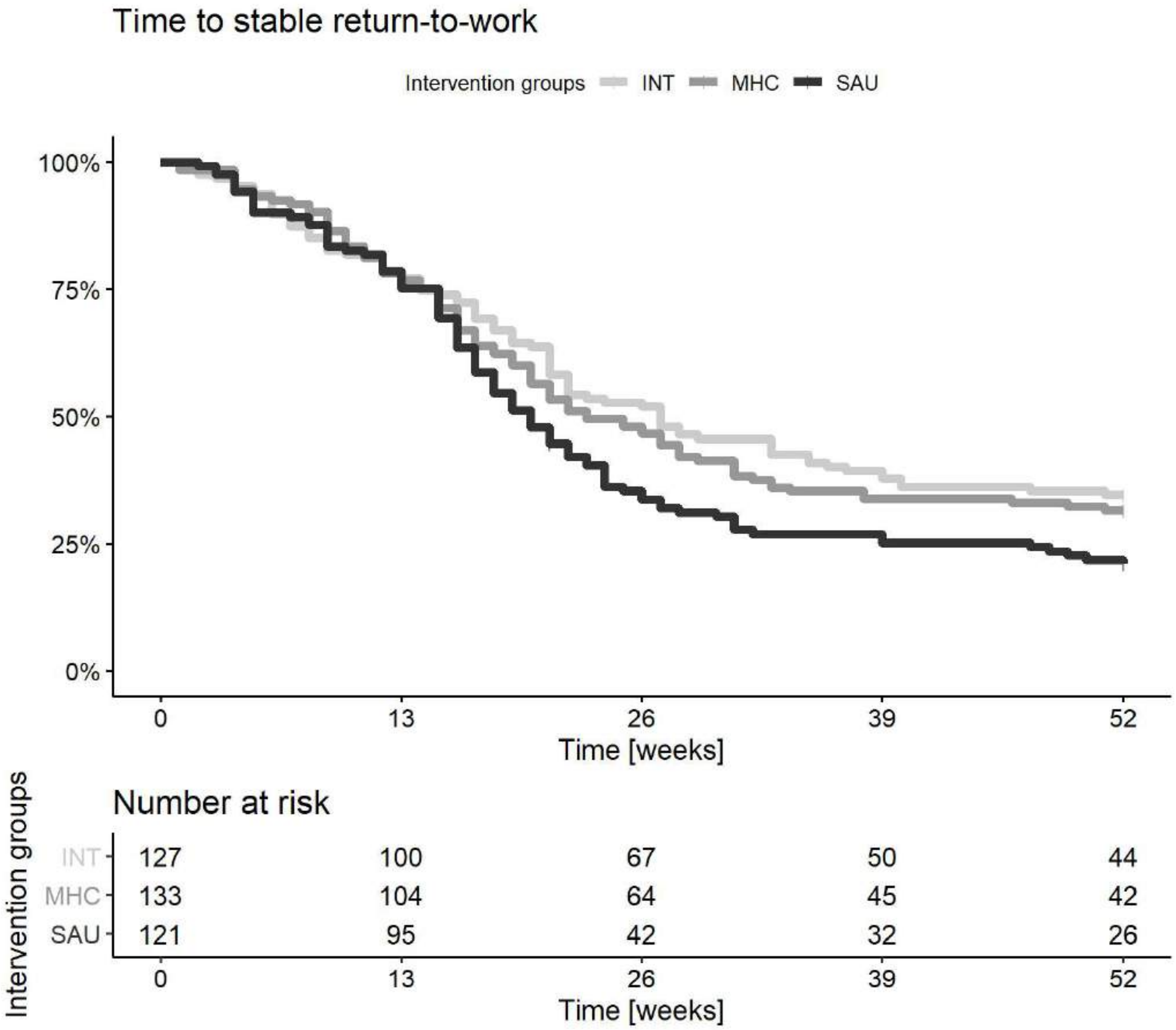

Proportion over time-curve at 12-month follow-up regarding: city<

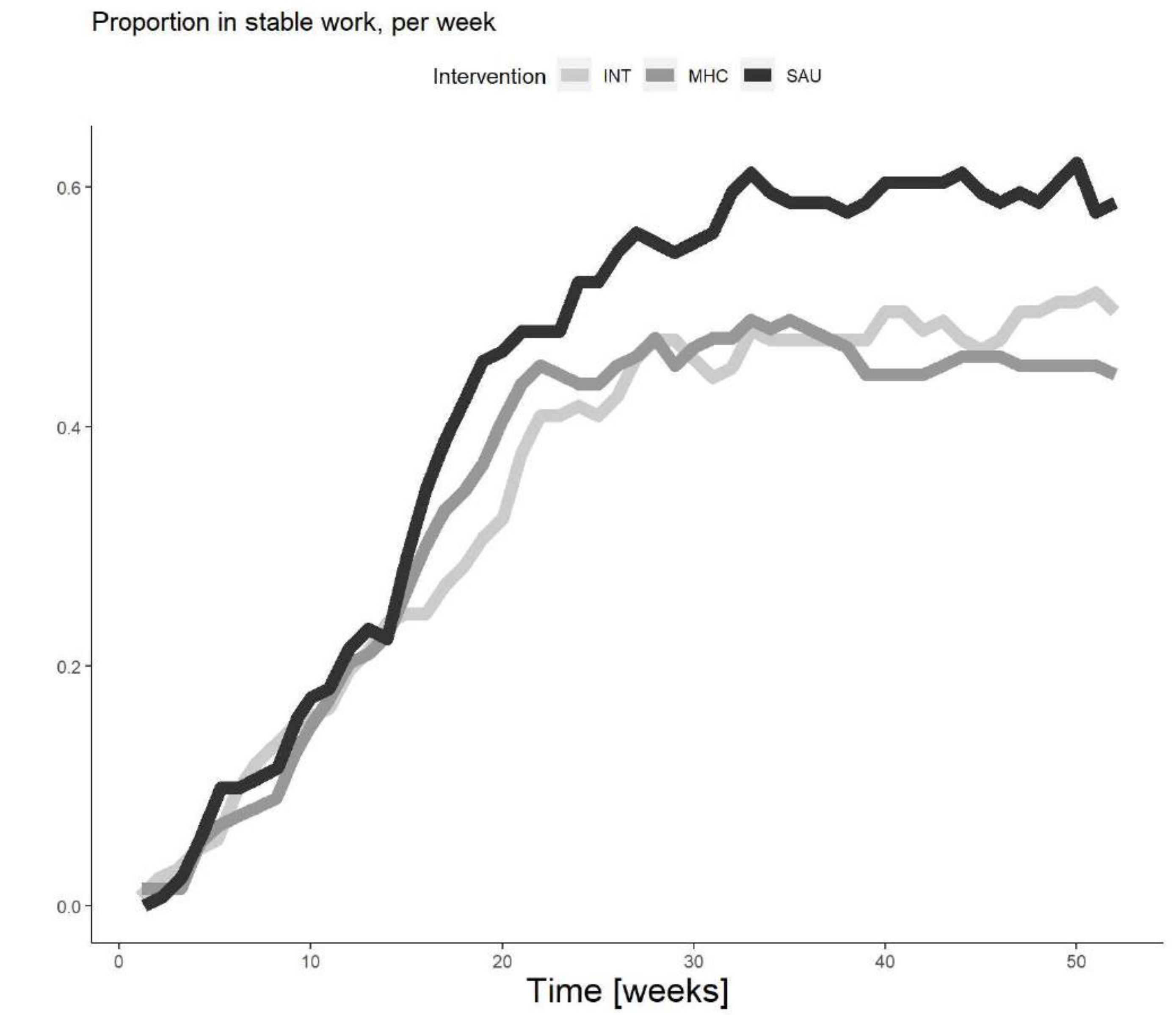

Vocational outcomes at 12-month follow-up from subgroup >city< in RCT2

	Group values			SAU-MHC				SAU-INT				MHC-INT			
	**INT**	**MHC**	**SAU**	**Est.**	**p**	**low CI :**	**high CI**	**Est.**	**p**	**low CI :**	**high CI**	**Est.**	**p**	**low CI :**	**high CI**
RTW, FU12 [weeks, HR]	27.0	23.0	20.0	1.28	0.09	0.90	1.83	1.42	*0.0198	0.99	2.05	1.10	0.52	0.76	1.59
RTW, FU6 [HR]				1.32	0.09	0.89	1.96	1.53	**0.0128	1.01	2.31	1.15	0.43	0.75	1.75
PROP, FU12 [%, OR]	49.6	45.0	59.2	1.79	*0.0249	0.96	3.33	1.46	0.15	0.78	2.76	0.82	0.43	0.45	1.51
WEEKS, FU12 [weeks, RR]	18.5	18.6	22.8	1.22	*0.0399	0.97	1.54	1.22	*0.0475	0.96	1.56	1.00	0.99	0.77	1.30

Self-reported outcomes at 6- and 12-month follow-up regarding: city

	Group values						SAU-MHC				SAU-INT				MHC-INT			
	**INT**	**(SD)**	**MHC**	**(SD)**	**SAU**	**(SD)**	**Est.**	**p**	**low CI**	**high CI**	**Est.**	**p**	**low CI**	**high CI**	**Est.**	**p**	**low CI**	**high CI**
BAI [FU6]	10.37	(7.61)	8.18	(6.76)	12.08	(8.2)	3.02	**0.0027	0.61	5.42	1.34	0.21	-1.22	3.9	-1.72	0.09	-4.12	0.68
BDI [FU6]	10.74	(8.42)	9.1	(8.2)	11.89	(8.51)	2.16	*0.05	-0.48	4.8	0.87	0.43	-1.77	3.51	-1.28	0.24	-3.9	1.33
PSS [FU6]	16.25	(6.61)	13.43	(7.11)	16.89	(6.56)	2.75	**0.0013	0.7	4.79	0.46	0.58	-1.52	2.44	-2.27	**0.0051	-4.21	-0.33

	**Group values**						**SAU-MHC**				**SAU-INT**				**MHC-INT**			
	**INT**	**(SD)**	**MHC**	**(SD)**	**SAU**	**(SD)**	**Est.**	**p**	**low CI**	**high CI**	**Est.**	**p**	**low CI**	**high CI**	**Est.**	**p**	**low CI**	**high CI**
KES [FU6]	62.61	(17.64)	59.17	(17.94)	67.03	(17.26)	6.12	**0.0022	1.35	10.88	2.85	0.14	-1.81	7.51	-3.25	0.1	-7.95	1.46
DSQ-som. [FU6]	8.76	(6.63)	7.49	(5.7)	9.31	(6.47)	1.47	0.06	-0.42	3.36	0.41	0.62	-1.58	2.4	-1.1	0.15	-2.96	0.75
DSQ-distr. [FU6]	11.18	(7.39)	10.12	(7.51)	12.74	(7.55)	2.1	*0.0168	0	4.2	1.13	0.2	-0.97	3.24	-0.96	0.26	-3.02	1.1
DSQ-anx. [FU6]	2.75	(4.14)	2.04	(3.07)	3.24	(4.21)	1.07	*0.0324	-0.13	2.27	0.42	0.43	-0.87	1.71	-0.66	0.18	-1.85	0.53
DSQ-depr. [FU6]	1.41	(2.56)	1.29	(2.31)	1.22	(1.94)	-0.04	0.9	-0.74	0.66	-0.13	0.67	-0.84	0.59	-0.08	0.78	-0.82	0.65
WSAS [FU6]	13.08	(10.09)	10.62	(10.16)	13.38	(9.9)	2.21	0.07	-0.74	5.15	0.13	0.92	-2.77	3.02	-2.09	0.07	-4.88	0.69
SPS [FU6]	19.48	(2.76)	19.31	(3.27)	19.72	(2.83)	0.14	0.66	-0.62	0.9	0.16	0.59	-0.56	0.89	0.04	0.88	-0.63	0.71
IPQ [FU6]	17.39	(3.65)	17.12	(3.81)	17.61	(3.48)	0.37	0.44	-0.78	1.51	0.19	0.7	-0.97	1.34	-0.19	0.69	-1.3	0.93
GSS [FU6]	27.73	(6.82)	29.18	(6.94)	28.54	(5.95)	-0.46	0.59	-2.49	1.57	0.82	0.33	-1.21	2.85	1.26	0.13	-0.75	3.27
RTW-SE [FU6]	25.66	(10.08)	27.27	(9.19)	25.71	(9.78)	-1.08	0.35	-3.83	1.67	0.28	0.81	-2.44	3	1.35	0.22	-1.27	3.96
QoLs [FU6]	72.82	(14.09)	75.2	(13.7)	73.72	(15.12)	-1.48	0.38	-5.5	2.55	0.61	0.72	-3.48	4.71	2.05	0.21	-1.87	5.97
EQ5 [FU6]	0.83	(0.15)	0.84	(0.14)	0.8	(0.15)	-0.03	0.09	-0.07	0.01	-0.01	0.47	-0.05	0.03	0.02	0.3	-0.02	0.06
IS [FU6]	25.29	(4.98)	25.52	(4.43)	21.73	(5.56)	-2.93	**1e-04	-4.69	-1.17	-2.57	**0.001	-4.43	-0.72	0.34	0.61	-1.28	1.96
BAI [FU12]	9.68	(7.57)	8.43	(7.89)	11.82	(9.44)	2.38	*0.0189	-0.05	4.82	1.33	0.21	-1.24	3.9	-1.05	0.29	-3.4	1.3
BDI [FU12]	9.03	(8.05)	8.95	(9.89)	9.13	(8.05)	0.2	0.86	-2.53	2.94	0	1	-2.67	2.67	-0.18	0.87	-2.81	2.45
PSS [FU12]	14.19	(7.25)	13.77	(7.71)	15.82	(6.51)	1.39	0.12	-0.75	3.53	0.99	0.26	-1.13	3.12	-0.37	0.67	-2.46	1.73
KES [FU12]	55.41	(18.18)	52.63	(19.54)	57.68	(18.58)	3.76	0.08	-1.31	8.83	1.35	0.52	-3.67	6.38	-2.34	0.26	-7.33	2.64
DSQ-som. [FU12]	8.6	(6.14)	7.63	(6.57)	9.44	(6.59)	1.19	0.15	-0.77	3.14	0.39	0.64	-1.63	2.41	-0.8	0.31	-2.68	1.09
DSQ-distr. [FU12]	10.44	(7.1)	9.88	(8.06)	11.2	(7.24)	1.05	0.24	-1.11	3.22	0.47	0.6	-1.68	2.62	-0.56	0.53	-2.7	1.59
DSQ-anx. [FU12]	2.36	(3.51)	2.02	(3.46)	2.91	(4.19)	0.75	0.12	-0.42	1.92	0.35	0.5	-0.89	1.59	-0.39	0.41	-1.54	0.75
DSQ-depr. [FU12]	1.22	(2.37)	1.45	(2.72)	0.8	(1.71)	-0.51	0.08	-1.21	0.18	-0.37	0.21	-1.06	0.33	0.16	0.61	-0.58	0.89
WSAS [FU12]	10.95	(9.79)	10.01	(9.93)	10.75	(9.74)	0.64	0.61	-2.33	3.61	-0.46	0.71	-3.43	2.52	-1.1	0.36	-3.98	1.78
SPS [FU12]	19.32	(3.48)	18.18	(3.28)	19.6	(3.04)	0.06	0.87	-0.86	0.99	-0.34	0.37	-1.23	0.56	-0.37	0.29	-1.21	0.47
IPQ [FU12]	17.25	(3.82)	17.78	(4.33)	17.39	(3.29)	-0.11	0.83	-1.3	1.09	0.1	0.85	-1.13	1.33	0.21	0.68	-1.01	1.43

	Group values						SAU-MHC				SAU-INT				MHC-INT			
	INT	(SD)	MHC	(SD)	SAU	(SD)	Est.	p	low CI	high CI	Est.	p	low CI	high CI	Est.	p	low CI	high CI
GSS [FU12]	28.49	(7.1)	30.5	(6.72)	28.85	(6.98)	-0.78	0.36	-2.82	1.26	0.74	0.4	-1.37	2.85	1.5	0.08	-0.56	3.55
RTW-SE [FU12]	27.61	(9.42)	29.38	(9.33)	27.79	(8.37)	-0.73	0.53	-3.57	2.1	0.56	0.63	-2.26	3.38	1.29	0.26	-1.46	4.03
QoLs [FU12]	75.16	(14)	75.21	(15.89)	76.51	(13.72)	0.61	0.72	-3.55	4.78	1.38	0.43	-2.83	5.6	0.72	0.67	-3.35	4.8
EQ5 [FU12]	0.83	(0.15)	0.84	(0.16)	0.84	(0.13)	-0.01	0.78	-0.05	0.04	0.01	0.66	-0.03	0.05	0.01	0.47	-0.03	0.06

Analyses regarding: >intteams<

Kaplan Meier-curve at 12-month follow-up regarding: intteams

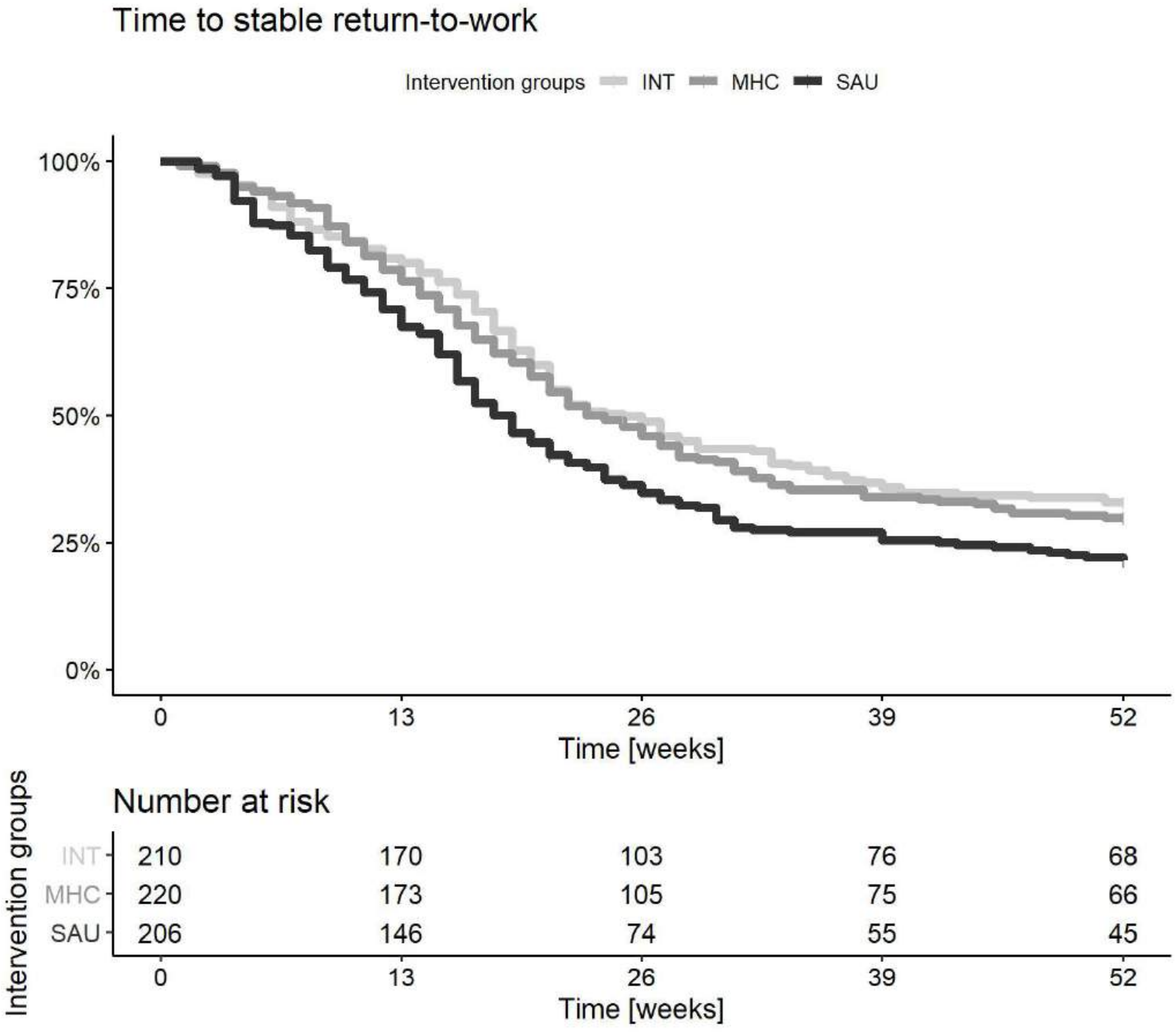

Proportion over time-curve at 12-month follow-up regarding: intteams<

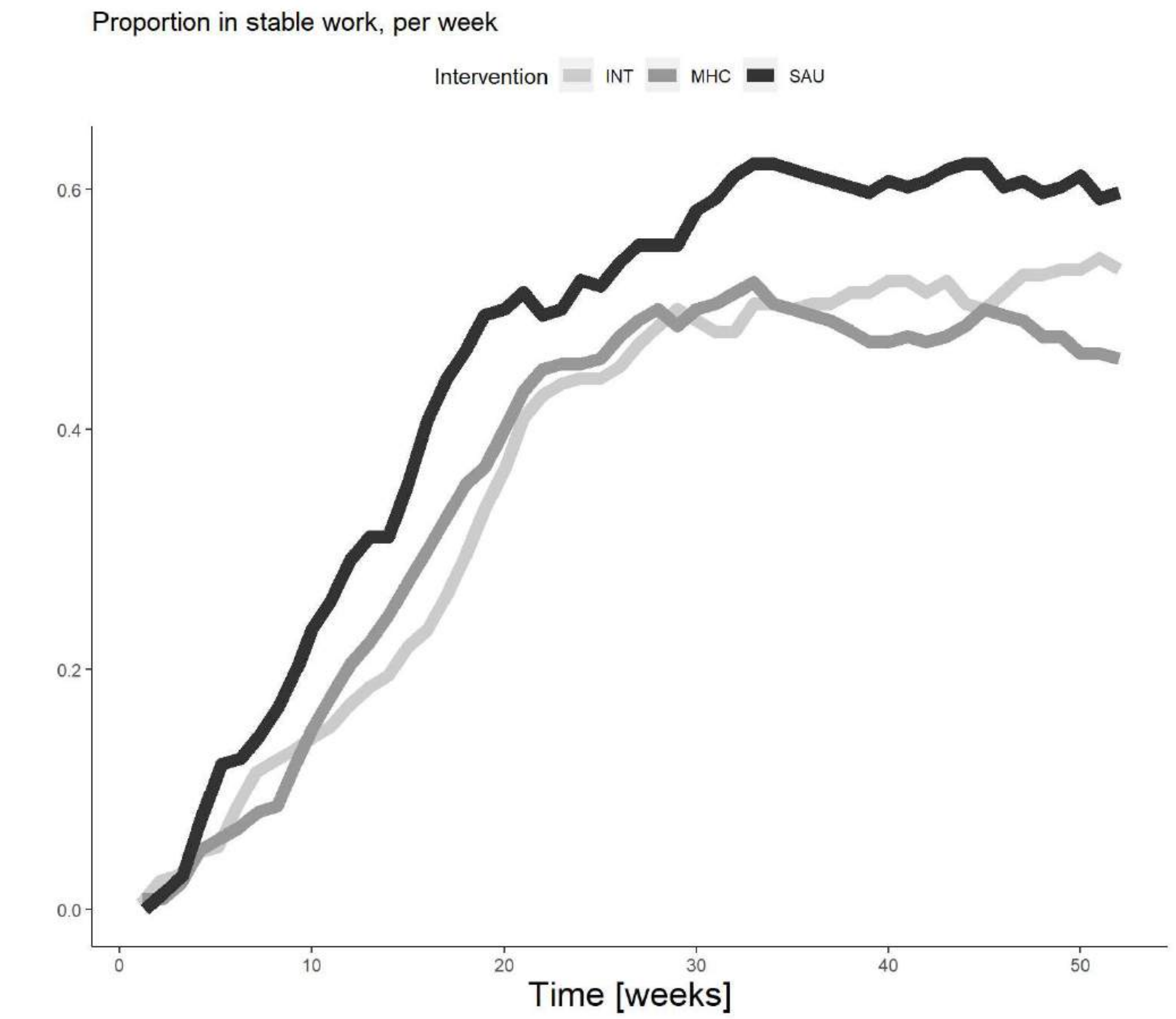

Vocational outcomes at 12-month follow-up from subgroup >intteams< in RCT2

	Group values			SAU-MHC					SAU-INT					MHC-INT				
	**INT**	**MHC**	**SAU**	**Est.**	**p**	**low CI**	**:**	**high CI**	**Est.**	**p**	**low CI**	**:**	**high CI**	**Est.**	**p**	**low CI**	**:**	**high CI**
RTW, FU12 [weeks, HR]	25.0	23.0	19	1.27	0.11	0.89		1.80	1.39	*0.0288	0.97		2.00	1.11	0.50	0.77		1.60
RTW, FU12 [weeks, HR]:teamnorth				1.17	0.48				1.07	0.76				0.90	0.67			
RTW, FU6 [HR]				1.30	0.11	0.88		1.92	1.48	*0.0203	0.99		2.24	1.15	0.42	0.76		1.75
RTW, FU6 [HR]:teamnorth				1.22	0.43				1.04	0.88				0.85	0.55			
PROP, FU12 [%, OR]	53.8	46.5	60	1.79	*0.0246	0.96		3.35	1.46	0.15	0.77		2.78	0.81	0.42	0.44		1.51
PROP, FU12 [%, OR]:teamnorth				-0.05	0.9				-0.39	0.36				-0.35	0.41			
WEEKS, FU12 [weeks, RR]	19.4	19.3	24	1.22	*0.0394	0.97		1.54	1.21	0.06	0.95		1.55	1.00	0.99	0.77		1.29
WEEKS, FU12 [weeks, RR]:teamnorth				0.03	0.83				0.00	0.99				-0.03	0.86			

Analyses regarding: >first<

Kaplan Meier-curve at 12-month follow-up regarding: first

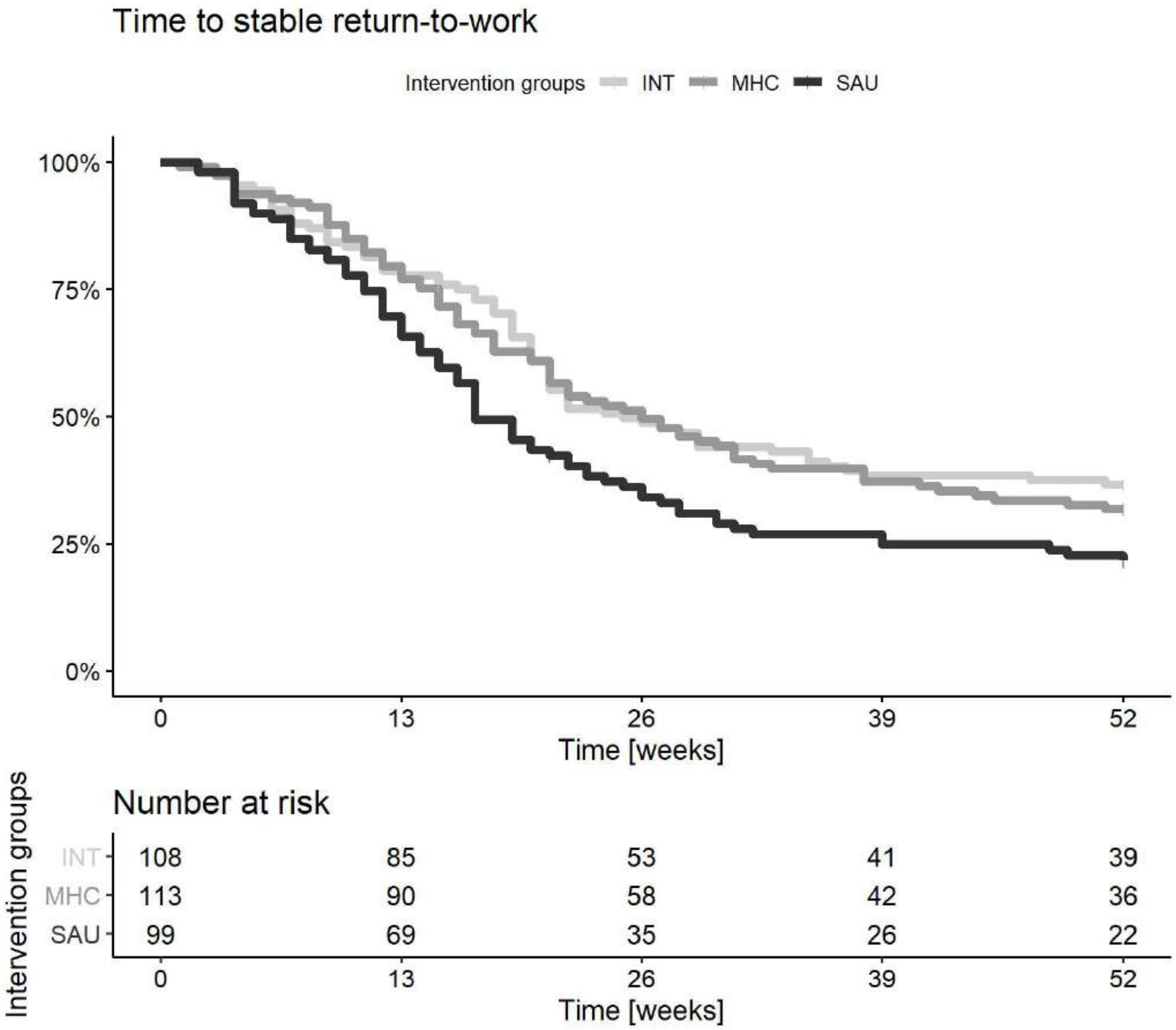

Proportion over time-curve at 12-month follow-up regarding: first<

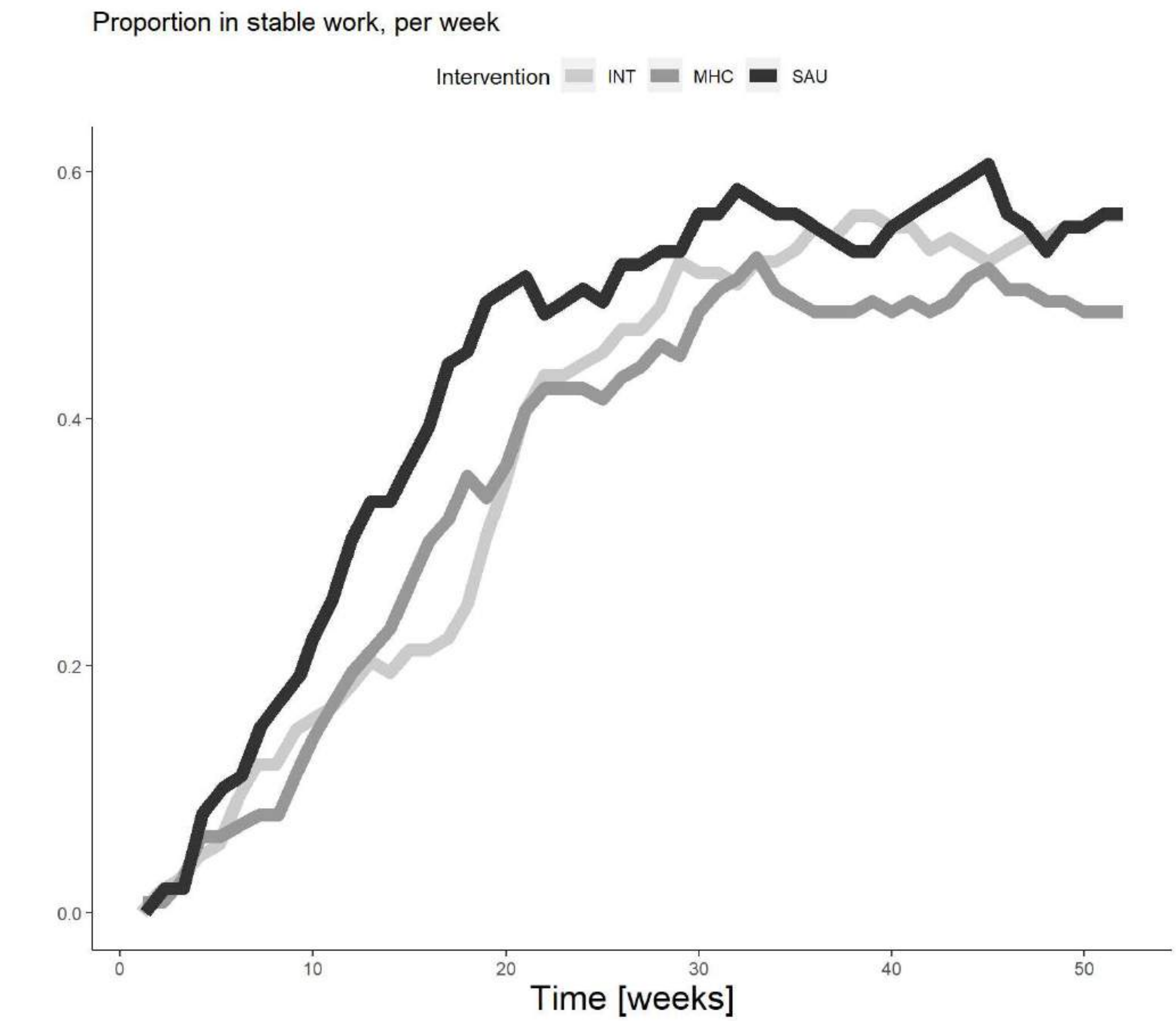

Vocational outcomes at 12-month follow-up from subgroup >first< in RCT2

	Group values			SAU-MHC				SAU-INT				MHC-INT			
	INT	MHC	SAU	Est.	p	low CI :	high CI	Est.	p	low CI :	high CI	Est.	p	low CI :	high CI
RTW, FU12 [weeks, HR]	25.0	26.0	17.0	1.45	*0.0235	0.98	2.15	1.48	*0.0198	0.99	2.21	1.01	0.95	0.67	1.51
RTW, FU6 [HR]				1.60	**0.0098	1.03	2.50	1.51	*0.0263	0.97	2.35	0.94	0.74	0.59	1.49
PROP, FU12 [%, OR]	57.0	49.1	57.1	1.40	0.24	0.70	2.80	0.90	0.74	0.43	1.90	0.66	0.15	0.33	1.32
WEEKS, FU12 [weeks, RR]	20.1	19.2	22.9	1.19	0.1	0.92	1.53	1.09	0.4	0.85	1.40	0.92	0.45	0.71	1.20

Self-reported outcomes at 6- and 12-month follow-up regarding: first

	Group values						SAU-MHC				SAU-INT				MHC-INT			
	INT	(SD)	MHC	(SD)	SAU	(SD)	Est.	p	low CI	high CI	Est.	p	low CI	high CI	Est.	p	low CI	high CI
BAI [FU6]	9.85	(7.77)	7.83	(6.5)	11.66	(8.3)	3.03	**0.0059	0.4	5.66	1.53	0.18	-1.2	4.26	-1.45	0.17	-4	1.1
BDI [FU6]	10.55	(8.75)	9.22	(8.63)	11.72	(8.73)	1.95	0.12	-1.07	4.97	1.03	0.39	-1.87	3.93	-0.87	0.46	-3.71	1.97
PSS [FU6]	16.01	(6.73)	13.73	(6.34)	16.34	(7.39)	2.05	*0.0264	-0.16	4.27	0.22	0.81	-1.96	2.4	-1.79	*0.0362	-3.83	0.26
KES [FU6]	61.91	(17.09)	57.87	(16.87)	65.69	(19.08)	6.45	**0.0031	1.25	11.66	2.81	0.2	-2.44	8.06	-3.5	0.09	-8.37	1.38
DSQ-som. [FU6]	8.14	(7)	7.49	(5.3)	8.92	(7.23)	1.4	0.12	-0.74	3.54	0.88	0.36	-1.43	3.2	-0.51	0.54	-2.51	1.5

	Group values						SAU-MHC				SAU-INT				MHC-INT			
	INT	(SD)	MHC	(SD)	SAU	(SD)	Est.	p	low CI	high CI	Est.	p	low CI	high CI	Est.	p	low CI	high CI
DSQ-distr. [FU6]	10.96	(7.48)	9.88	(6.55)	12.64	(8.39)	2.29	*0.0181	-0.03	4.62	1.28	0.19	-1.08	3.64	-1	0.27	-3.16	1.17
DSQ-anx. [FU6]	2.31	(3.89)	1.99	(3.8)	3.05	(4.03)	0.94	0.08	-0.36	2.25	0.64	0.22	-0.61	1.9	-0.27	0.61	-1.52	0.99
DSQ-depr. [FU6]	1.46	(2.68)	1.01	(1.99)	1.32	(2.05)	0.2	0.52	-0.54	0.94	-0.17	0.58	-0.93	0.58	-0.37	0.26	-1.15	0.42
WSAS [FU6]	12.36	(9.69)	10.56	(9.58)	13.46	(9.68)	2.27	0.08	-0.85	5.38	0.63	0.62	-2.46	3.72	-1.61	0.18	-4.48	1.25
SPS [FU6]	19.79	(2.36)	19.48	(2.63)	19.86	(2.96)	0.13	0.68	-0.62	0.89	0.12	0.68	-0.6	0.85	0	0.99	-0.67	0.66
IPQ [FU6]	17.54	(3.7)	17.07	(3.74)	17.13	(3.12)	0.12	0.81	-1.13	1.38	-0.29	0.58	-1.56	0.97	-0.41	0.43	-1.65	0.83
GSS [FU6]	27.65	(6.62)	28.72	(6.89)	28.93	(5.77)	-0.03	0.97	-2.16	2.1	1.16	0.2	-1.02	3.33	1.2	0.17	-0.9	3.31
RTW-SE [FU6]	25.76	(9.87)	27.22	(9.37)	25.49	(10.23)	-1.4	0.27	-4.45	1.64	-0.06	0.96	-3.08	2.96	1.27	0.28	-1.57	4.12
QoLs [FU6]	73.36	(13.67)	76.51	(13.64)	74.33	(14.22)	-1.17	0.53	-5.66	3.31	0.93	0.61	-3.44	5.29	2.13	0.22	-2.04	6.3
EQ5 [FU6]	0.83	(0.15)	0.85	(0.13)	0.83	(0.13)	-0.02	0.33	-0.06	0.03	0	0.96	-0.04	0.04	0.02	0.32	-0.03	0.06
IS [FU6]	25.43	(5.03)	24.99	(5.37)	21.61	(6.08)	-2.66	**0.0022	-4.72	-0.6	-2.99	**5e-04	-5.01	-0.96	-0.46	0.54	-2.28	1.35
BAI [FU12]	9.14	(7.79)	7.45	(5.69)	10.14	(9.06)	1.87	0.08	-0.7	4.43	0.49	0.66	-2.24	3.23	-1.32	0.21	-3.84	1.2
BDI [FU12]	8.58	(7.47)	8.23	(8.05)	7.92	(6.32)	-0.45	0.71	-3.37	2.48	-0.67	0.57	-3.5	2.16	-0.16	0.89	-2.99	2.67
PSS [FU12]	14.25	(6.69)	14.36	(6.7)	14.07	(6.44)	-0.32	0.73	-2.56	1.93	-0.31	0.75	-2.63	2.01	0.05	0.96	-2.17	2.27
KES [FU12]	54.01	(16.75)	51.15	(15.33)	56.92	(16.52)	4.93	*0.0258	-0.37	10.23	2.32	0.29	-2.99	7.63	-2.43	0.25	-7.54	2.69
DSQ-som. [FU12]	7.27	(5.72)	6.72	(4.8)	8.63	(6.92)	1.47	0.09	-0.63	3.56	0.92	0.33	-1.35	3.19	-0.51	0.54	-2.51	1.48
DSQ-distr. [FU12]	10.07	(6.77)	9.63	(6.36)	10.18	(6.8)	0.75	0.44	-1.55	3.04	0.17	0.86	-2.17	2.51	-0.55	0.55	-2.78	1.68
DSQ-anx. [FU12]	1.91	(3.12)	1.3	(2.69)	2.29	(3.63)	0.8	0.12	-0.43	2.03	0.25	0.63	-0.97	1.47	-0.5	0.31	-1.7	0.69
DSQ-depr. [FU12]	1.17	(2.61)	0.9	(1.69)	0.82	(1.65)	-0.13	0.67	-0.83	0.58	-0.27	0.4	-1.03	0.49	-0.13	0.68	-0.91	0.64
WSAS [FU12]	11	(10.31)	9.93	(8.73)	10.24	(8.78)	0.23	0.86	-2.85	3.32	-0.87	0.52	-4.08	2.35	-1.04	0.41	-4.08	2
SPS [FU12]	19.73	(3.45)	18.95	(2.98)	19.05	(3.68)	0.03	0.93	-0.9	0.97	-0.32	0.4	-1.21	0.58	-0.31	0.37	-1.15	0.53
IPQ [FU12]	17.82	(3.71)	17.33	(4.11)	17.06	(3.61)	-0.09	0.87	-1.41	1.24	-0.6	0.28	-1.94	0.75	-0.49	0.36	-1.79	0.81
GSS [FU12]	28.46	(6.65)	30.06	(6.02)	30.43	(6.83)	0.34	0.7	-1.77	2.44	1.86	*0.0399	-0.31	4.02	1.56	0.07	-0.53	3.66
RTW-SE [FU12]	28.54	(9.01)	29.08	(8.91)	28.68	(8.79)	-0.17	0.89	-3.15	2.81	0.62	0.62	-2.38	3.62	0.74	0.54	-2.18	3.66
QoLs [FU12]	76.38	(12.57)	75.88	(12.39)	76.88	(12.17)	0.42	0.81	-3.89	4.74	0.65	0.72	-3.65	4.94	0.25	0.88	-3.85	4.35
EQ5 [FU12]	0.83	(0.14)	0.84	(0.16)	0.84	(0.13)	0	0.98	-0.05	0.05	0.01	0.49	-0.03	0.06	0.01	0.51	-0.03	0.06

Analyses regarding: >last<

Kaplan Meier-curve at 12-month follow-up regarding: last

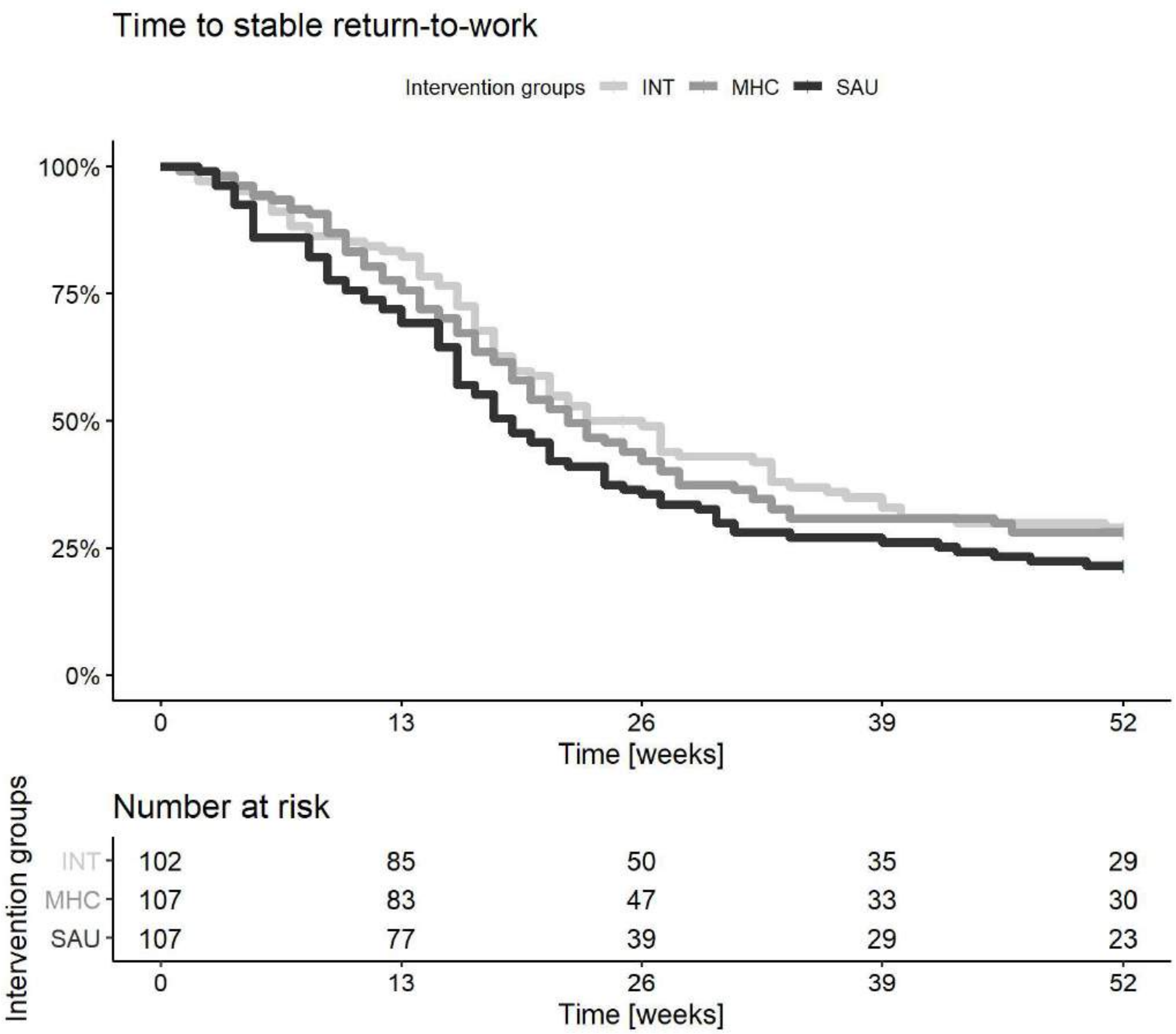

Proportion over time-curve at 12-month follow-up regarding: last<

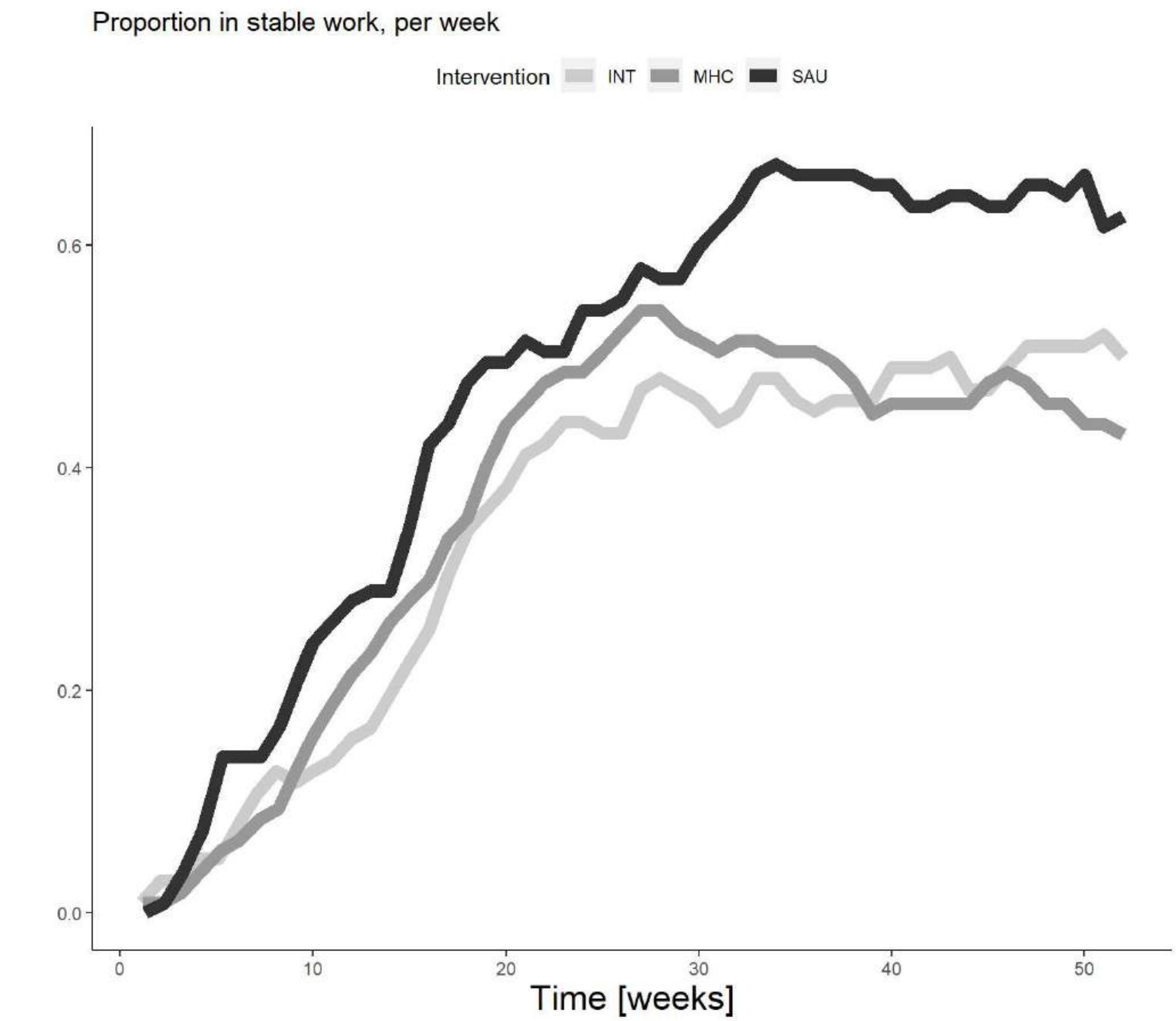

Vocational outcomes at 12-month follow-up from subgroup >last< in RCT2

	Group values			SAU-MHC				SAU-INT				MHC-INT			
	INT	MHC	SAU	Est.	p	low CI :	high CI	Est.	p	low CI :	high CI	Est.	p	low CI :	high CI
RTW, FU12 [weeks, HR]	26.0	22.0	19.0	1.30	0.11	0.88	1.91	1.42	*0.0314	0.96	2.10	1.10	0.58	0.74	1.63
RTW, FU6 [HR]				1.28	0.16	0.84	1.96	1.54	*0.0198	0.98	2.41	1.20	0.33	0.76	1.89
PROP, FU12 [%, OR]	50.5	43.8	62.6	2.22	**0.0059	1.10	4.46	1.67	0.08	0.82	3.40	0.75	0.34	0.37	1.53
WEEKS, FU12 [weeks, RR]	18.8	19.4	24.9	1.28	**0.0114	1.01	1.62	1.33	**0.004	1.05	1.70	1.05	0.67	0.81	1.36

Self-reported outcomes at 6- and 12-month follow-up regarding: last

	Group values						SAU-MHC				SAU-INT				MHC-INT			
	INT	(SD)	MHC	(SD)	SAU	(SD)	Est.	p	low CI	high CI	Est.	p	low CI	high CI	Est.	p	low CI	high CI
BAI [FU6]	9.21	(7.18)	8.62	(7.61)	10.33	(7.52)	1.47	0.17	-1.08	4.01	1.06	0.33	-1.58	3.69	-0.43	0.69	-3.03	2.16
BDI [FU6]	10.52	(8.9)	9.5	(8.01)	10.56	(8.63)	1.04	0.39	-1.85	3.93	0.19	0.88	-2.74	3.11	-0.82	0.5	-3.72	2.08
PSS [FU6]	15.03	(7.47)	13.82	(7.42)	15.69	(6.52)	1.75	0.06	-0.47	3.97	0.8	0.39	-1.44	3.04	-1	0.28	-3.22	1.23
KES [FU6]	60.76	(17.3)	59.91	(17.09)	65.29	(17.25)	4.4	*0.0332	-0.55	9.36	3.52	0.09	-1.53	8.57	-0.87	0.68	-5.9	4.16
DSQ-som. [FU6]	7.45	(5.12)	7.45	(5.8)	9.1	(6.77)	1.26	0.14	-0.79	3.32	1.18	0.16	-0.86	3.23	-0.16	0.84	-2.11	1.79

	Group values						SAU-MHC				SAU-INT				MHC-INT			
	**INT**	**(SD)**	**MHC**	**(SD)**	**SAU**	**(SD)**	**Est.**	**p**	**low CI**	**high CI**	**Est.**	**p**	**low CI**	**high CI**	**Est.**	**p**	**low CI**	**high CI**
DSQ-distr. [FU6]	10.09	(6.93)	10.66	(7.39)	11.3	(7.13)	0.7	0.45	-1.51	2.9	1.04	0.26	-1.15	3.22	0.33	0.71	-1.87	2.54
DSQ-anx. [FU6]	1.94	(3.1)	2.08	(3.1)	2.53	(3.59)	0.51	0.32	-0.73	1.74	0.5	0.35	-0.78	1.77	-0.03	0.95	-1.21	1.15
DSQ-depr. [FU6]	1.12	(2.26)	1.15	(2.05)	1.17	(2.12)	0.11	0.71	-0.63	0.86	0.1	0.76	-0.67	0.86	-0.02	0.94	-0.75	0.71
WSAS [FU6]	11.27	(9.34)	10.36	(9.5)	13.02	(10.32)	2.1	0.1	-1.01	5.2	1.55	0.22	-1.51	4.62	-0.66	0.6	-3.67	2.34
SPS [FU6]	19.46	(2.58)	19.47	(3.35)	19.95	(2.35)	0.13	0.68	-0.62	0.89	0.12	0.68	-0.6	0.85	0	0.99	-0.67	0.66
IPQ [FU6]	17.58	(3.48)	17.51	(3.79)	17.15	(4.21)	-0.32	0.54	-1.61	0.96	-0.33	0.54	-1.64	0.97	0.04	0.94	-1.21	1.29
GSS [FU6]	29.26	(6.64)	29.66	(6.39)	28.84	(6.24)	-0.62	0.49	-2.78	1.54	-0.17	0.85	-2.4	2.05	0.43	0.64	-1.75	2.6
RTW-SE [FU6]	27.53	(9.15)	26.83	(8.82)	27.04	(8.48)	0.38	0.75	-2.42	3.17	-0.51	0.66	-3.28	2.26	-0.74	0.52	-3.5	2.02
QoLs [FU6]	75.07	(14.3)	73.63	(14.14)	75	(14.57)	0.82	0.65	-3.55	5.2	-0.12	0.95	-4.6	4.37	-0.94	0.6	-5.28	3.41
EQ5 [FU6]	0.83	(0.13)	0.84	(0.12)	0.82	(0.15)	-0.02	0.33	-0.06	0.03	-0.01	0.64	-0.05	0.03	0.01	0.54	-0.03	0.05
IS [FU6]	25.49	(5.36)	25.49	(4.49)	21.82	(5.68)	-2.91	**4e-04	-4.86	-0.95	-2.87	**9e-04	-4.91	-0.82	0.11	0.89	-1.8	2.02
BAI [FU12]	7.9	(6.12)	8.92	(8.02)	9.97	(8.16)	0.9	0.4	-1.65	3.44	1.66	0.11	-0.84	4.17	0.79	0.45	-1.72	3.3
BDI [FU12]	9.6	(8.59)	9.34	(9.84)	9.05	(9.06)	0.19	0.88	-2.73	3.11	-0.29	0.81	-3.21	2.63	-0.38	0.75	-3.28	2.53
PSS [FU12]	13.43	(7.71)	13.7	(8.2)	15.06	(6.57)	1.15	0.23	-1.16	3.46	1.38	0.16	-0.95	3.7	0.22	0.82	-2.15	2.59
KES [FU12]	53.39	(17.98)	53.09	(19.83)	56.12	(18.34)	2.6	0.24	-2.68	7.87	2.4	0.28	-2.94	7.74	-0.08	0.97	-5.48	5.32
DSQ-som. [FU12]	7.32	(5.66)	7.85	(6.9)	8.5	(6.44)	0.45	0.61	-1.66	2.56	0.95	0.26	-1.08	2.99	0.45	0.59	-1.54	2.45
DSQ-distr. [FU12]	9.87	(6.93)	10.42	(8.55)	10.78	(6.87)	0.25	0.79	-2.03	2.53	0.79	0.39	-1.42	3	0.57	0.55	-1.74	2.88
DSQ-anx. [FU12]	1.7	(2.82)	2.16	(3.37)	2.41	(3.71)	0.17	0.74	-1.03	1.36	0.62	0.21	-0.57	1.81	0.45	0.34	-0.68	1.58
DSQ-depr. [FU12]	0.95	(1.68)	1.48	(2.99)	0.88	(1.88)	-0.49	0.12	-1.25	0.27	-0.07	0.82	-0.78	0.64	0.43	0.17	-0.32	1.18
WSAS [FU12]	9.28	(8.5)	10.07	(10.15)	9.96	(9.98)	0.02	0.99	-3.17	3.21	0.59	0.65	-2.49	3.68	0.49	0.7	-2.53	3.51
SPS [FU12]	18.92	(2.84)	17.87	(3.82)	19.07	(3.87)	0.03	0.93	-0.9	0.97	-0.32	0.4	-1.21	0.58	-0.31	0.37	-1.15	0.53
IPQ [FU12]	17.52	(4.17)	17.82	(4.17)	17.78	(3.41)	0.01	0.99	-1.33	1.35	0.12	0.83	-1.23	1.48	0.18	0.75	-1.14	1.49
GSS [FU12]	30.02	(6.91)	30.51	(6.51)	29.79	(6.59)	-0.18	0.84	-2.35	1.98	0.03	0.97	-2.19	2.25	0.19	0.83	-2.01	2.4
RTW-SE [FU12]	29.19	(9.03)	28.94	(9.44)	28.23	(8.62)	-0.18	0.88	-3.04	2.68	-0.76	0.53	-3.65	2.12	-0.47	0.7	-3.34	2.4
QoLs [FU12]	76.49	(14.45)	74.7	(16.77)	77.47	(13.11)	2	0.29	-2.5	6.5	0.46	0.81	-4.01	4.92	-1.55	0.41	-6.12	3.01
EQ5 [FU12]	0.84	(0.13)	0.85	(0.13)	0.83	(0.13)	-0.02	0.3	-0.06	0.02	-0.01	0.48	-0.06	0.03	0.01	0.71	-0.03	0.05

Analyses regarding: >inttemp<

Kaplan Meier-curve at 12-month follow-up regarding: inttemp

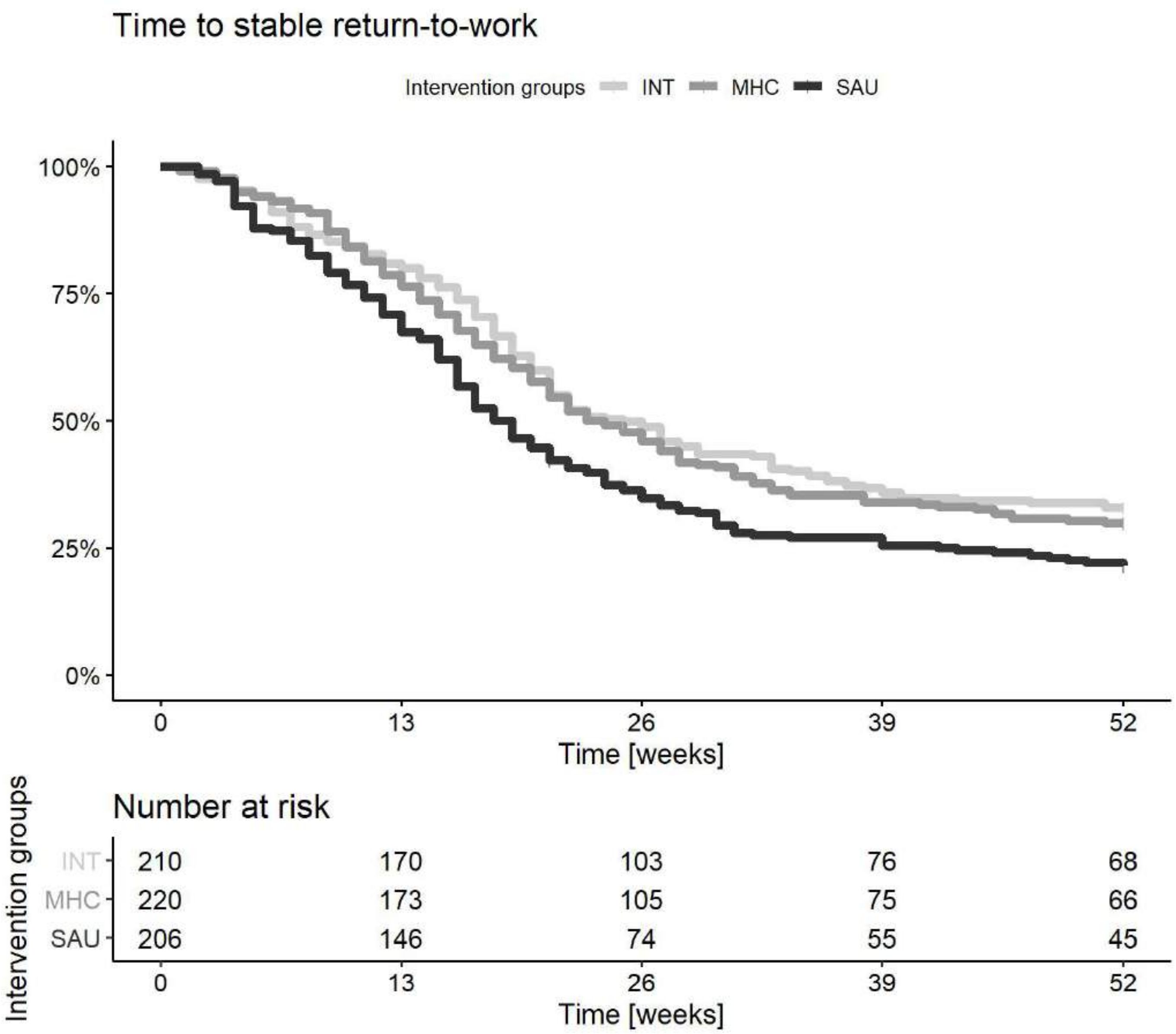

Proportion over time-curve at 12-month follow-up regarding: inttemp<

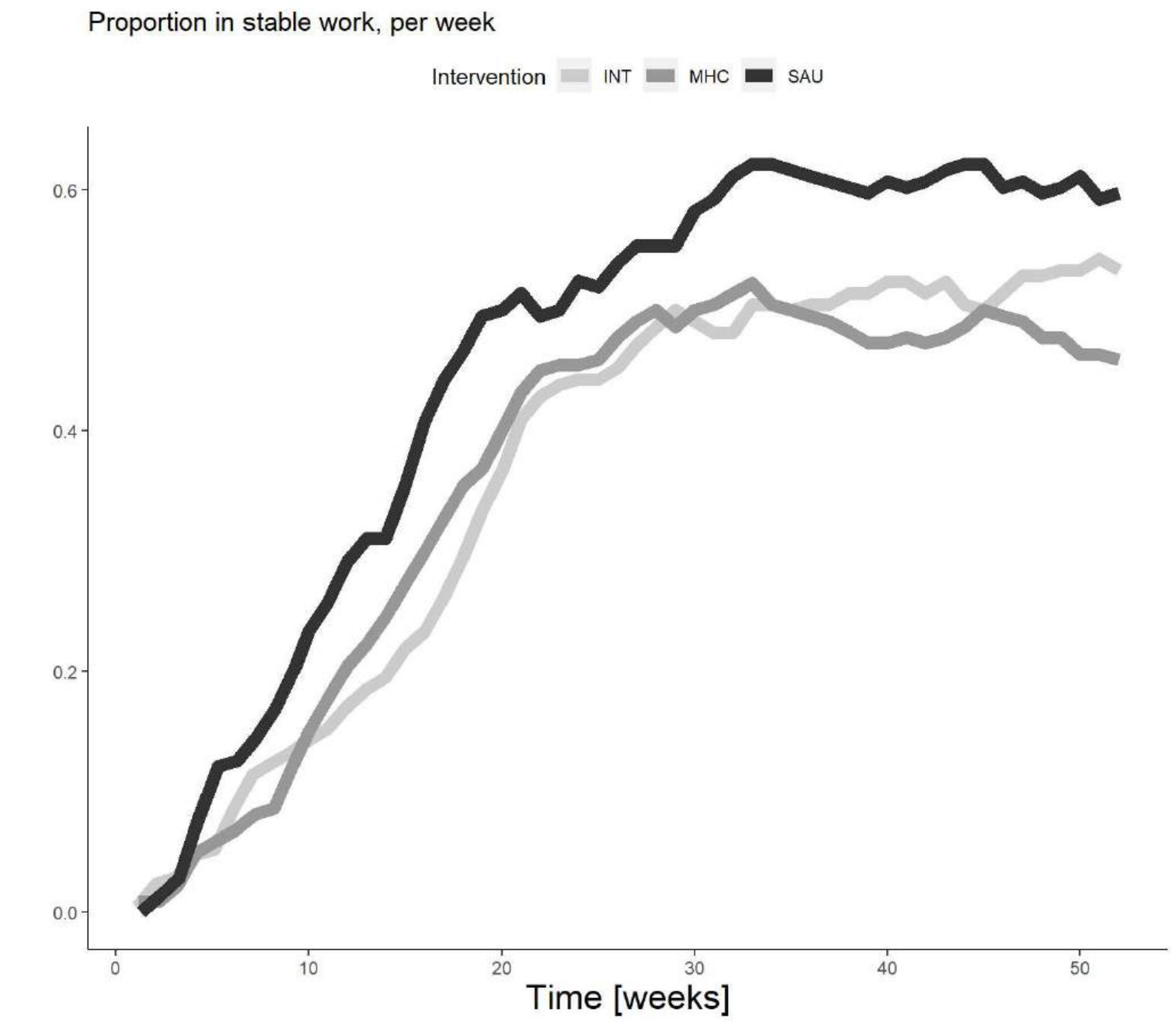

Vocational outcomes at 12-month follow-up from subgroup >inttemp< in RCT2

	Group values			SAU-MHC				SAU-INT				MHC-INT			
	INT	MHC	SAU	Est.	p	low CI :	high CI	Est.	p	low CI :	high CI	Est.	p	low CI :	high CI
RTW, FU12 [weeks, HR]	25.0	23.0	19	1.41	*0.0343	0.95	2.08	1.48	*0.0193	0.99	2.21	1.04	0.80	0.70	1.56
RTW, FU12 [weeks, HR]:temphalflast				0.92	0.7			0.94	0.78			1.04	0.86		
RTW, FU6 [HR]				1.54	*0.0173	1.00	2.39	1.49	*0.0301	0.96	2.32	0.97	0.85	0.61	1.52
RTW, FU6 [HR]:temphalflast				0.83	0.47			1.03	0.91			1.24	0.42		
PROP, FU12 [%, OR]	53.8	46.5	60	1.39	0.24	0.70	2.76	0.94	0.83	0.46	1.92	0.67	0.15	0.34	1.32
PROP, FU12 [%, OR]:temphalflast				0.46	0.25			0.58	0.17			0.14	0.73		
WEEKS, FU12 [weeks, RR]	19.4	19.3	24	1.19	0.1	0.92	1.54	1.11	0.33	0.86	1.43	0.93	0.52	0.72	1.21
WEEKS, FU12 [weeks, RR]:temphalflast				0.07	0.64			0.18	0.21			0.12	0.44		
